# Biosynthetic and catabolic pathways control amino acid δ^2^H values in aerobic heterotrophs

**DOI:** 10.3389/fmicb.2024.1338486

**Published:** 2024-04-05

**Authors:** Shaelyn N. Silverman, Reto S. Wijker, Alex L. Sessions

**Affiliations:** Division of Geological and Planetary Sciences, California Institute of Technology, Pasadena, CA, United States

**Keywords:** amino acids, hydrogen isotopes, isotope fractionation, aerobic metabolism, heterotrophic bacteria, pyruvate, NADPH

## Abstract

The hydrogen isotope ratios (δ^2^H_AA_ values) of amino acids in all organisms are substantially fractionated relative to growth water. In addition, they exhibit large variations within microbial biomass, animals, and human tissues, hinting at rich biochemical information encoded in such signals. In lipids, such δ^2^H variations are thought to primarily reflect NADPH metabolism. Analogous biochemical controls for amino acids remain largely unknown, but must be elucidated to inform the interpretation of these measurements. Here, we measured the δ^2^H values of amino acids from five aerobic, heterotrophic microbes grown on different carbon substrates, as well as five *Escherichia coli* mutant organisms with perturbed NADPH metabolisms. We observed similar δ^2^H_AA_ patterns across all organisms and growth conditions, which–consistent with previous hypotheses–suggests a first-order control by biosynthetic pathways. Moreover, δ^2^H_AA_ values varied systematically with the catabolic pathways activated for substrate degradation, with variations explainable by the isotopic compositions of important cellular metabolites, including pyruvate and NADPH, during growth on each substrate. As such, amino acid δ^2^H values may be useful for interrogating organismal physiology and metabolism in the environment, provided we can further elucidate the mechanisms underpinning these signals.

## 1 Introduction

Stable hydrogen isotope analysis of amino acids (δ^2^H_AA_) is receiving growing attention due to its potential utility as a tracer of ecological and/or physiological processes, as well as the extreme fractionations recorded in laboratory-grown and natural organisms. In the first published study on terrestrial δ^2^H_AA_ values, Fogel et al. (2016) discovered large (>100‰) variations in δ^2^H_AA_ values in *Escherichia coli* cultured on glucose or tryptone (a complex protein source) in different growth waters, with two key insights emerging from their study: (1) patterns of δ^2^H_AA_ values may be driven by ubiquitous biochemical mechanisms associated with amino acid synthesis in organisms, and (2) hydrogen can be directly routed from organic substrates, or incorporated from water via de novo amino acid synthesis, to variable extents depending on the protein content of the medium. Expanding this work to an animal model, Newsome et al. (2020) observed that hydrogen sources of amino acids in mouse muscle tissue are driven by similar metabolic factors as *E. coli*, but that carbohydrates and amino acids from both the diet and gut microbiome are particularly important hydrogen sources. Recently, Gharibi et al. (2022a) reported extreme ^2^H-enrichments in proline and hydroxyproline (δ^2^H values >1,000‰) from seal bone collagen, although the cause of these extreme δ^2^H values was not identified. Smith et al. (2022) ruled out growth rate as a primary control on δ^2^H_AA_ values in *E. coli* and revealed that carbon and hydrogen isotope compositions of amino acids are governed by different biochemical factors. Drawing on the well-known spatial variations in precipitation isotope ratios (Craig, 1961; Dansgaard, 1964; Rozanski et al., 1993; Kendall and Coplen, 2001; Poage and Chamberlain, 2001), Mancuso et al. (2023) revealed the first systematic link between δ^2^H_AA_ values in human tissue (scalp hair) and local water δ^2^H, supporting the utility of this compound-specific tool as a potential tracer of geographical origin (Rubenstein and Hobson, 2004; Bowen et al., 2005). Together, these results encourage a variety of potential exciting applications of δ^2^H_AA_ analysis across diverse fields such as ecology, archaeology, microbiology, biogeochemistry, and forensics. However, these applications are limited by our lack of fundamental understanding of which biochemical controls set δ^2^H_AA_ values in terrestrial organisms.

**Figure 1:**
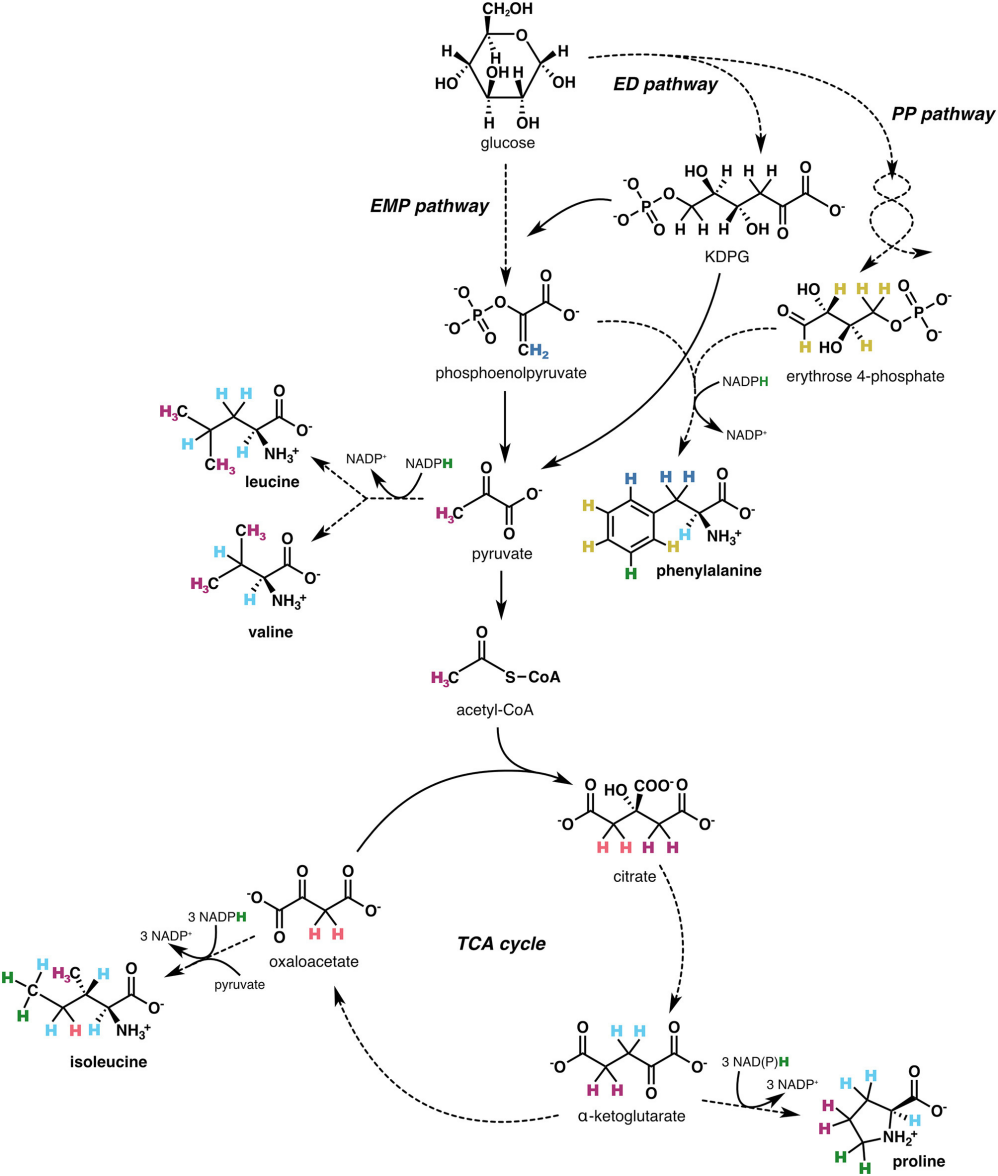
Simplified schematic of biosynthetic pathways (showing end-member compounds), central metabolic pathway precursors, and hydrogen sources for the five amino acids investigated in this study (proline, phenylalanine, leucine, valine, isoleucine). Hydrogen atoms are visually tracked from source to amino acid through colors: green indicates hydrogen from NAD(P)H, light blue from water, and the remaining colors correspond to hydrogen from organic precursors. Solid arrows denote single metabolic reactions; dashed arrows encompass multiple steps. Although NAD(P)H is used to reduce substrates in all amino acid biosynthetic pathways, some NAD(P)H-derived hydrogen is subsequently lost due to elimination or equilibration with water (see detailed biosynthetic pathways in Supplementary Figures S16–S19). EMP, Embden-Meyerhof-Parnas; ED, Entner-Doudoroff; PP, pentose phosphate; TCA, tricarboxylic acid.

Here we seek to elucidate some of the mechanistic controls on biological δ^2^H_AA_ values. We focus on microbes, which are simpler systems than animals because most microbes are unicellular, can synthesize all 20 amino acids (Price et al., 2018), and can be grown in defined media. Furthermore, microbes are the major drivers of biogeochemical processes such as energy and nutrient cycling in the environment (Falkowski et al., 2008), so understanding how their δ^2^H_AA_ values relate to their metabolic activities may render δ^2^H_AA_ analysis a useful tool for interrogating the critical microbial-driven changes to our planet's surface geochemistry. Amino acids are formed via biosynthetic pathways that are ubiquitous across most forms of life. Their carbon skeleton precursors are the intermediates of central metabolic pathways (Figure 1), and the hydrogen on each amino acid is derived from different combinations of sources, including the organic precursors, water, and NAD(P)H. As such, δ^2^H_AA_ values are complicated to interpret, but may contain multiple layers of useful biochemical information.

**Figure 2:**
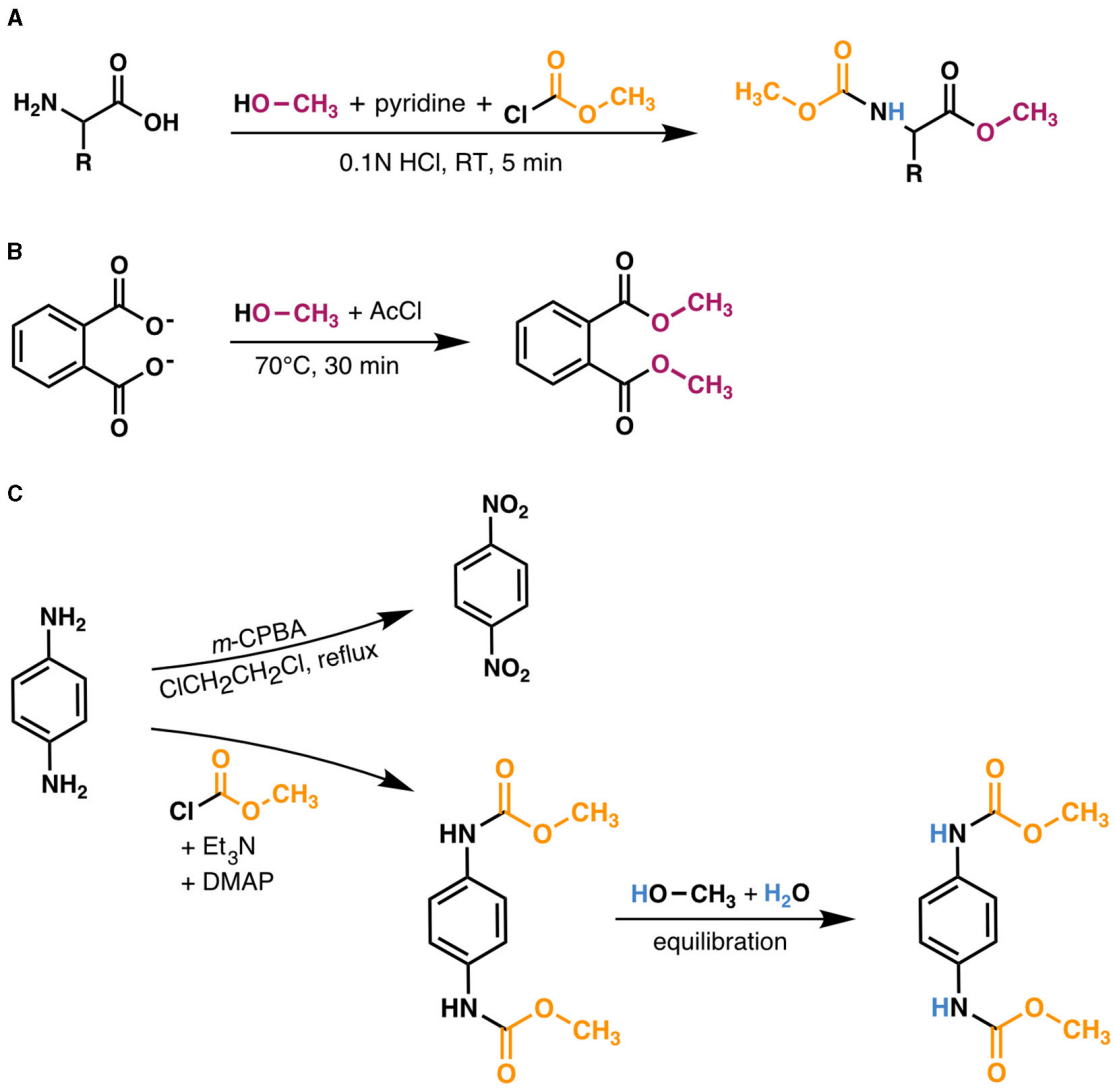
Derivatization scheme for amino acids and methods used to measure δ^2^H values of derivative reagents. **(A)** Amino acids were derivatized with anhydrous methanol, pyridine, and methyl chloroformate in 0.1N HCl; see Section 2.2 for details. The exchangeable amine hydrogen atom (blue in derivatized product) was equilibrated in the solvent (0.1N HCl), which was prepared using the same water supply as that used to equilibrate the N-bound hydrogen in the dimethyl 1,4-phenylenedicarbamate product [DCP; depicted in **(C)**]. **(B)** The δ^2^H value of anhydrous methanol was measured by derivatizing disodium phthalate with known isotopic composition (Sessions et al., 2002) with anhydrous methanol in acetyl chloride (AcCl); see Section 2.5 for details. **(C)** The δ^2^H value of methyl chloroformate was measured by first oxidizing *p*-phenylenediamine (PPD) to dinitrobenzene (DNB) to obtain the aromatic hydrogen δ^2^H value (top reaction), then separately derivatizing PPD with methyl chloroformate to produce the DCP (bottom reaction). DCP was purified, then dissolved in a 1:1 (v/v) mixture of anhydrous methanol:water to equilibrate the N-bound hydrogen atoms before extraction and measurement via GC/P/IRMS. See Section 2.5 for details.

In this study, we investigated the δ^2^H values of amino acids from the biomass of five aerobic, heterotrophic bacteria that was previously generated for lipid δ^2^H analysis (Wijker et al., 2019). The organisms included *E. coli, Bacillus subtilis, Ensifer meliloti, Pseudomonas fluorescens*, and *Rhizobium radiobacter*, as well as five mutant *E. coli* organisms lacking specific dehydrogenase or transhydrogenase enzymes. These organisms were grown on different carbon substrates, including on glucose for which their metabolic fluxes were characterized (Wijker et al., 2019), enabling investigation of the mechanistic link between δ^2^H_AA_ values and microbial metabolism. This experimental system provides the opportunity to test a number of hypotheses about the mechanisms that govern amino acid δ^2^H values, including whether NADPH metabolism is a primary control (the case for lipids; Zhang et al., 2009; Wijker et al., 2019), and how varied fluxes through specific enzymes in central metabolism affect δ^2^H_AA_ values. We targeted five amino acids–proline, phenylalanine, leucine, valine, and isoleucine–which were selected because (1) they span different parts of central metabolism (Figure 1), and (2) their hydrogen isotope compositions are among the most reliable to interpret, as these amino acids exhibit consistent baseline chromatographic separation (Supplementary Figure S1), have relatively high ionization efficiencies, and maintain stable hydrogen isotope compositions through hydrolysis and derivatization (Supplementary Figures S9, S10; Supplementary Table S4; Silverman et al., 2022; for further details, see Supplementary Section 1). We provide hypotheses for the observed δ^2^H_AA_ patterns within and across organisms cultured under different conditions. As such, we aim to elucidate the underlying mechanisms that control ^2^H/^1^H fractionation in these five amino acids.

Additionally, δ^2^H_AA_ analyses to-date have been hampered by the presence of “labile” organic hydrogen in the amine (–NH_2_) and carboxyl (–COOH) groups, which readily exchange with hydrogen in both water and ambient water vapor. Derivatization of amino acids removes the carboxyl- and one amine-bound hydrogen, but the remaining amine hydrogen cannot be excluded from the measured isotopic composition, and may dilute or obscure biological signals (i.e., those of non-exchangeable, C-bound hydrogen in the amino acids) and furthermore lead to incomparable results across laboratories. Previous studies (Fogel et al., 2016; Newsome et al., 2020; Smith et al., 2022; Mancuso et al., 2023) have attempted to correct for the contribution of derivative and exchangeable hydrogen to measured δ^2^H_AA_ values through the use of amino acid standards, whereby the δ^2^H values of underivatized amino acid powders pre-equilibrated with ambient water vapor (following the comparative equilibration method; Wassenaar and Hobson, 2003) are measured via a high-temperature conversion elemental analyzer coupled to an isotope ratio mass spectrometer, then subtracted via mass balance from the δ^2^H values of corresponding derivatized amino acids. The central issue with this approach is that hydrogen in the derivative reagents, derivatized amino acids, and underivatized amino acids cannot be mass balanced, as (1) the isotopic fractionations between the exchangeable hydrogen and water (or ambient moisture) are unknown, thus the δ^2^H values of the carboxyl and amine hydrogen atoms removed during derivatization cannot be properly accounted for, and (2) the isotopic fractionation between the amine-bound hydrogen and water likely differs when amino acids are in derivatized (possessing a secondary amine) vs. underivatized (primary amine) form, so knowledge of the amine hydrogen δ^2^H value in the latter case may not help correct for exchangeable hydrogen in the former case. Independent measurements of the derivative reagent δ^2^H values are possible in some cases, but without accompanying correction for the amine-bound hydrogen in derivatized amino acids, errors in the reported δ^2^H values of amino acid carbon-bound hydrogen may be significant (on the order of 10 to 100‰; Supplementary Figure S7; Supplementary Section 4). Here we have developed a new, simple procedure for controlling this exchangeable amine-bound hydrogen based on separate oxidation and derivatization of a diamine compound to obtain the combined δ^2^H value of our amine group derivative and the exchangeable hydrogen. By subtracting the isotopic contribution of both the derivative hydrogen and exchangeable amine-bound hydrogen, we are able to accurately calculate the δ^2^H value of pure carbon-bound hydrogen in amino acids.

## 2 Materials and methods

### 2.1 Strain and culture conditions

The microbial biomass measured here was generated in a prior study targeting lipid δ^2^H analysis (Wijker et al., 2019); all relevant culturing details are recapitulated here. Five wildtype aerobic heterotrophic microbes (*Escherichia coli* MG1655, *Bacillus subtilis* PY79, *Ensifer meliloti* Young 2003, *Pseudomonas fluorescens* 2-79, and *Rhizobium radiobacter* C58) and five mutant *E. coli* organisms carrying specific deletions of dehydrogenase or transhydrogenase genes (glucose 6-phosphate dehydrogenase deleted in JW1841, phosphoglucose isomerase deleted in JW3985, membrane-bound transhydrogenase deleted in PntAB, soluble transhydrogenase deleted in UdhA, and both transhydrogenases deleted in UdhA-PntAB) were cultured on unlabeled glucose for hydrogen isotope analysis, and on ^13^C-labeled glucose (100% 1-^13^C-glucose and a mixture of 20% (wt/wt) U-^13^C_6_-glucose + 80% (wt/wt) unlabeled glucose) for metabolic flux analysis. The relative metabolic fluxes were calculated based on the ^13^C-labeling pattern of proteinogenic amino acids—see Wijker et al. (2019) for more details. Wildtype organisms were additionally cultured on acetate, citrate, fructose, pyruvate, and/or succinate in an isotopically constant growth water; as well as on glucose in growth waters with different isotopic compositions, which were manipulated by adding specific volumes of 99.9% purity D_2_O to distilled, deionized water. Each organism was grown with 4 g/L of carbon source in M9 minimal medium (prepared as described in Fuhrer et al., 2005) in batch culture on a rotary shaker at 200 rpm, thereby ensuring aerobic conditions were maintained and fermentation was avoided throughout the course of the experiments. The carbon source served as the limiting nutrient in each culture, causing cells to transition to stationary growth phase upon depletion. *B. subtilis* and *R. radiobacter* cultures were supplemented with a vitamin mixture, while strains JW1841 and JW3985 were given 50 μg/mL of kanamycin. *B. subtilis* and *E. coli* cultures were incubated at 37°C; all other organisms were incubated at 30°C. All wildtype cultures were prepared in duplicate except for organisms grown in D_2_O-spiked media (for growth water experiments) and for *E. coli*, which was grown on pyruvate and acetate in single cultures, and on glucose in two non-replicate cultures: culture #1 was grown along with the rest of the wildtype organisms, *E. coli* mutants, and growth water experiments for non-*E. coli* organisms; culture #2 was grown at a later date as one of four cultures in *E. coli* growth water experiments. Although culturing conditions were identical between *E. coli* cultures #1 and #2 grown on glucose, the different timing of culturing, and different methods used to process the biomass (see Section 2.2), renders culture #2 a repeat experiment, but not true biological replicate, to culture #1. Culture growth was monitored by measuring optical density at 600 nm (OD_600_), and cells were harvested in late-exponential phase, lyophilized, and stored at -80°C until further processing for lipid and amino acid δ^2^H analysis (Wijker et al., 2019 and this study, respectively).

### 2.2 Amino acid hydrolysis, derivatization, extraction, and quantification

A 10–20 mg of dry biomass from each sample was hydrolyzed anoxically in 6N HCl at 110°C for 24 h in tightly capped VOA vials. Following hydrolysis, samples were uncapped and left on the hot plates until the 6N HCl was completely evaporated, then samples were resuspended in 0.5 ml of 0.1N HCl. Amino acids in all samples except *E. coli* culture #2 were derivatized with 7:6:3 (v/v/v) anhydrous methanol (MeOH), pyridine, and methyl chloroformate (MCF); reagents were added at room temperature, then samples were immediately capped and sonicated for ~5 min (procedure adapted from Husek, 1991a,b and Zampolli et al., 2007). *E. coli* culture #2 was derivatized with the same reagent bottles and reaction procedure as the other samples, but was placed on dry ice while derivative reagents were added to slow the derivatization reaction. Note that in contrast to most published δ^2^H_AA_ studies (Fogel et al., 2016; Newsome et al., 2020; Smith et al., 2022; Mancuso et al., 2023), we avoid using fluorinated derivative reagents, as hydrofluoric acid can form during pyrolysis in the gas chromatograph/isotope ratio mass spectrometer (GC/IRMS), leading to potential hydrogen isotope fractionation (Sauer et al., 2001; Renpenning et al., 2017; Silverman et al., 2022).

The resulting methoxycarbonyl (MOC) esters (Figure 2A) were extracted twice with methyl tert-butyl ether (MTBE) and filtered through a sodium sulfate column to adsorb any water present. Samples were concentrated to ~0.25–0.5 ml under N_2_. MOC ester peaks were identified via gas chromatography/mass spectrometry (GC/MS) on a Thermo-Scientific Trace ISQ equipped with a Zebron ZB-5 ms column (30-m × 0.25-mm i.d., 0.25 μm film thickness) and programmable temperature vaporizing (PTV) injector operated in splitless mode, using He as a carrier gas (flow rate = 1.4 ml/min). The GC oven was held at 80°C for 1 min, ramped at 5°C/min to 280°C with no hold, then ramped at 20°C/min to 310°C with a final 5 min temperature hold. Peaks were identified by comparing the relative retention times and mass spectra to those of known MOC ester standards, as well as to mass spectra in the NIST MS Library database.

### 2.3 Isotope analysis

The δ^2^H values of MOC esters were measured by a gas chromatograph coupled to an isotope ratio mass spectrometer (Thermo Finnigan Delta^+^XP) using a pyrolysis interface (i.e., GC/P/IRMS). Chromatographic separation was achieved on a thick-film Zebron ZB-5ms column (30-m × 0.25-mm i.d., 1.00 μm film thickness) with a nearly identical chromatographic method as used in GC/MS analysis [exceptions included a higher carrier gas flow rate (1.7 ml/min) and slight modifications to the temperature program to optimize MOC ester separation] so peaks could be identified by retention order and relative height. Measured isotope ratios were calibrated using hydrogen gas of known isotopic composition and are reported in δ notation (in units of ‰, or parts per thousand; Urey, 1948; McKinney et al., 1950) relative to the Vienna Standard Mean Ocean Water (VSMOW) international standard (δ^2^H = *R*_AA_/*R*_VSMOW_ − 1), where *R* = ^2^H/^1^H. Additionally, an eight-compound fatty acid methyl ester standard mixture was analyzed between every 5–6 samples to verify instrument accuracy and precision. Samples were analyzed in triplicate, and the MOC ester δ^2^H values were corrected for the addition of methyl hydrogen from the derivative reagents, as well as for the remaining exchangeable amine hydrogen (see Section 2.5). The standard deviation of triplicate analyses for individual amino acids was typically ≤6‰. The average root-mean-square error of the external FAME standard was 3.2‰ across all analyses. δ^2^H values of the culture media (δ^2^H_w_) were measured previously using a Los Gatos Research DLT-100 liquid water isotope analyzer and calibrated against up to four working standards with δ^2^H values ranging from –73 to +458‰ (Wijker et al., 2019). Data are reported as apparent fractionations between amino acids (AA) and culture medium water (w) according to the equation ${\;}^{2}\varepsilon_{\text{AA/w}}$ = (δ^2^H_AA_ + 1)/(δ^2^H_w_ + 1) − 1, with uncertainty propagated as Equation 1


(1) $$\begin{array}{l} \sigma_{\varepsilon }=\left(\frac{\delta^{2}{\text{H}}_{\text{AA}}+1}{\delta^{2}{\text{H}}_{\text{w}}+1}\right)\sqrt{{\left(\frac{\sigma_{\text{AA}}}{\delta^{2}{\text{H}}_{\text{AA}}+1}\right)}^{2}+{\left(\frac{\sigma_{\text{w}}}{\delta^{2}{\text{H}}_{\text{w}}+1}\right)}^{2}} \end{array}$$

### 2.4 Hydrolysis and derivatization tests for isotopic alteration

Potential changes in amino acid isotopic compositions during acid hydrolysis were investigated by varying the hydrolysis conditions used (temperature, duration, and O_2_ presence). For a control treatment, standard bovine serum albumin (BSA) was hydrolyzed in 6N HCl for 24 h at 110°C under anoxic conditions (achieved by sparging samples with N_2_ for 2 min with vigorous shaking). Variations on these conditions were achieved by either hydrolyzing BSA (1) without sparging with N_2_ (oxic hydrolysis), (2) at 105°C, or (3) for 20 or 48 h. All conditions were prepared in duplicate. Amino acids were derivatized to MOC esters, extracted, and analyzed via GC/P/IRMS using methods described in Sections 2.2, 2.3.

Additionally, to test for hydrogen isotope exchange with aqueous medium during hydrolysis and derivatization (Hill and Leach, 1964; Fogel et al., 2016; Silverman et al., 2022), BSA and a mixture of pure amino acid standards were separately hydrolyzed (6N HCl, 24 h, 110°C, oxic) and derivatized (0.1N HCl, 7:2:3 v/v/v anhydrous MeOH, pyridine, MCF) in aqueous solvent with different hydrogen isotope compositions.

### 2.5 Correction for derivative and exchangeable amine hydrogen

To determine the δ^2^H value of MeOH, 100 μg of disodium phthalate with known isotopic composition (Sessions et al., 2002) was derivatized in 2 ml of 20:1 (v/v) anhydrous MeOH:acetyl chloride (70°C, 30 min; Figure 2B). The derivatized product was extracted with 4 ml of 1:1 water:hexane, then measured by GC/P/IRMS, and contribution of disodium phthalate hydrogen was subtracted by mass balance.

The combined δ^2^H value of MCF and the remaining exchangeable amine hydrogen was characterized via derivatization of *p*-phenylenediamine (PPD)—a compound with two primary amine groups—with MCF and separate oxidation of PPD to dinitrobenzene (DNB), which has no nitrogen-bound hydrogen. One hundred mM of PPD was dissolved in 5 ml of anoxic dichloromethane with 3 eq. triethylamine (pre-distilled with CaH_2_ to remove any HCl generated in the reaction) and 0.1 eq. 4-dimethylamino pyridine, and was subsequently derivatized via an overnight reaction with 2.5 eq. MCF to yield dimethyl 1,4-phenylenedicarbamate (hereafter, “dicarbamate product”, or DCP in Equation 2; Figure 2C). The DCP was purified via flash column chromatography with silica gel. Five mg of DCP was dissolved in 2 ml of anhydrous MeOH, then 2 ml of distilled, deionized water was slowly added. The solution was mixed on a shaker for 2 h to ensure complete equilibration of the two amine hydrogen atoms with water, then the DCP was extracted once with 4 ml MTBE, filtered through a sodium sulfate column, and analyzed by GC/P/IRMS. In a separate reaction, PPD was oxidized to DNB using 8 eq. of *m*-chloroperbenzoic acid added under refluxing 1,2-dichloroethane in a procedure adapted from Liu et al. (2014) (Figure 2C). DNB was purified via flash column chromatography with silica gel, then 3 mg of DNB was dissolved in MTBE and analyzed via GC/P/IRMS. The δ^2^H value of MCF and the exchangeable nitrogen-bound hydrogen (MCF+NH) was obtained by solving for *F*_MCF+NH_ in the mass balance equation


(2) $$\begin{array}{l} 12F_{\text{DCP}}=8F_{\text{MCF}+\text{NH}}+4F_{\text{DNB}} \end{array}$$

where *F* is the fractional abundance (i.e., mole fraction) of ^2^H in each compound. PPD was chosen for these reactions (1) because of the favorable 8/4 ratio of (MCF + amine)/aromatic hydrogen in the dicarbamate product, and (2) because the exchangeable amine hydrogen atoms on the dicarbamate product and on MOC esters should have similar hydrogen isotope compositions when equilibrated in the same water supply, as the amine hydrogen in the dicarbamate product and in MOC esters share similar intramolecular bonding environments so should be controlled by similar equilibrium ^2^H/^1^H fractionation factors at a constant temperature (Wang et al., 2009).

## 3 Results

### 3.1 Derivative correction

Hydrogen atoms on amine and carboxyl groups rapidly exchange with water so do not contribute information about native δ^2^H_AA_ values. Derivatization of the carboxyl group removes the exchangeable hydrogen, but derivatization of the amine group removes only one of the two exchangeable hydrogen atoms (Figure 2A). In order to determine the isotope compositions of the native, non-exchangeable (i.e., carbon-bound) hydrogen on the amino acids, it is necessary to correct δ^2^H_AA_ values not only for the added derivative (MeOH and MCF) hydrogen, but also for the exchangeable amine hydrogen atom remaining after derivatization. A suitable method for this latter correction has eluded prior studies of δ^2^H_AA_ thus far, yet is imperative, as errors in reported δ^2^H_AA_ values can be on the order of 10 to 100‰ when the amine-bound hydrogen is improperly accounted for (Supplementary Figure S7; Supplementary Section 4). Here we developed a method to characterize the combined δ^2^H values of MCF and the exchangeable amine hydrogen by derivatizing PPD with MCF, separately oxidizing PPD to DNB (which removes all four of the nitrogen-bound hydrogens; Figure 2C), and analyzing both resulting products using GC/P/IRMS. Importantly, this approach requires derivatizing samples with the same reagents and water as those used to measure PPD.

**Figure 3:**
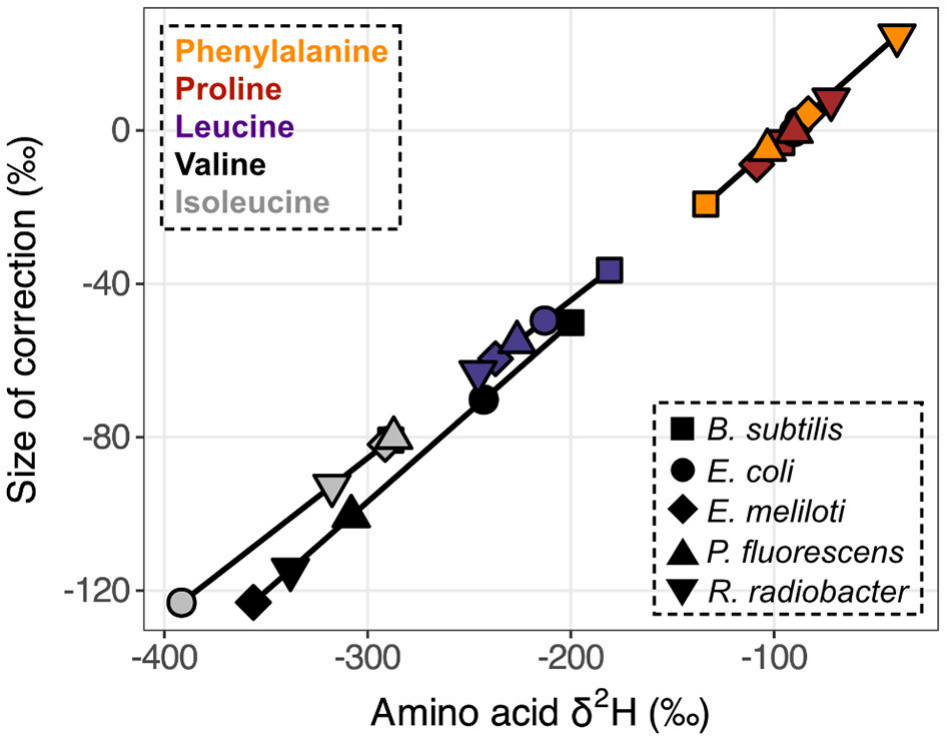
Size of derivative δ^2^H corrections vs. native amino acid isotopic compositions. Measured δ^2^H_AA_ values were corrected for derivative and exchangeable amine hydrogen contributions (δ^2^H_MeOH_ = –67.27 ± 1.78‰, δ^2^H_MCF+NH_ = –111.43 ± 1.96‰). Data displayed are from one replicate of wildtype organisms grown on glucose (shapes denote organisms as defined in the **lower right legend**). Amino acids are denoted by colors **(upper left legend)** and corresponding symbols are connected to highlight compound-specific magnitudes of correction effects. Horizontal error bars (indicating the propagated uncertainties (±1σ) from the amino acid and derivative measurements) are smaller than symbols.

The δ^2^H value of the MCF + amine hydrogen was –111.43 ± 1.96‰ when equilibrated with water of δ^2^H = –86.50 ± 0.36‰. The δ^2^H value of MeOH was –67.27 ± 1.78‰. Correction for these non-biological contributions generally shifted δ^2^H_AA_ values lower (Figure 3). For the relatively ^2^H-depleted amino acids (leucine, valine, and isoleucine), changes in δ^2^H values were substantial, reaching up to 123‰ for wildtype organisms grown on glucose. For the relatively ^2^H-enriched amino acids (proline and phenylalanine), this correction resulted in small or negligible changes, but in some cases resulted in higher δ^2^H values. However, note that correction of proline δ^2^H values using this approach may introduce small (<20‰) errors, as proline does not contain amine-bound hydrogen after derivatization, but the isotopic composition of MCF cannot be isolated from our measured δ^2^H value for MCF + amine hydrogen (see Supplementary Section 4). For other researchers to adopt our correction method, they will need to re-analyze PPD of known δ^2^H (available by request) with their own reagents and water.

### 3.2 Tests for isotopic alteration during sample preparation

Certain preparatory steps can alter the isotopic compositions of amino acids (reviewed in Silverman et al., 2022). In particular, degradation or non-quantitative recovery of amino acids during acid hydrolysis can lead to isotopic fractionation (e.g., Bada et al., 1989; Phillips et al., 2021). To assess the isotopic consequences of different hydrolysis conditions on δ^2^H_AA_ values, standard BSA protein was hydrolyzed at different temperatures (105 or 110°C), for different durations (20, 24, or 48 h), anoxically or with O_2_ present. Compared to conventional hydrolysis conditions (6N HCl, 110°C, 20-24 h, anoxic; Silverman et al., 2022), no treatment significantly altered the hydrogen isotope composition of amino acids (Supplementary Table S4).

To investigate whether carbon-bound hydrogen in amino acids exchanges with aqueous medium during hydrolysis or derivatization, BSA and a mixture of amino acid standards were separately hydrolyzed (6N HCl, 110°C, 24 h) and derivatized to MOC esters in solvents with different isotopic compositions. Slopes of regressions of amino acid vs. water δ^2^H values (Supplementary Figures S9, S10) represent the equilibrium fractionation factor (α_eq_) between organic hydrogen and water, multiplied by the fraction of hydrogen exchanged in the amino acid. To estimate the maximum percent of carbon-bound hydrogen exchanged, each slope was divided by α_eq_ = 0.9, an estimate based on fractionation factors for a variety of hydrogen positions in linear and cyclic organic molecules (Wang et al., 2009, 2013). These calculations indicate that ten amino acids experienced negligible (<2%) hydrogen exchange with aqueous medium during hydrolysis, while tryptophan experienced significant exchange (~27%; Supplementary Figure S9). Asparagine + aspartic acid (Asx), glutamine + glutamic acid (Glx), and tyrosine experienced moderate exchange (4%–10%); this effect has been previously demonstrated through deuterated and tritiated hydrolysis experiments (Hill and Leach, 1964; Fogel et al., 2016) and is likely due to the increased lability of hydrogen adjacent to the polar—R groups. Hydrogen exchange in tryptophan may have occurred through a reversible reaction with sulfur-containing amino acids in the presence of oxygen (common tryptophan degradation mechanisms summarized in Silverman et al., 2022). All amino acids experienced low (<2%) hydrogen exchange during derivatization (Supplementary Figure S10).

### 3.3 ^2^H/^1^H fractionations and carbon fluxes across wildtype organisms grown on glucose

**Figure 4:**
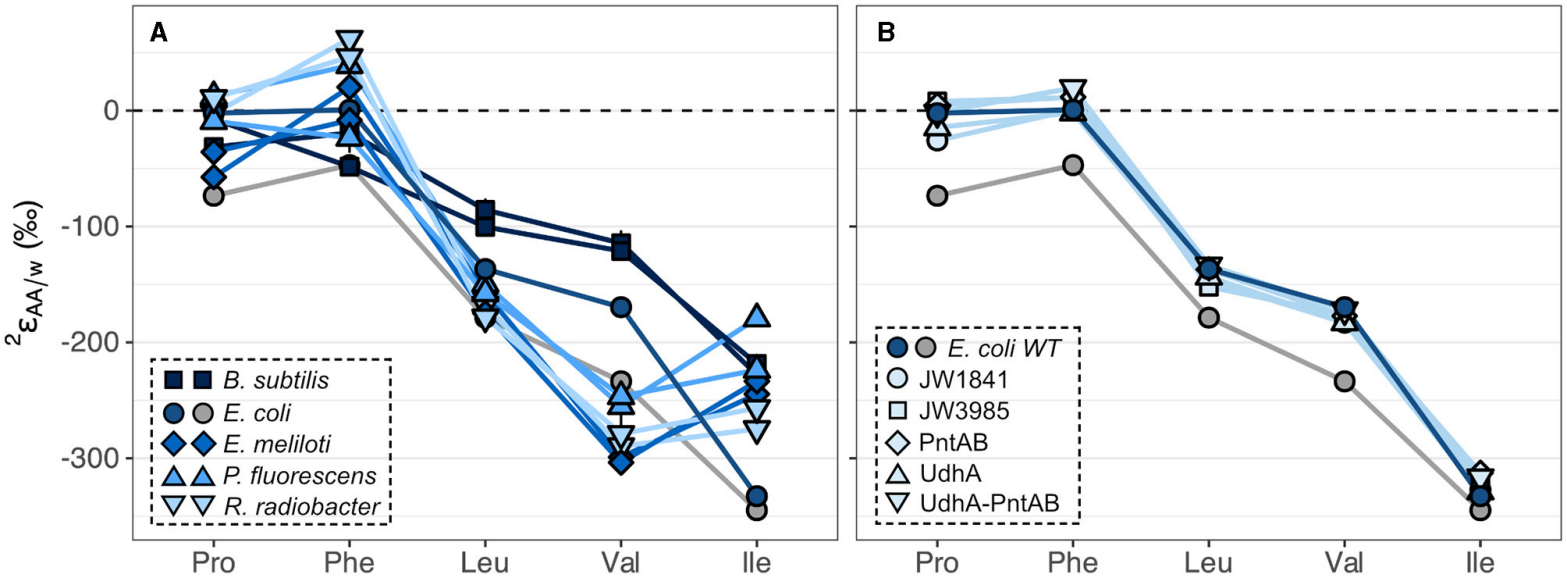
Summary of ^2^H/^1^H fractionations between amino acids and water in wildtype organisms **(A)** and in *E. coli* wildtype (WT) and mutant organisms **(B)** grown on glucose. Wildtype cultures were grown in biological duplicate, except for *E. coli*, which was grown in two non-replicate cultures (see Sections 2.1, 2.2) distinguished by blue and gray symbols for cultures #1 and #2, respectively. Error bars indicate the propagated uncertainties (±1σ) from the amino acid, derivative, and water measurements and are smaller than symbols. Amino acids are proline (Pro), phenylalanine (Phe), leucine (Leu), valine (Val), and isoleucine (Ile).

The substantial variations in ${\;}^{2}\varepsilon_{\text{AA/w}}$ values within wildtype organisms grown on glucose are summarized in Figure 4A and Supplementary Table S2 for the five amino acids analyzed, and in Supplementary Figure S6 and Supplementary Table S3 for the other amino acids measured in this study. All organisms produced similar ${\;}^{2}\varepsilon_{\text{AA/w}}$ patterns, where phenylalanine and proline were the most ^2^H-enriched, while isoleucine and valine were the most ^2^H-depleted. This pattern mirrors that previously observed for *E. coli* (Fogel et al., 2016), with the exception of phenylalanine, which in our study was significantly more ^2^H-enriched relative to the average. Within a single organism, the five ${\;}^{2}\varepsilon_{\text{AA/w}}$ values spanned large ranges (220‰–352‰). Valine exhibited the largest variation across organisms (190‰) while proline and leucine ${\;}^{2}\varepsilon_{\text{AA/w}}$ values varied the least (86‰–93‰). ${\;}^{2}\varepsilon_{\text{AA/w}}$ values from biological replicates of wildtype organisms grown on glucose were generally reproducible (within 30‰) except for phenylalanine and isoleucine from *P. fluorescens* cultures (Supplementary Figure S5; Supplementary Table S2), which differed between replicates for unknown reasons. ${\;}^{2}\varepsilon_{\text{AA/w}}$ values in *E. coli* cultures grown on glucose also differed by >30‰ for four of the amino acids, but these cultures are not considered biological replicates due to differences in sample preparation (see Sections 2.1, 2.2).

**Figure 5:**
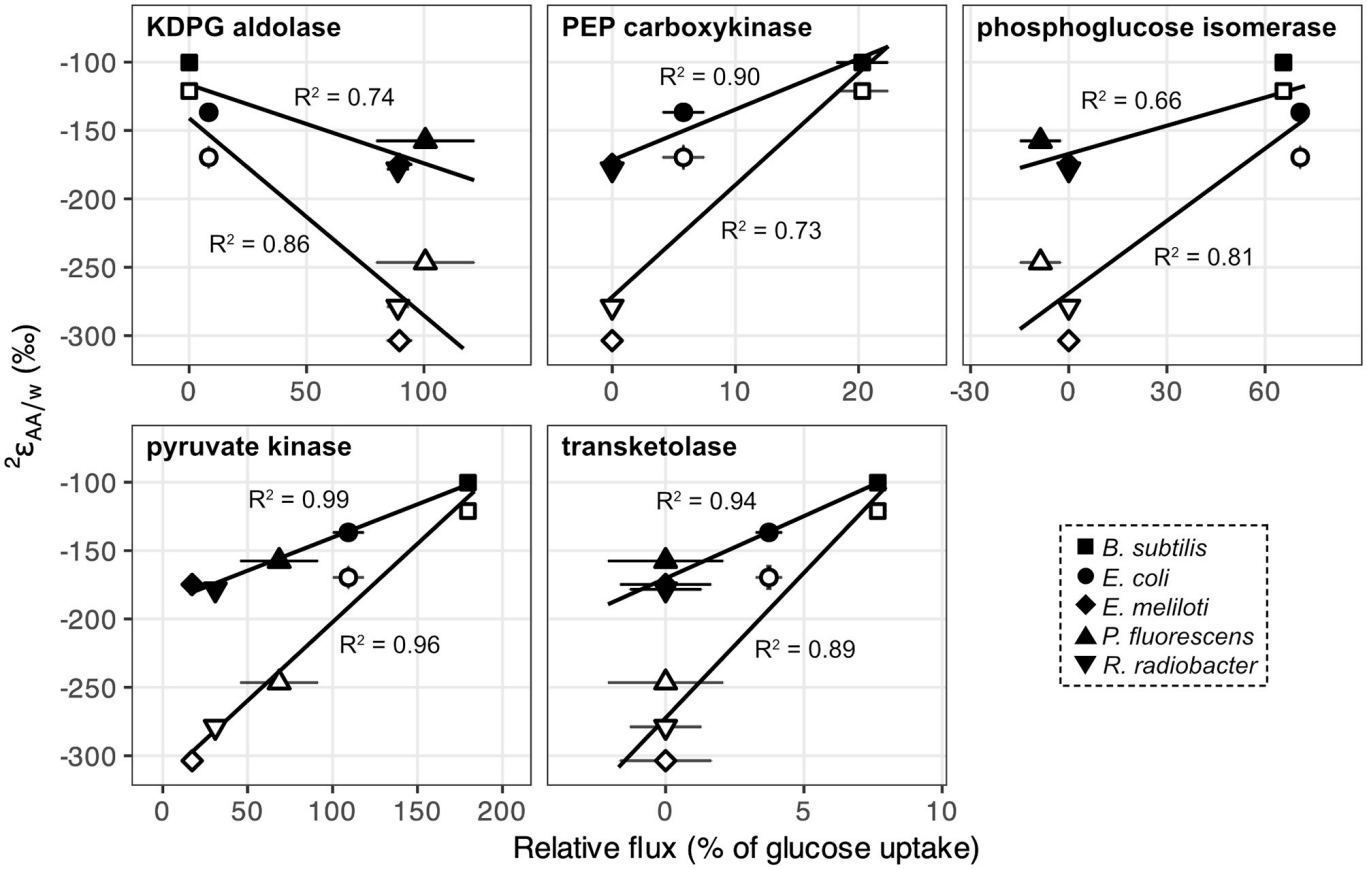
${\;}^{2}\varepsilon_{\text{AA/w}}$ values for leucine (black) and valine (white) in wildtype organisms grown on glucose vs. relative carbon flux (i.e., normalized to glucose uptake rates) through pyruvate synthesis-related enzymes in central metabolism: KDPG aldolase (ED pathway), PEP carboxykinase (anaplerotic pathway), phosphoglucose isomerase (EMP pathway), pyruvate kinase (EMP + ED pathways), and fructose 6-phosphate-forming transketolase (PP pathway). Error bars represent ±1σ. Regression analyses were performed using ${\;}^{2}\varepsilon_{\text{AA/w}}$ values from the first of each biological replicate condition (see Supplementary Table S2).

Metabolic flux analysis carried out in a previous study (Wijker et al., 2019) revealed substantial differences in pathways used for glucose breakdown and in carbon fluxes through central metabolic enzymes (Supplementary Figure S3; Supplementary Table S1; Wijker et al., 2019). *E. coli* and *B. subtilis* primarily used the EMP pathway for glucose catabolism and excreted high fluxes of acetate. In contrast, *E. meliloti* and *R. radiobacter* mainly relied on the ED pathway to metabolize glucose and exhibited moderate fluxes through the TCA cycle. *P. fluorescens* exhibited high fluxes through the ED pathway and TCA cycle, as well as periplasmic conversion of glucose to gluconate and 2-ketogluconate, and cyclic flux through the EDEMP pathway (Nikel et al., 2015; Wijker et al., 2019). ${\;}^{2}\varepsilon_{\text{AA/w}}$ values for leucine and valine correlated with carbon fluxes through enzymes related to pyruvate synthesis: KDPG aldolase (ED pathway), PEP carboxykinase (anaplerotic pathway), phosphoglucose isomerase (EMP pathway), pyruvate kinase (EMP + ED pathways), and transketolase (PP pathway; Figure 5). Directions of correlations between ${\;}^{2}\varepsilon_{\text{AA/w}}$ values and carbon flux through EMP and ED pathways opposed those observed for lipid/water fractionations (${\;}^{2}\varepsilon_{\text{L/w}}$, presented in Wijker et al., 2019), although these relationships may not be directly comparable, as ${\;}^{2}\varepsilon_{\text{L/w}}$ values are primarily controlled by NADPH metabolism (Zhang et al., 2009; Wijker et al., 2019) while leucine and valine do not inherit hydrogen from NADPH. ${\;}^{2}\varepsilon_{\text{AA/w}}$ values for other amino acids did not correlate with carbon flux through any central metabolic enzyme in wildtype organisms.

### 3.4 ^2^H/^1^H fractionations and carbon fluxes in *E. coli* knockout mutants grown on glucose

Specific dehydrogenase and transhydrogenase genes were deleted in *E. coli* organisms in a previous study (Wijker et al., 2019) to interrogate the influence of NADPH on lipid δ^2^H values. Deletion of these genes forced carbon flux through alternative central metabolic enzymes to accomplish glucose catabolism and NADPH balance (Supplementary Figure S4; Table S1). Despite drastic differences in the magnitudes of carbon fluxes, *E. coli* mutant organisms produced similar ${\;}^{2}\varepsilon_{\text{AA/w}}$ values compared to wildtype *E. coli* culture #1, differing by <40‰ for any given amino acid (Figure 4B; Supplementary Table S2; see note about *E. coli* culture #2 in Section 3.3). Lipid δ^2^H values showed a similar response in these organisms (Wijker et al., 2019), as did δ^2^H_AA_ values in *E. coli* mutants with inhibited glycolysis or oxidative pentose phosphate pathways in a previous study (Smith et al., 2022). Nevertheless, variations in isotopic compositions hint at some control by NADPH, as δ^2^H_AA_ values correlated with carbon fluxes through all NADPH-related enzymes (Supplementary Figure S15), with proline exhibiting the strongest correlations (R^2^ = 0.70–0.86), followed by phenylalanine (R^2^ = 0.58–0.71), then isoleucine (R^2^ = 0.36–0.49). The PGI knockout mutant, JW3985, had a severely perturbed metabolism and fell off the regressions in most cases so was excluded from the regression analyses.

### 3.5 ^2^H/^1^H fractionations across wildtype organisms grown on different substrates

**Figure 6:**
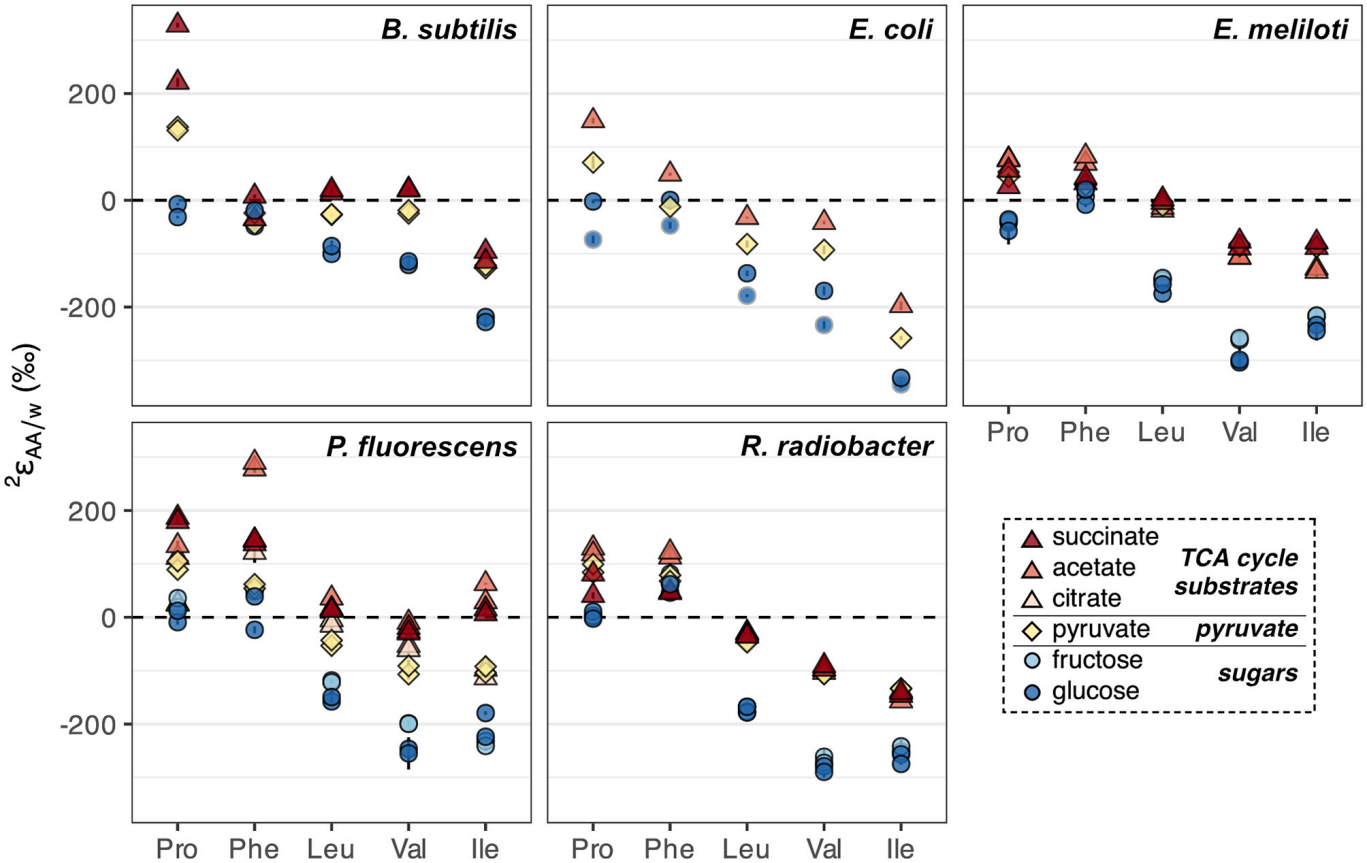
Summary of ^2^H/^1^H fractionations between amino acids and water in wildtype organisms grown on different substrates (denoted by colors) spanning metabolically distinct classes (denoted by shapes). Error bars indicate the propagated uncertainties (±1σ) from the amino acid, derivative, and water measurements, and are smaller than symbols. Duplicate cultures were set up for all organisms and conditions except *E. coli*, which was grown on acetate and pyruvate in a single replicate, and on glucose in two different (non-replicate) experiments (with cultures #1 and #2 distinguished by dark blue circles with black and gray borders, respectively; see Sections 2.1, 2.2).

In addition to glucose, wildtype organisms were cultured on acetate, citrate, fructose, pyruvate, and/or succinate, which enter central metabolism at different nodes and activate different catabolic pathways for substrate breakdown. ${\;}^{2}\varepsilon_{\text{AA/w}}$ values from biological replicates of wildtype organisms grown on each substrate were generally reproducible (within 30‰) except for proline and phenylalanine in *B. subtilis* grown on succinate, and proline in *R. radiobacter* grown on succinate, which differed between replicates for unclear reasons (Figure 6, Supplementary Figure S5, Supplementary Table S2). Fructose led to similar ${\;}^{2}\varepsilon_{\text{AA/w}}$ values as glucose-based growth, while pyruvate and TCA cycle substrates (acetate, citrate, and succinate) led to ^2^H-enrichment of all amino acids (Figure 6), mirroring phenomena observed for lipids (Zhang et al., 2009; Osburn et al., 2016; Wijker et al., 2019). The amount of ^2^H-enrichment varied widely for each amino acid. Proline in *B. subtilis* grown on succinate, and isoleucine and phenylalanine in *P. fluorescens* grown on acetate, exhibited the largest singular ^2^H-enrichments (286‰–360‰ higher ${\;}^{2}\varepsilon_{\text{AA/w}}$ values relative to those during glucose-based growth). However, phenylalanine was generally ^2^H-enriched by the least amount (<100‰ difference between ${\;}^{2}\varepsilon_{\text{Phe/w}}$ values upon growth on TCA cycle substrates relative to on glucose for all organisms except *P. fluorescens*), while valine was generally ^2^H-enriched by the greatest amount (142–245‰). These differences were significantly greater than those between growth water (<15‰) or substrate δ^2^H values (<85‰; Supplementary Table S2).

## 4 Discussion

Consistencies in ${\;}^{2}\varepsilon_{\text{AA/w}}$ patterns (i.e., the relative ordering of ${\;}^{2}\varepsilon_{\text{AA/w}}$ values) within each growth condition, coupled with the substantial shifts in ${\;}^{2}\varepsilon_{\text{AA/w}}$ values across growth conditions, underscore the existence of systematic controls on δ^2^H_AA_ values. Initial investigations (Fogel et al., 2016; Newsome et al., 2020; Gharibi et al., 2022a; Smith et al., 2022; Mancuso et al., 2023) have begun to explore the complicated factors driving δ^2^H_AA_ signals in heterotrophic microbes, mammals, and humans, but we are still far from mechanistic understanding of these controls. In the following sections we interrogate several biochemical controls on the patterns and variations in microbial δ^2^H_AA_ values in an attempt to elucidate how these signals can be used as tracers for microbial or ecological studies in the environment (summarized in Table 1). Our data allow us to provide a mechanistic explanation for some, though not all, of the observed ${\;}^{2}\varepsilon_{\text{AA/w}}$ patterns. As numerous enzymes in central metabolism are referenced throughout the discussion, a schematic of the central metabolic pathways with all enzymes annotated is provided for reference (Supplementary Figure S2).

**Table 1:** Summary of potential biochemical controls on δ^2^H_AA_ values.

**AA(s)**	**Biochemical control hypothesized**	**Effect on δ^2^H_AA_ values**	**Data where effect is observed/explored**	**Potential application of δ^2^H_AA_ analysis**
Pro	Citrate synthase: KIE leads to ^2^H-enrichment of α-ketoglutarate (proline precursor in TCA cycle).	Stimulates high proline δ^2^H values, i.e., small proline/water fractionations.	High proline δ^2^H values across all carbon substrate conditions (Figure 6). Large slopes in regressions of proline vs. water δ^2^H (growth water experiments; Supplementary Figure S11), implying small proline/water fractionations (Supplementary Section 6.1).	
Phe	High fraction of water-derived hydrogen in precursors PEP and erythrose-4-phosphate, with water-derived hydrogen having equilibrated with water.	Leads to small but positive phenylalanine/water fractionations, with phenylalanine δ^2^H values relatively insensitive to diet.	Supported by small phenylalanine/water fractionations across all substrate conditions (except in *P. fluorescens* grown on TCA cycle substrates; Figure 6).	Proxy for environmental water δ^2^H. Bio-thermometer if organic hydrogen/water equilibration is temperature-dependent.
Pro, Phe, Ile	NADPH metabolism: KIEs of dehydrogenases and transhydrogenases control the δ^2^H value of the NADPH pool.	Contributes to some variations in δ^2^H values of amino acids with NADPH-derived hydrogen.	Correlations between δ^2^H_AA_ values and carbon flux through NADPH-related enzymes, and between δ^2^H_AA_ values and NADPH imbalance fluxes in *E. coli* organisms (Supplementary Figure S15; Section 4.2.1.2). Correlations between δ^2^H_AA_ shifts in organisms grown on glucose → TCA cycle substrates and fraction of NADPH-derived amino acid hydrogen (Figure 7B, Supplementary Figure S13). Substrate ordering of estimated NADPH-related ^2^H- enrichment of proline with measured and published NADPH imbalance fluxes in *E. coli* and *B. subtilis* (Figure 7C; Section 4.2.1.2).	Elucidate NADPH balance and redox metabolism in cells.
All AAs	Catabolic pathways activated: control δ^2^H values of central metabolites (e.g., pyruvate) through differential activation of catabolic pathways and associated enzymes.	Contributes to systematic variations in δ^2^H_AA_ values, with lowest δ^2^H values upon growth on sugars, moderate upon growth on pyruvate, and highest upon growth on TCA cycle substrates.	Correlations between leucine and valine δ^2^H values and carbon flux through pyruvate synthesis-related enzymes in organisms grown on glucose (Figure 5). Correlations between δ^2^H_AA_ shifts in organisms grown on glucose → TCA cycle substrates and fraction of pyruvate-derived amino acid hydrogen (Figure 7B, Supplementary Figure S12). Systematic variations in δ^2^H_AA_ values (Figure 7A) explainable by considering relative ^2^H-enrichment of cellular pyruvate in different carbon substrate conditions (Figure 8; Section 4.2.1.1).	Interrogate an organism's diet and/or metabolic lifestyle.
	Enzymes in biosynthetic pathways.	Set the overall pattern of δ^2^H_AA_ values, but variations in enzymes and isotope effects across organisms may contribute to variations in δ^2^H_AA_ values.	Similar δ^2^H_AA_ patterns across organisms and substrate conditions (Figure 6). Similar δ^2^H_AA_ values across *E. coli* organisms grown on glucose, despite different fluxes through catabolic pathways (Figure 4B). Diversity in isozymes employed in each amino acid biosynthetic step across organisms (Supplementary Figure S20).	Fingerprinting method to trace origins of amino acids in organic matter, if δ^2^H_AA_ patterns within different taxonomic groups are unique.

### 4.1 Controls on the ${\;}^{2}\varepsilon_{\text{AA/w}}$ pattern during glucose metabolism

${\;}^{2}\varepsilon_{\text{AA/w}}$ patterns were strikingly similar across wildtype organisms and *E. coli* mutants grown on all carbon substrates (Figures 4, 6), indicating that biosynthetic pathways (as opposed to catabolic pathways) serve as first-order controls on δ^2^H_AA_ values. Our interpretation is consistent with Fogel et al. (2016), who observed similar δ^2^H_AA_ patterns in *E. coli* cultured on glucose or tryptone in different growth waters. In this section, we investigate the biochemical factors that set the general ${\;}^{2}\varepsilon_{\text{AA/w}}$ pattern in glucose-grown cultures, focusing our interpretation on hydrogen sources and mechanisms of hydrogen exchange, as well as relevant isotope effects associated with enzymes in central metabolic and biosynthetic pathways. Based on these interpretations, we speculate on the most prominent types of biological information that can be obtained from δ^2^H_AA_ measurements. Amino acids are discussed in order from most to least ^2^H-enriched. As these microbes share the same biosynthetic pathways, variations in ^2^ε values of a given amino acid across organisms (e.g., Figure 4A) hint at the importance of additional, second-order controls, which are examined in Section 4.2.

#### 4.1.1 Proline

The high δ^2^H values of proline are likely due in large part to the ^2^H-enriching kinetic isotope effect (KIE) of citrate synthase in the TCA cycle. Proline is mainly synthesized from the TCA cycle intermediate α-ketoglutarate and inherits four hydrogen atoms from α-ketoglutarate, two from NAD(P)H, and one from water (Figure 1, Supplementary Figure S16). In the first step of the TCA cycle, citrate synthase combines acetyl-CoA with oxaloacetate to form citrate, abstracting a proton from acetyl-CoA's methyl group with a large KIE (1.94 measured *in vitro*; Lenz et al., 1971). The resulting ^2^H-enriched hydrogen in citrate is retained through formation of α-ketoglutarate (and ultimately, synthesis of proline), as aconitase and isocitrate dehydrogenase stereospecifically remove the oxaloacetate-derived hydrogen from citrate and isocitrate, respectively (Supplementary Figure S16; Lowenstein, 1967; Smith and York, 1970; Csonka and Fraenkel, 1977; Ochs and Talele, 2020). As proline inherits ~30% of its hydrogen from NAD(P)H through this synthesis pathway, and its δ^2^H value appears to be controlled to some extent by NADPH metabolism (see Section 4.2.1.2), proline may be a sensitive indicator of redox balance in cells. Some microbial species within the families Rhizobiaceae and Pseudomonadaceae can additionally synthesize proline from ornithine, which in turn is synthesized from arginine (Stalon et al., 1987; Schindler et al., 1989). The prevalence of this pathway in the *P. fluorescens* and *R. radiobacter* strains examined in this study is unclear, but its operation would presumably reduce the fraction of NAD(P)H-derived hydrogen in proline.

#### 4.1.2 Phenylalanine

The source of phenylalanine's high δ^2^H values is unclear, but may be due to relatively large fractions of water-derived hydrogen in phenylalanine's organic precursors (phosphoenolpyruvate and erythrose-4-phosphate; Figure 1, Supplementary Figure S17). During glucose metabolism, phosphoenolpyruvate (PEP) is primarily synthesized through the EMP or ED pathway, and its hydrogen can be directly routed from glucose or partially exchanged with water (e.g., at the triose phosphate level; Rose and O'Connell, 1961; Saur et al., 1968; Reynolds et al., 1971; Russell and Young, 1990). Erythrose-4-phosphate is synthesized through the PP pathway, which includes numerous isomerizations and reversible reactions that exchange organic hydrogen with water (Russell and Young, 1990). As these equilibrations are presumably controlled by equilibrium rather than kinetic isotope effects, the resulting fractionations are unlikely to be strongly negative (in contrast to the potentially large normal KIEs expressed during water incorporation into pyruvate; Section 4.1.3). Indeed, theoretical calculations predict slightly negative to relatively positive equilibrium fractionations for the hydrogen sites susceptible to equilibration with water in the organic intermediates (Wang et al., 2009). The relatively small isotopic fractionation of phenylalanine across carbon substrate conditions (Figure 6) further supports a large fraction of water-derived hydrogen, which may render phenylalanine a useful proxy for environmental water δ^2^H, and potentially a bio-thermometer if equilibration with water is temperature-dependent.

#### 4.1.3 Leucine and valine

The low δ^2^H values of leucine and valine, as well as the consistent ^2^H-depletion of valine relative to leucine in glucose-grown organisms, may be attributed in part to low pyruvate δ^2^H values. Leucine and valine are formed through overlapping biosynthetic pathways, initiated by condensation of two pyruvate molecules to form 2-acetolactate. Both pyruvate methyl groups remain intact through this and subsequent steps, ultimately becoming part of the isopropyl groups of these amino acids (Figure 1, Supplementary Figure S18). During glucose metabolism, pyruvate's methyl hydrogen is likely to become ^2^H-depleted relative to the methylene hydrogen in its central metabolite precursors (PEP, KDPG, and malate), as all pyruvate synthesis reactions incorporate solvent hydrogen with potentially large isotope effects, ranging from –141‰ for equilibrium-controlled incorporation (Wang et al., 2009) to presumably larger fractionations for kinetically-controlled transfers. For example, solvent isotope effects measured *in vitro* for pyruvate kinase and KDPG aldolase were 1,700 and 3,250‰, respectively (Meloche, 1975; Bollenbach et al., 1999), although note that the applicability of such measurements to *in vivo* studies is untested, and enzyme reversibility (such as with KDPG aldolase; Jacobson et al., 2019) as well as keto-enol tautomerization of pyruvate (Chiang et al., 1992) would drive pyruvate δ^2^H values toward equilibrium. As valine inherits a larger proportion of pyruvate hydrogen (Figure 1, Supplementary Figure S18), variations in the isotopic composition of pyruvate would result in more pronounced changes in the δ^2^H value of valine compared to leucine, as observed here (Figure 5). All additional carbon-bound hydrogen in leucine and valine are likely transferred from water, and the isotope compositions of these hydrogen atoms may reflect equilibrium and/or kinetic control. Pyruvate occupies a crucial node at the intersection of many branches of central metabolism. The isotope composition of its methyl hydrogen should therefore be sensitive to the central metabolic pathways activated (see Section 4.2.1.1), and consequently to the types of carbon substrates consumed by an organism. As leucine and valine inherit unaltered pyruvate methyl hydrogen (in addition to isotopically invariant water hydrogen), these amino acids should also be sensitive to organisms' diet if they are synthesized de novo.

#### 4.1.4 Isoleucine

As with leucine and valine, low isoleucine δ^2^H values in organisms grown on glucose may be the result of relatively ^2^H-depleted hydrogen sources (pyruvate and NADPH) and large normal KIEs associated with hydrogen transfer from water. Isoleucine is formed from oxaloacetate, which in turn is synthesized from malate in the TCA cycle, or from PEP or pyruvate through anaplerotic pathways (Figure 1, Supplementary Figure S2, S19). The *pro-S* position of oxaloacetate's methylene group is retained through biosynthesis of isoleucine (Supplementary Figure S19); depending on the extent to which oxaloacetate undergoes keto-enol tautomerization (i.e., intercoverts between a ketone and enol(ate) structure; Kosicki, 1962; Bruice and Bruice, 1978) and/or is synthesized by malate dehydrogenase (thereby incorporating solvent hydrogen into oxaloacetate's *pro-S* methylene position; Gawron and Fondy, 1959; Omi et al., 2003), the hydrogen atom retained may originate from water (see details in Supplementary Figure S19 caption). Together, this hydrogen, along with the water-derived fraction in pyruvate's methyl group (Section 4.1.3) and those transferred from or equilibrated with water during isoleucine biosynthesis, contribute to a potentially large (≥60%) fraction of carbon-bound hydrogen in isoleucine sourced from water (Supplementary Figure S19)—an estimate consistent with model predictions by Fogel et al. (2016). In contrast to phenylalanine, whose water-derived hydrogen may be predominantly acquired through equilibrium exchange reactions, isoleucine's water-derived hydrogen may be primarily transferred from water by enzymatic reactions with large KIEs, contributing to very low isoleucine δ^2^H values. Despite its high water fraction, the δ^2^H value of isoleucine remains sensitive to the metabolic programming of cells (i.e., both redox balance and central metabolic pathways activated) through its pyruvate and NADPH hydrogen, as evident through the large δ^2^H variations across growth conditions (Figure 6). This sensitivity may be enhanced by the different origins of oxaloacetate-sourced hydrogen in isoleucine (water vs. organic hydrogen), which vary depending on which carbon substrates are being catabolized.

### 4.2 Controls on variations in ${\;}^{2}\varepsilon_{\text{AA/w}}$ values

While the similar patterns of ${\;}^{2}\varepsilon_{\text{AA/w}}$ values across glucose-grown organisms reveals remarkable consistencies in net isotopic fractionations of central metabolic and biosynthetic pathways, the variations in ${\;}^{2}\varepsilon_{\text{AA/w}}$ values hint at subtle physiological differences in the organisms. Culturing experiments in which the δ^2^H value of an organism's growth water is manipulated can provide some constraints on the biochemical causes of these differences (presented in Supplementary Section 6.1). The substantial systematic shifts in ${\;}^{2}\varepsilon_{\text{AA/w}}$ values upon growth on different carbon substrates provide further insight into biochemical controls on these isotopic signals, including the differential activation of enzymes across catabolic pathways (Section 4.2.1). Finally, potential differences in ${\;}^{2}\varepsilon_{\text{AA/w}}$ values due to enzymatic variations in biosynthetic pathways are considered (Section 4.2.2).

#### 4.2.1 Metabolite pools in activated catabolic pathways

**Figure 7:**
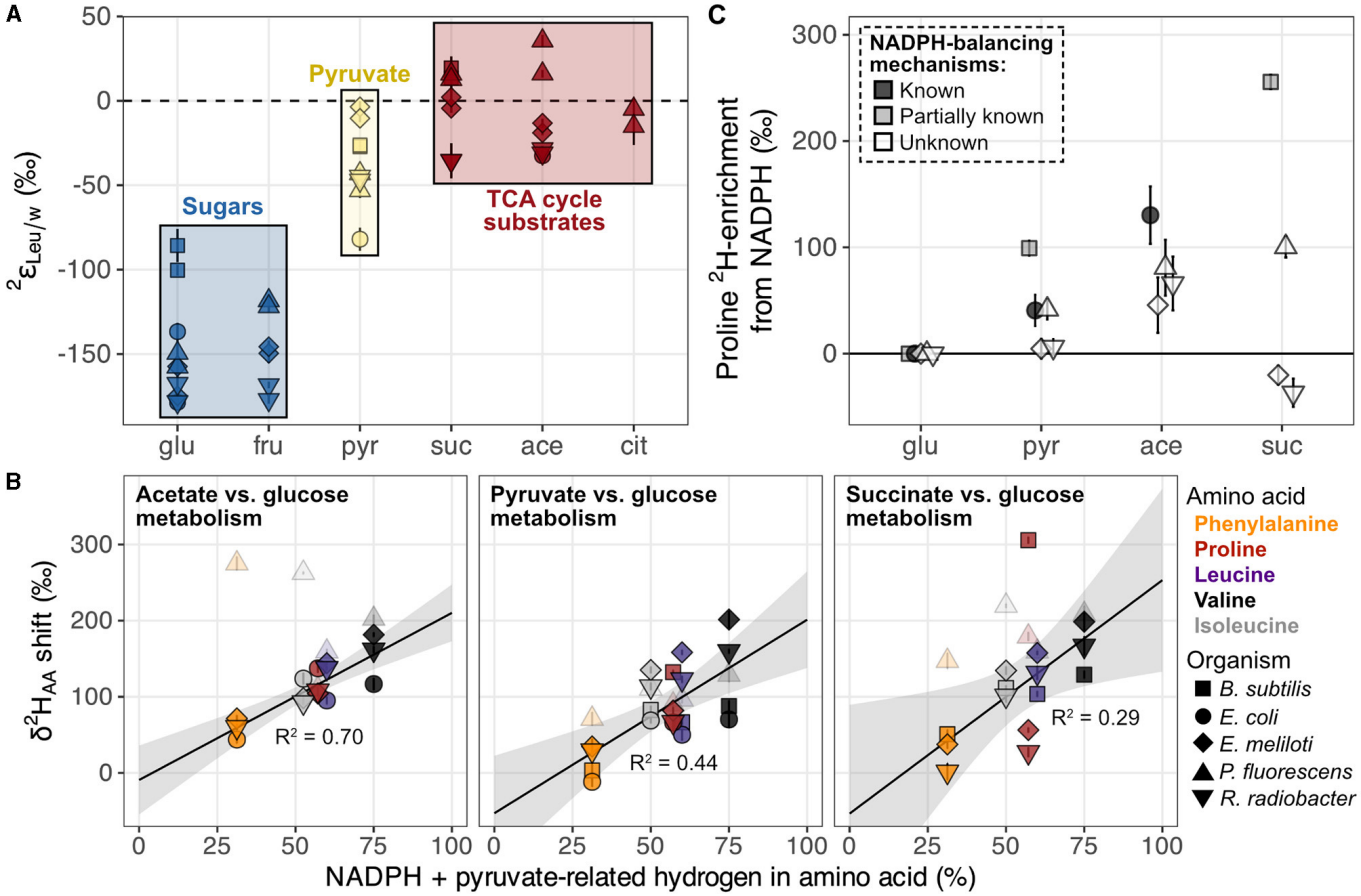
Hypotheses explored for pyruvate and NADPH controls on δ^2^H_AA_ variations in organisms grown on different substrates. **(A)** Example of systematic shifts in ${\;}^{2}\varepsilon_{\text{AA/w}}$ values for one amino acid (leucine) in wildtype organisms grown on metabolically distinct classes of carbon substrates (denoted by colors). All ${\;}^{2}\varepsilon_{\text{Leu/w}}$ values from Figure 6 are plotted. Error bars indicate the propagated uncertainties (±1σ) from the amino acid, derivative, and water measurements, and are smaller than symbols. **(B)** Estimated influence of NADPH- and pyruvate-derived hydrogen on shifts in δ^2^H_AA_ values across growth conditions. See Section 4.2.1 and Supplementary Sections 6.2, 6.3 for details on accounting estimates. δ^2^H_AA_ shifts were calculated as the difference in δ^2^H value of a given amino acid between the two substrate conditions compared within each panel (using data from replicate culture #1 for each condition). Error bars on individual data points are the propagated uncertainties (±1σ) from each pair of δ^2^H_AA_ values measured and are smaller than symbols. The shaded gray region indicates the 95% confidence interval of the coefficients from the linear regression fit; R^2^ values are adjusted for the number of predictors in the model. *P. fluorescens* data are shown as transparent symbols but excluded from the regressions due to multiple instances as extreme outliers. When *P. fluorescens* data are included in the regressions, adjusted R^2^ values are 0.02, 0.43, and 0.21 for shifts from glucose to acetate, pyruvate, and succinate metabolism, respectively. Colors denote amino acids as shown in the legend to the right of the plot. **(C)** NADPH-driven ^2^H-enrichment of proline (i.e., that beyond ^2^H-enrichment due to acetyl-CoA alone) estimated in wildtype organisms cultured on the indicated substrate compared to during growth on glucose (data from replicate #1 cultures used). See Section 4.2.1.2 and Supplementary Section 6.3 for calculation details. Error bars indicate combined uncertainties in measured proline δ^2^H values (Supplementary Table S2), estimated shifts in pyruvate δ^2^H values (Supplementary Table S6), and/or measured acetate and pyruvate δ^2^H values (reported in Zhang et al., 2009). Data are slightly spread about the *x*-axis to increase ease of visualization. In all three figures, shapes denote the organism as shown in the bottom right legend. Growth substrates are glu, glucose; fru, fructose; pyr, pyruvate; suc, succinate; ace, acetate; and cit, citrate.

**Figure 8:**
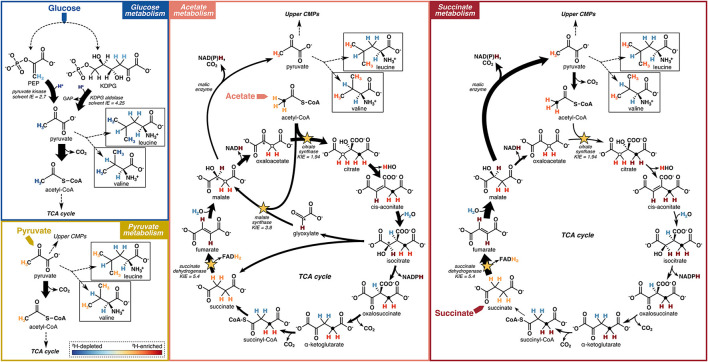
Schematic models of hydrogen flow through relevant central metabolic pathways (CMPs), corresponding isotopic fractionations, and relative ^2^H-enrichment of leucine and valine upon growth of microbes on different carbon substrates. Reactions potentially having a strong influence on the hydrogen isotope compositions of pyruvate and acetyl-CoA, and thus on the amino acids investigated in this study, are highlighted. Arrow widths indicate flux magnitudes estimated based on ^13^C-based metabolic flux maps reported in Gerosa et al. (2015). Dashed lines summarize a series of reactions not shown. Filled circles in the TCA cycle metabolites trace the methyl group of acetyl-CoA through succinyl-CoA. Open circles trace the methyl group of acetyl-CoA into malate and through citrate via the glyoxylate shunt (acetate metabolism) and the methylene group of oxaloacetate into citrate (succinate metabolism). The asterisk traces the alcohol carbon position in isocitrate to oxaloacetate via the glyoxylate shunt (acetate metabolism). The color gradient representing the relative ^2^H-enrichments or ^2^H-depletions of hydrogen within biomolecules is shown as an inset in the “Pyruvate metabolism” box. Isotope effects (KIEs and solvent IEs) shown are reported from Rétey et al. (1970), Lenz et al. (1971), Meloche (1975), O'Leary (1989), and Bollenbach et al. (1999).

Growth of organisms on carbon substrates that activated different catabolic pathways led to substantial shifts in δ^2^H_AA_ values (Figures 6, 7A). In general, growth on sugars (glucose and fructose) led to the most ^2^H-depleted amino acids, followed by growth on pyruvate, then TCA cycle substrates (acetate, citrate, and succinate). The overall pattern is similar to that observed in lipids (Zhang et al., 2009; Wijker et al., 2019). Moreover, patterns of ^2^H-enrichment relative to δ^2^H_AA_ values in glucose-grown cells were similar within pairs of metabolically-related organisms (*B. subtilis* + *E. coli* and *E. meliloti* + *R. radiobacter*), highlighting organismal physiology as an important control on δ^2^H_AA_ values. The altered metabolic programming in organisms grown on different substrates undoubtedly alters the hydrogen isotope composition of central metabolites that feed into amino acid biosynthesis. In particular, pyruvate occupies a crucial node in central metabolism and contributes hydrogen to all amino acids either directly (the case for leucine, valine, and isoleucine), or indirectly (for proline and phenylalanine; see Supplementary Section 6.2), so likely controls some of the variation in the δ^2^H_AA_ values. Furthermore, some amino acids inherit hydrogen from NADPH (29% in proline, 20% in isoleucine, and 13% in phenylalanine; Figure 1, Supplementary Figures S16–S19). NADPH serves as a hydride carrier in organisms, both providing reducing power for anabolic reactions and transmitting isotopic information to products. As lipid δ^2^H variations are thought to be primarily controlled by the hydrogen isotope composition of the NADPH pool (Zhang et al., 2009; Wijker et al., 2019), amino acid δ^2^H values may be similarly influenced by NADPH metabolism. Together, changes in the isotopic composition of the pyruvate-related and NADPH fractions of hydrogen in the amino acids explain 29%–70% of the shifts in δ^2^H_AA_ values between pairs of substrate conditions (e.g., when *E. coli* is grown on acetate vs. glucose; Figure 7B; Supplementary Sections 6.2, 6.3). Stated a different way, δ^2^H_AA_ values may vary with the inverse of the fraction of water-derived hydrogen in amino acids, however further work is required to quantify these fractions under different metabolic conditions. In the following two sections we separately explore the influences of pyruvate and NADPH on δ^2^H_AA_ variations, with mechanisms affecting pyruvate δ^2^H summarized in Figure 8.

##### 4.2.1.1 Pyruvate δ^2^H

The substrate-driven ordering of amino acid ^2^H-enrichment (TCA cycle substrates > pyruvate > sugars) can be explained by the flow of hydrogen and activated enzymes in the different conditions. Growth on sugars that activate upper EMP/ED pathways should fuel an overall ^2^H-depleted pyruvate pool, as pyruvate is predominantly synthesized by enzymes that transfer hydrogen from water with potentially large normal isotope effects (e.g., pyruvate kinase and KDPG aldolase; Figure 8; Meloche, 1975; Bollenbach et al., 1999; Wang et al., 2009; although note that the expression of such isotope effects may be diluted by any enzyme reversibility). The influence of pyruvate on ${\;}^{2}\varepsilon_{\text{AA/w}}$ values in glucose-grown organisms is visible through the correlations between leucine or valine ${\;}^{2}\varepsilon_{\text{AA/w}}$ values (which should only reflect pyruvate and water hydrogen) and carbon flux through enzymes related to pyruvate synthesis (Figure 5). In particular, the ED pathway appears to have a more ^2^H-depleting effect on pyruvate δ^2^H than does the EMP pathway, as increased flux through KDPG aldolase leads to net ^2^H-depletion of leucine and valine, while increased flux through both phosphoglucose isomerase and pyruvate kinase stimulate ^2^H-enrichment of the amino acids (Figure 5). These trends are consistent with the magnitudes of solvent isotope effects observed for enzymes in the respective pathways (1,700‰ for pyruvate kinase and 3,250‰ for KDPG aldolase; Meloche, 1975; Bollenbach et al., 1999). ^2^H-enrichment by PEP carboxykinase (anaplerotic pathway) may be due to the ^2^H-enriched hydrogen transferred from the TCA cycle into pyruvate through PEP. Transketolase influences the hydrogen isotope composition of pyruvate by combining PP pathway intermediates to produce fructose 6-phosphate and glyceraldehyde 3-phosphate, which feed into glycolysis (Supplementary Figure S2). In contrast to the wildtype microbes, mutant *E. coli* organisms mainly synthesized pyruvate via glycolysis (Supplementary Figure S4, Supplementary Table S1), resulting in similar δ^2^H_AA_ values as wildtype *E. coli*. This particular result highlights how variations in amino acid hydrogen isotope compositions are driven by differences in hydrogen routing through central metabolism rather than merely by changes in the magnitude of flux through a given enzyme.

Compared to glucose metabolism, the cellular pyruvate pool should be more ^2^H-enriched when organisms are grown on pyruvate (Figure 8), as pyruvate is directly assimilated with presumably minimal isotopic alteration, so the resulting cellular pyruvate δ^2^H value should be close to that of the starting substrate (–12‰ as measured by Zhang et al., 2009; Supplementary Table S2). Even higher cellular pyruvate δ^2^H values are likely produced in organisms grown on TCA cycle substrates (succinate, citrate, and acetate) due to the large KIEs of three differentially activated enzymes: succinate dehydrogenase, citrate synthase, and malate synthase. These enzymes abstract hydrogen from their respective substrates, leading to strong ^2^H-enrichment of residual hydrogen in the organic products (Figure 8). Succinate dehydrogenase removes one hydrogen from each methylene group in succinate with a large isotope effect (*in vitro* KIE = 5.4; Rétey et al., 1970), producing highly ^2^H-enriched fumarate. Succinate metabolism stimulates high flux through malic enzyme (Gerosa et al., 2015), which carries the ^2^H-enriched fumarate hydrogen through malate into pyruvate (although with isotopic dilution via solvent hydrogen incorporation by fumarase and malic enzyme; Figure 8), contributing to among the highest δ^2^H_AA_ values across growth conditions, including a 228‰–360‰ ^2^H-enrichment of proline in *B. subtilis* grown on succinate vs. glucose (Figure 6). This contrasts with low δ^2^H_AA_ values produced during sugar metabolism, whereby malate is predominantly routed around the TCA cycle through oxaloacetate, so the ^2^H-enriched hydrogen does not end up in pyruvate, nor in α-ketoglutarate due to stereospecific abstraction by aconitase and isocitrate dehydrogenase (Figure 8; Smith and York, 1970; Gerosa et al., 2015; Ochs and Talele, 2020). During acetate metabolism, acetyl-CoA is either routed around the TCA cycle through citrate synthase, or through the glyoxylate shunt by malate synthase, which conserves carbon by bypassing the decarboxylating steps of the TCA cycle (Zhao and Shimizu, 2003; Gerosa et al., 2015). Both citrate synthase and malate synthase abstract a hydrogen from the methyl group of acetyl-CoA with large KIEs (*in vitro* KIEs = 1.94 and 3.8, respectively; Lenz et al., 1971; O'Leary, 1989), leading to ^2^H-enriched methylene hydrogen in citrate and malate that are routed into pyruvate through malic enzyme (Figure 8). The enzymatic reactions comprising the glyoxylate shunt do not introduce water hydrogen into organic intermediates, thus ^2^H-enrichment of malate when the glyoxylate shunt is activated is likely more significant than when malate is synthesized by fumarase. Phenylalanine in *P. fluorescens* experienced the most ^2^H-enrichment from glucose to acetate metabolism (239‰–313‰), potentially in part due to transfer of ^2^H-enriched TCA cycle hydrogen into PEP through PEP carboxykinase (Gerosa et al., 2015; Dolan et al., 2020). However, in other organisms, the δ^2^H value of phenylalanine shifted by much smaller amounts. Citrate metabolism bypasses the ^2^H-enriching citrate synthase step, leading to significantly lower proline δ^2^H values in *P. fluorescens* compared to pyruvate, acetate, or succinate metabolism (Figure 6). However, flux through succinate dehydrogenase and malic enzyme still carries ^2^H-enriched hydrogen into pyruvate, likely contributing to the strong ^2^H-enrichment of the other four amino acids during citrate metabolism compared to glucose metabolism. Note that reversibility of any central metabolic enzymatic reaction contributing to equilibration of organic hydrogen with water could dilute any of the aforementioned signals.

Overall, pyruvate appears to exert an important influence on the δ^2^H values of all amino acids. The δ^2^H value of pyruvate is likely sensitive to the catabolic pathways activated for substrate degradation, which contributes to some of the variations in δ^2^H_AA_ values across substrate conditions. Thus, the hydrogen isotope compositions of amino acids—particularly leucine and valine—may be useful for identifying the types of substrates consumed, and thus metabolic pathways used, by an organism. In other words, δ^2^H_AA_ values may provide insight into how organisms process carbon from their environment. Future work should explore the mechanistic link between pyruvate (and other important central metabolites) and amino acid δ^2^H values under different metabolic conditions.

##### 4.2.1.2 NADPH δ^2^H

Proline, phenylalanine, and isoleucine derive hydrogen from NADPH (Figure 1, Supplementary Figures S16–S19). The isotopic composition of NADPH is thought to be primarily driven by the relative fluxes through dehydrogenases (which produce NADPH) in central metabolism, and through transhydrogenases that interconvert NADH and NADPH to maintain NADPH balance between catabolic and anabolic processes (Zhang et al., 2009; Wijker et al., 2019). The dehydrogenase and transhydrogenase enzymes have different KIEs (O'Leary, 1989; Bizouarn et al., 1995; Venning et al., 1998; Fjellström et al., 1999), and their fluxes vary across organisms and substrate conditions, leading to large variations in the isotopic composition of NADPH (Wijker et al., 2019). In wildtype organisms grown on glucose, these effects are visible through correlations between lipid δ^2^H values and fluxes through dehydrogenases (6PGDH, ICDH) or enzymes that directly compete with dehydrogenases (PGI, KDPG aldolase; Wijker et al., 2019). However, amino acid δ^2^H values across the same organisms grown on glucose showed no correlation with carbon flux through any NADPH-related enzyme, nor with overall NADPH imbalance flux (calculated as the difference between all NADPH-producing and -consuming fluxes; Supplementary Figure S14). In fact, despite nearly identical carbon fluxes in *E. meliloti* and *R. radiobacter* grown on glucose (Supplementary Figure S3; Supplementary Table S1), these organisms exhibited the largest differences in δ^2^H values for proline (Figure 6; Supplementary Table S2), whose hydrogen is derived from the same sources as lipids: NAD(P)H, acetyl-CoA, and water. Lack of clear control by NADPH on δ^2^H_AA_ values during glucose metabolism may be due to the fact that amino acids inherit only a small fraction of hydrogen from NADPH (13–30%) relative to other sources (e.g., ~75% of hydrogen in phenylalanine derived from PEP + erythrose 4-phosphate), so any control by NADPH may be obscured by variations in isotope compositions of the other sources. Alternatively, these results could be due to different cofactor specificities (i.e., use of NADH vs. NADPH; Fuhrer and Sauer, 2009) of biosynthetic enzymes, different isotope effects within amino acid biosynthetic pathways (Section 4.2.2.), downstream processing of amino acids after synthesis, or an outsized influence of other unknown factors on δ^2^H_AA_ values in wildtype organisms.

While the extent of NADPH influence on δ^2^H_AA_ values in wildtype organisms is unclear when comparing different organisms grown under the same condition, it is more apparent when physiological variability is controlled for—i.e., by comparing single organisms grown under different conditions. In *E. coli* wildtype and mutant organisms with perturbed NADPH metabolisms, δ^2^H values for proline, phenylalanine, and isoleucine were weakly to moderately correlated with carbon flux through NADPH-related enzymes, and δ^2^H values for isoleucine and phenylalanine positively correlated with NADPH imbalance fluxes in the cells (Supplementary Figure S15). This latter result is presumably due to increased activity of soluble transhydrogenase UdhA, which corrects NADPH overproduction by converting NADPH to NADH with an accompanying normal KIE, leading to ^2^H-enrichment of the residual NADPH pool. Surprisingly, NADPH imbalance was not correlated with proline δ^2^H values in *E. coli* organisms, possibly because these modest effects were overprinted by changes in the δ^2^H value of acetyl-CoA—the other major hydrogen source of proline. We attempted to disentangle these influences by isolating the contribution of NADPH to proline δ^2^H variations in wildtype organisms. We subtracted the relative contribution of acetyl-CoA δ^2^H variations ($\Delta^{2}{\text{H}}_{\text{AcCoA}}$) from total shifts in proline δ^2^H values ($\Delta^{2}{\text{H}}_{\text{Pro}}$) between pairs of glucose and non-glucose substrate conditions:


(3) $$\begin{array}{l} \Delta^{2}{\text{H}}_{\text{NADPH}}=\frac{7}{2}\Delta^{2}{\text{H}}_{\text{Pro}}-\Delta^{2}{\text{H}}_{\text{AcCoA}} \end{array}$$

where $\Delta^{2}{\text{H}}_{\text{NADPH}}$ is the NADPH-driven variation in proline δ^2^H. In turn, $\Delta^{2}{\text{H}}_{\text{AcCoA}}$ can be estimated based on assumptions about how hydrogen is routed through the catabolic pathways. During glucose, pyruvate, and succinate metabolism, the majority of acetyl-CoA is produced from pyruvate via pyruvate dehydrogenase (Gerosa et al., 2015), and as the methyl group remains intact, no hydrogen isotope alteration is presumed to occur (Figure 8). Thus, $\Delta^{2}{\text{H}}_{\text{AcCoA}}$ from glucose to pyruvate or succinate metabolism can be approximated as equal to shifts in cellular pyruvate δ^2^H ($\Delta^{2}{\text{H}}_{\text{Pyr}}$; Supplementary Equation S6), which in turn can be estimated through shifts in leucine or valine δ^2^H (see Supplementary Section 6.3 for details). During acetate metabolism, pyruvate hydrogen does not route into proline (Figure 8; Gerosa et al., 2015; Dolan et al., 2020), so individual cellular acetyl-CoA δ^2^H values upon growth on acetate and glucose were estimated based on measured substrate δ^2^H values and assumptions about hydrogen routing from substrates into acetyl-CoA (see Supplementary Section 6.3 for details). The magnitudes of estimated NADPH-driven ^2^H-enrichment of proline varied widely across organisms (Figure 7C). Interestingly, for *E. coli* and *B. subtilis* in particular, the magnitudes of NADPH imbalances in our glucose-grown cultures (–32 and -11%, respectively, measured in Wijker et al., 2019), as well as those from published work on *E. coli* grown on pyruvate (13%), acetate (50%), and succinate (72%; Gerosa et al., 2015; Haverkorn van Rijsewijk et al., 2016), appear to scale with the substrate ordering of NADPH-driven ^2^H-enrichment of proline (Figure 7C). In *E. coli* and *B. subtilis*, significant soluble transhydrogenase activity has been demonstrated in association with NADPH overproduction (Sauer et al., 2004; Fuhrer and Sauer, 2009; Haverkorn van Rijsewijk et al., 2016), so the potentially progressive increase in ^2^H-enrichment of the NADPH pool across growth on glucose, pyruvate, acetate, and succinate, may be contributing to the associated increase in proline (and other amino acid) δ^2^H values. Control on δ^2^H_AA_ values by NADPH metabolism may also be important in *E. meliloti, P. fluorescens*, and *R. radiobacter*, but the extent of this control is unclear due to uncertainty in NADPH balancing mechanisms in these organisms.

Overall, the isotope composition of the NADPH pool appears to exert some control on the δ^2^H values of amino acids that inherit NADPH hydrogen. Unlike for lipids, the influence of NADPH may be difficult to observe across different organisms, but more readily apparent when considering a given organism grown under different physiological conditions. In this way, primary controls on amino acid δ^2^H values are different from those for lipids, yet amino acids may offer the unique advantage of isolating the isotopic influence of NADPH—i.e., by comparing the δ^2^H values of amino acids containing vs. lacking NADPH-derived hydrogen—which may enable researchers to probe NADPH-related metabolic phenomena such as redox balance in cells.

#### 4.2.2 Enzymatic variations in biosynthetic pathways

Variations in δ^2^H_AA_ values across organisms grown on the same substrate (e.g., Figure 4) may be driven not only by differences in the organisms' catabolic fluxes and pathways employed for substrate degradation, but also by species-specific differences in the amino acid biosynthetic enzymes and their isotope effects. Throughout the evolution of amino acid biosynthetic pathways, events such as gene duplication, functional convergence, and emergence of alternative pathways have contributed to a diversity in the enzymes and mechanisms of amino acid synthesis employed across different clades and species (Hernández-Montes et al., 2008). These enzymatic variations may contribute to different isotope effects expressed at each biosynthetic step, and consequently, different net fractionations expressed for the overall biosynthetic pathways. The five microbes investigated in this study largely employ the same enzymatic reactions to synthesize their amino acids, but exhibit large variations in isozymes expressed for each step (Supplementary Figure S20). For example, *B. subtilis* expresses three isozymes of pyrroline-5-carboxylate reductase (ProG, ProH, and ProI; EC 1.5.1.2)—which transfers hydrogen from NAD(P)H to catalyze the final step of proline biosynthesis (Supplementary Figure S16)—while the other four organisms express the same single enzyme (ProC). These differences may contribute to some of the variations in δ^2^H_AA_ values across the five organisms; however, a general lack of data on isotope effects in these pathways challenges interrogation of this hypothesis in our study. Future studies that elucidate the net hydrogen isotope fractionations in each amino acid biosynthetic pathway will be invaluable in facilitating a more comprehensive, mechanistic understanding of δ^2^H_AA_ controls and variations.

### 4.3 Conclusions and potential applications

Here we have explored several hypotheses regarding biochemical controls on δ^2^H_AA_ values in aerobic heterotrophic microbes. Our results demonstrate that the overall pattern of amino acid/water fractionations is highly correlated with the individual biosynthetic pathways in organisms, while magnitudes of fractionations are likely controlled by the organic precursor and NADPH isotope compositions. In turn, the δ^2^H values of organic precursors and the NADPH pool are driven by the relative fluxes through different central metabolic pathways, which vary depending on the catabolic pathways activated for substrate degradation. Together, these results suggest that δ^2^H_AA_ values may be useful tracers for carbon processing within organisms and the environment. As the 20 biological amino acids are ubiquitous across the tree of life, and organisms that share similar biosynthetic pathways should produce similar patterns of amino acid/water fractionations, we may expect the δ^2^H values of amino acids synthesized *de novo* across microbial and metazoan taxa to be governed by the same controls. Quantitative interrogations of the hypotheses presented in this study will likely require modeling work (e.g., Mueller et al., 2022), but are needed in order to fully understand the information encoded in these signals.

While controls on δ^2^H_AA_ values are clearly nuanced and it may not be possible to uniquely relate all δ^2^H_AA_ values to simple biological or environmental properties, the different combinations of hydrogen sources in amino acids leads to rich variability in δ^2^H_AA_ signals, thus numerous potential applications of δ^2^H_AA_ analysis. For example, δ^2^H_AA_ values may provide insight into the metabolic strategies that microbes employ for carbon and energy acquisition. Microbes are the major drivers of nutrient and energy cycling in the environment, thereby playing substantial roles in shaping the geochemistry of our planet. While we observed large systematic variations in amino acid/water fractionations across heterotrophic microbes grown on different carbon substrates, we predict that even larger variations should exist between organisms of different metabolic classes (e.g., heterotrophs vs. autotrophs), as is the case for lipids (Zhang et al., 2009; Osburn et al., 2016). If true, δ^2^H_AA_ values and patterns may be used to decipher the metabolisms of unculturable organisms, to distinguish contributions by metabolically distinct organisms to geochemical processes in nature (e.g., in largely inaccessible environments such as deep subsurface biospheres), and/or to quantify contributions by different metabolisms to bulk organic matter in the environment (including by mixotrophic organisms that operate on a continuum of carbon and energy acquisition strategies, or across diverse taxa such as plants, algae, bacteria, and fungi). While lipid δ^2^H values have been suggested as a potential tool for this latter application (Cormier et al., 2022), amino acids may offer distinct advantages, as their δ^2^H values can be linked to host organisms through the isolation and sequencing of proteins (Gharibi et al., 2022b), and their different combinations of hydrogen sources capture a more diverse suite of information than is encoded in lipids (which inherit hydrogen from essentially the same three sources: acetyl-CoA, NADPH, and water). However, we view amino acid and lipid δ^2^H analyses as complementary, as each can help inform interpretations from the other. Leucine and valine δ^2^H may provide the most direct information about the metabolic ‘state' or lifestyle of an organism, as the positioning of pyruvate as a central node in metabolism makes it relatively sensitive to the molecular wiring of central metabolic pathways in cells. In turn, accounting for these metabolic signals may help isolate the NADPH-driven signals in proline and lipid δ^2^H values, thereby increasing their sensitivity as redox indicators in cells. Moreover, if the majority of phenylalanine hydrogen is indeed equilibrated with water at the level of metabolic intermediates, the δ^2^H value of phenylalanine may provide insight into water δ^2^H in places where water sources are unclear (e.g., in environments with intermittent wet/dry cycles), and if such equilibrium is temperature-dependent, phenylalanine could additionally serve as a potential bio-thermometer.

In addition to geomicrobiology-based applications, δ^2^H_AA_ analyses may provide useful information about eukaryotic organisms, including their stressors, diets, and migration patterns. The application of δ^2^H_AA_ values to human and wildlife forensics is in the early stages of exploration, with links between mammal diet, drinking water, and δ^2^H_AA_ values beginning to emerge (Newsome et al., 2020; Mancuso et al., 2023). The biochemical controls discussed here may only be relevant for interpreting δ^2^H values of non-essential amino acids (e.g., proline) as well as those with relatively high contributions from the gut microbiome (e.g., phenylalanine; Newsome et al., 2020). However, the differentiated tissues in animals, variable residence times of proteins in cells, and integration of numerous dietary hydrogen sources significantly increase the complexity of information encoded in mammalian δ^2^H_AA_ signals, which will require detailed investigations to disentangle. δ^2^H_AA_ signals in plants likely carry important physiological information as well. Like microbes, plants can synthesize all 20 proteinogenic amino acids, yet their hydrogen metabolism may be simpler to interpret, as plants derive their organic hydrogen exclusively from water. Amino acids are involved in numerous mechanisms of stress alleviation in plants (e.g., Batista-Silva et al., 2019), so their δ^2^H_AA_ values may encode information about their physiological status. For example, synthesis and degradation of proline helps maintain redox balance (i.e., the NADP/NADPH ratio) in plants and appears to facilitate drought tolerance (Sharma et al., 2011; Bhaskara et al., 2015; Batista-Silva et al., 2019). In the first step of proline catabolism, proline dehydrogenase removes a carbon-bound hydrogen atom, which should lead to ^2^H-enrichment of the residual proline pool. Consequently, proline δ^2^H values may serve as a sensitive indicator of oxidative and drought stress in plants, which are highly important aspects of crop health. This mechanism may additionally play a role in the extreme ^2^H-enrichment of proline observed in gray seals (Gharibi et al., 2022a), which endure significant periods of oxidative stress while diving. Overall, δ^2^H_AA_ analysis has potential to become a highly useful isotopic tool for a variety of diverse applications, which will undoubtedly emerge as we continue to unravel the biochemical mechanisms underpinning δ^2^H_AA_ signals in organisms.

## Data availability statement

The original contributions presented in the study are included in the article/Supplementary material, further inquiries can be directed to the corresponding author.

## Author contributions

SS: Conceptualization, Formal analysis, Funding acquisition, Investigation, Methodology, Writing—original draft, Writing—review & editing. RW: Conceptualization, Methodology, Writing—review & editing. AS: Conceptualization, Formal analysis, Funding acquisition, Methodology, Project administration, Resources, Supervision, Writing—review & editing.
