# Supplementary material for: Biosynthetic and catabolic pathways control amino acid δ2H values in aerobic heterotrophs
Source: Front Microbiol. 2024 Apr 5;15:1338486. doi: 10.3389/fmicb.2024.1338486 (PMC11026604; doi:10.3389/fmicb.2024.1338486)
Supplement: Supplementary file 1 [file Data_Sheet_1.PDF]

## *Supplementary Material*

# 1. RATIONALE FOR AMINO ACIDS TARGETED

The five amino acids investigated in this study (proline, phenylalanine, leucine, valine, and isoleucine) were selected because their hydrogen isotope compositions are among the most reliable to interpret based on preparatory and analytical considerations. These amino acids do not degrade during hydrolysis, even in the presence of oxygen (Table S4; Silverman et al., 2022), nor do they experience hydrogen exchange with aqueous medium during hydrolysis or derivatization (Figs. S9 and S10). Moreover, they exhibit consistent baseline chromatographic separation, and their high abundances in cells and great ionization efficiencies result in relatively intense chromatographic peaks that can be measured with reasonable precision (Fig. S1; Table S2).

Of the remaining 15 common biological amino acids, some remain isotopically faithful through preparation, while others may not be possible to target for $\delta^2H$ analysis. A thorough review of the preparatory and analytical considerations for isotopic analysis of each amino acid is provided in Silverman et al. (2022) and briefly summarized here in the context of our methodological approach. Lysine is stable during hydrolysis and derivatizes sufficiently to an MOC methyl ester, thus is a promising target for future $\delta^2H$ analyses. Alanine and glycine are also stable during hydrolysis but co-elute on a ZB-5ms column when derivatized as MOC methyl esters; however, these amino acids can be separated when using different derivatization reactions and/or column stationary phases (e.g., Corr et al., 2007; Walsh et al., 2014). Asparagine and glutamine quantitatively deamidate to aspartic acid and glutamic acid during hydrolysis (Wright, 1991), while the acidic amino acids and tyrosine experience moderate to significant hydrogen exchange with aqueous medium (Fig. S9; Hill and Leach, 1964), so the $\delta^2H$ values of these amino acids should be interpreted with caution. The peak shape of threonine was often problematic in our analyses (Fig. S1), potentially related to incomplete derivatization of threonine's hydroxyl group and its consequent interaction with the column stationary phase (Hušek, 1991). Histidine and serine have relatively low reaction yields when derivatized to MOC esters (Walsh et al., 2014), but the former amino acid is sometimes still accessible for analysis. Arginine is difficult to derivatize with the majority of common GC-based derivatization reactions (Silverman et al., 2022). Cysteine and methionine experience extensive oxic degradation during hydrolysis (Phillips et al., 2021), although the mechanism of loss suggests that their hydrogen isotope ratios may not be affected (Silverman et al., 2022), which was corroborated for methionine by hydrolysis tests in this study (Table S4). Tryptophan also degrades extensively during hydrolysis and is difficult to recover for most applications.

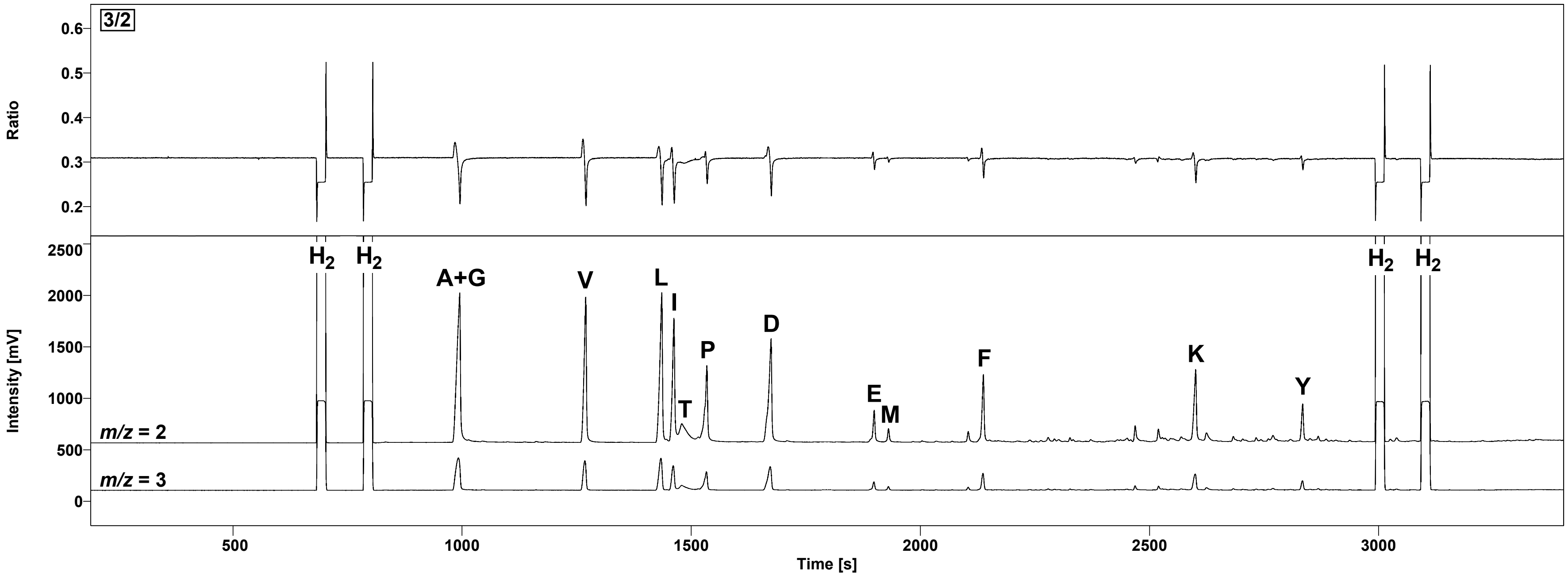


**Figure S1.** Typical chromatogram and isotope ratio trace from GC/P/IRMS analysis of amino acids from microbial biomass. The conventional single-letter amino acid codes are used to label peaks. $H_2$ reference gas with a known isotopic composition was used to calibrate amino acid hydrogen isotope ratios.

# 2. CARBON FLUXES

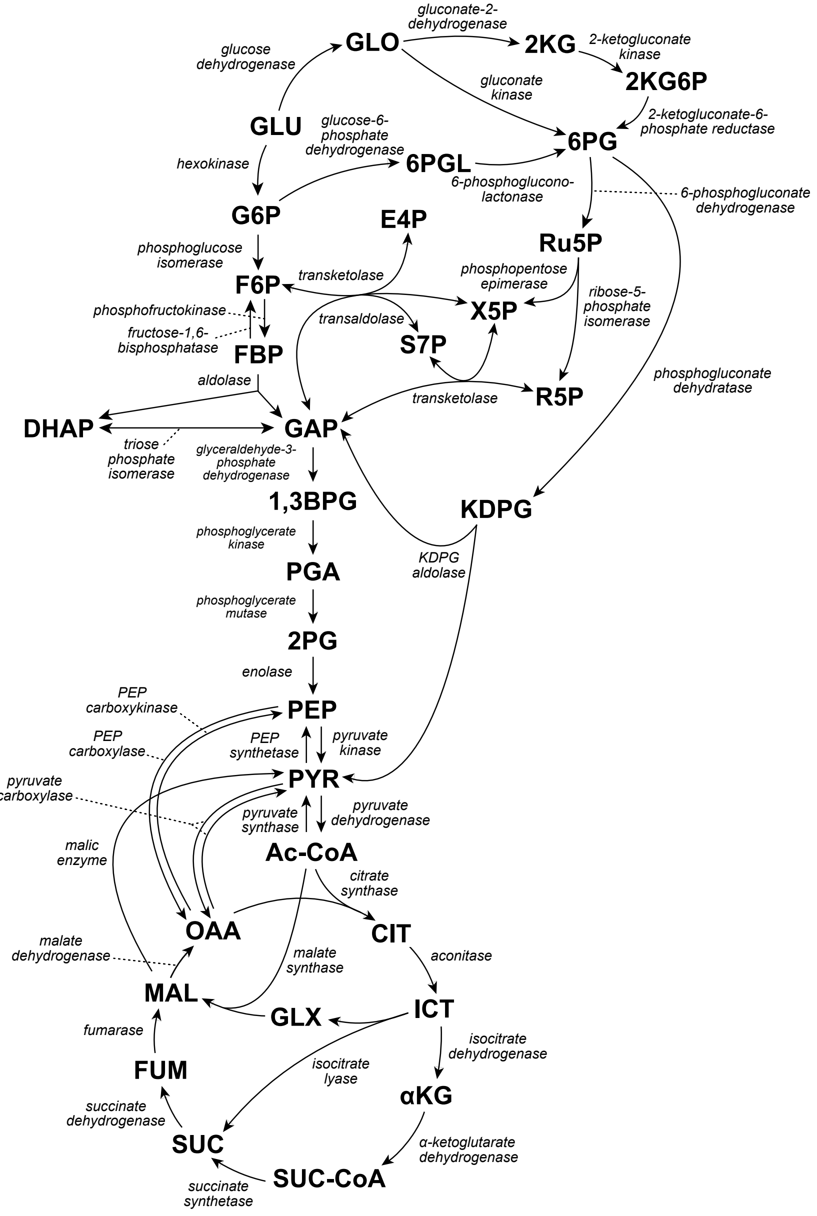


**Figure S2.** Schematic of enzymatic reactions in central metabolic pathways.

**Table S1.** Estimated net carbon fluxes of wildtype organisms and *E. coli* mutants grown on glucose, reproduced from Wijker et al. (2019). Fluxes represent reaction rates of enzymatic conversion, in mmol $h^{-1}$ $g^{-1}$ biomass.

| | Wildtype organisms | | | | |
|---|---|---|---|---|---|
| **Reaction** | ***B. subtilis*** | ***E. coli*** | ***E. meliloti*** | ***P. fluorescens*** | ***R. radiobacter*** |
| GLU + ATP → G6P | 6.65 ± 0.09 | 8.30 ± 0.10 | 3.64 ± 0.01 | 0.48 ± 0.03 | 3.88 ± 0.02 |
| GLU → GLO | - | - | - | 4.48 ± 0.09 | - |
| GLO + ATP → 6PG | - | - | - | 3.89 ± 0.09 | - |
| GLO → 2KG → 6PG + NADPH | - | - | - | 0.58 ± 0.05 | - |
| G6P → 6PG + NADPH | 2.10 ± 0.07 | 2.40 ± 0.09 | 3.63 ± 0.02 | 0.89 ± 0.06 | 3.86 ± 0.02 |
| 6PG → Ru5P + $CO_2$ + NADPH | 2.10 ± 0.07 | 1.72 ± 0.11 | 0.37 ± 0.20 | 0.38 ± 0.03 | 0.41 ± 0.18 |
| G6P → F6P | 4.36 ± 0.09 | 5.87 ± 0.12 | 0.00 ± 0.02 | −0.43 ± 0.03 | 0.00 ± 0.02 |
| 6PG → GAP + PYR | 0.00 ± 0.00 | 0.69 ± 0.12 | 3.26 ± 0.20 | 4.99 ± 0.10 | 3.45 ± 0.18 |
| F6P + ATP → 2 GAP | 5.55 ± 0.08 | 6.68 ± 0.13 | 0.04 ± 0.12 | −0.35 ± 0.02 | 0.06 ± 0.11 |
| X5P + R5P → S7P + GAP | 0.68 ± 0.02 | 0.55 ± 0.04 | 0.07 ± 0.07 | 0.11 ± 0.01 | 0.09 ± 0.06 |
| X5P + E4P → F6P + GAP | 0.51 ± 0.02 | 0.31 ± 0.04 | 0.00 ± 0.06 | 0.00 ± 0.01 | 0.00 ± 0.05 |
| S7P + GAP → E4P + F6P | 0.68 ± 0.02 | 0.55 ± 0.04 | 0.07 ± 0.07 | 0.11 ± 0.01 | 0.09 ± 0.06 |
| GAP → PGA + ATP + NADH | 11.51 ± 0.17 | 14.29 ± 0.21 | 3.31 ± 0.11 | 4.26 ± 0.09 | 3.52 ± 0.10 |
| PGA → PEP | 10.96 ± 0.17 | 13.40 ± 0.22 | 1.46 ± 0.08 | 3.72 ± 0.10 | 1.93 ± 0.08 |
| PEP → PYR + ATP | 11.95 ± 0.25 | 9.07 ± 0.76 | 0.63 ± 0.07 | 3.40 ± 0.11 | 1.20 ± 0.07 |
| PYR → AcCoA + $CO_2$ + NADH | 8.19 ± 0.16 | 9.61 ± 0.29 | 2.56 ± 0.26 | 6.00 ± 0.23 | 2.89 ± 0.25 |
| OAA + AcCoA → ICT | 1.39 ± 0.15 | 2.80 ± 0.33 | 2.01 ± 0.29 | 5.18 ± 0.24 | 2.16 ± 0.29 |
| ICT → αKG + $CO_2$ + NADPH | 1.39 ± 0.15 | 2.80 ± 0.33 | 2.01 ± 0.29 | 5.18 ± 0.24 | 2.16 ± 0.29 |
| αKG → FUM + $CO_2$ + 1.5 ATP + 2 NADH | 0.90 ± 0.15 | 1.89 ± 0.35 | 1.75 ± 0.32 | 4.62 ± 0.26 | 1.81 ± 0.31 |
| FUM → MAL | 0.90 ± 0.15 | 1.89 ± 0.35 | 1.75 ± 0.32 | 4.62 ± 0.26 | 1.81 ± 0.31 |
| MAL → OAA + NADH | 0.66 ± 0.06 | 0.00 ± 0.58 | 1.60 ± 0.31 | 1.80 ± 0.10 | 1.71 ± 0.31 |
| MAL → PYR + $CO_2$ + NADPH | 0.24 ± 0.12 | 1.89 ± 0.91 | 0.15 ± 0.05 | 2.82 ± 0.20 | 0.11 ± 0.06 |
| OAA + ATP → PEP + $CO_2$ | 1.35 ± 0.14 | 0.48 ± 0.14 | 0.00 ± 0.01 | - | 0.00 ± 0.02 |
| PEP + $CO_2$ → OAA | - | 4.28 ± 0.85 | - | - | - |
| PYR + ATP + $CO_2$ → OAA | 2.82 ± 0.19 | - | 0.72 ± 0.11 | 4.99 ± 0.21 | 0.88 ± 0.11 |
| OAA → PYR + $CO_2$ | - | - | - | 1.02 ± 0.13 | - |
| AcCoA → Acetate + ATP | 6.19 ± 0.09 | 5.39 ± 0.10 | - | - | - |
| NADH → NADPH | 0.72 ± 0.21 | 2.67 ± 0.98 | - | - | - |
| NADPH → NADH | - | - | 1.39 ± 0.36 | 0.42 ± 0.32 | 0.38 ± 0.34 |
| Respiration | 11.75 ± 0.10 | 15.42 ± 0.73 | 5.85 ± 0.58 | 12.4 ± 0.51 | 6.31 ± 0.56 |

| Reaction | *E. coli* mutant organisms | | | | |
|---|---|---|---|---|---|
| | **JW1841** | **JW3985** | **PntAB** | **UdhA** | **UdhA-PntAB** |
| GLU + ATP → G6P | 7.97 ± 0.10 | 4.86 ± 0.10 | 7.10 ± 0.10 | 10.38 ± 0.10 | 7.16 ± 0.10 |
| GLU → GLO | - | - | - | - | - |
| GLO + ATP → 6PG | - | - | - | - | - |
| GLO → 2KG → 6PG + NADPH | - | - | - | - | - |
| G6P → 6PG + NADPH | 0.77 ± 0.10 | 4.83 ± 0.10 | 2.75 ± 0.08 | 2.70 ± 0.12 | 3.10 ± 0.08 |
| 6PG → Ru5P + $CO_2$ + NADPH | 0.53 ± 0.10 | 3.05 ± 0.11 | 2.03 ± 0.09 | 1.86 ± 0.14 | 2.52 ± 0.10 |
| G6P → F6P | 7.18 ± 0.14 | 0.02 ± 0.04 | 4.33 ± 0.1 | 7.64 ± 0.14 | 4.03 ± 0.09 |
| 6PG → GAP + PYR | 0.24 ± 0.13 | 1.78 ± 0.09 | 0.71 ± 0.1 | 0.84 ± 0.16 | 0.58 ± 0.10 |
| F6P + ATP → 2 GAP | 7.33 ± 0.15 | 1.92 ± 0.07 | 5.42 ± 0.11 | 8.53 ± 0.15 | 5.46 ± 0.11 |
| X5P + R5P → S7P + GAP | 0.17 ± 0.03 | 1.01 ± 0.03 | 0.66 ± 0.03 | 0.60 ± 0.05 | 0.83 ± 0.03 |
| X5P + E4P → F6P + GAP | 0.01 ± 0.04 | 0.90 ± 0.04 | 0.45 ± 0.03 | 0.34 ± 0.05 | 0.63 ± 0.03 |
| S7P + GAP → E4P + F6P | 0.17 ± 0.03 | 1.01 ± 0.04 | 0.66 ± 0.03 | 0.60 ± 0.05 | 0.82 ± 0.03 |
| GAP → PGA + ATP + NADH | 14.88 ± 0.23 | 6.49 ± 0.15 | 11.96 ± 0.21 | 18.16 ± 0.23 | 12.10 ± 0.20 |
| PGA → PEP | 14.31 ± 0.24 | 6.11 ± 0.16 | 11.10 ± 0.21 | 17.20 ± 0.24 | 11.40 ± 0.21 |
| PEP → PYR + ATP | 9.81 ± 0.86 | 1.18 ± 0.45 | 7.71 ± 2.32 | 11.48 ± 0.85 | 8.44 ± 1.04 |
| PYR → AcCoA + $CO_2$ + NADH | 11.57 ± 0.30 | 5.96 ± 0.30 | 6.53 ± 0.30 | 12.99 ± 0.29 | 8.36 ± 0.29 |
| OAA + AcCoA → ICT | 3.50 ± 0.34 | 4.28 ± 0.34 | 1.86 ± 0.34 | 4.02 ± 0.33 | 1.75 ± 0.33 |
| ICT → αKG + $CO_2$ + NADPH | 3.50 ± 0.34 | 4.28 ± 0.34 | 1.86 ± 0.34 | 4.02 ± 0.33 | 1.75 ± 0.33 |
| αKG → FUM + $CO_2$ + 1.5 ATP + 2 NADH | 2.88 ± 0.36 | 3.88 ± 0.36 | 0.71 ± 0.36 | 2.98 ± 0.36 | 1.01 ± 0.36 |
| FUM → MAL | 2.88 ± 0.36 | 3.88 ± 0.36 | 0.71 ± 0.36 | 2.98 ± 0.36 | 1.01 ± 0.36 |
| MAL → OAA + NADH | 0.00 ± 0.68 | 0.00 ± 0.27 | 0.00 ± 2.14 | 0.00 ± 0.67 | 0.00 ± 0.85 |
| MAL → PYR + $CO_2$ + NADPH | 2.88 ± 1.00 | 3.88 ± 0.56 | 0.71 ± 2.49 | 2.98 ± 0.98 | 1.01 ± 1.19 |
| OAA + ATP → PEP + $CO_2$ | 2.48 ± 0.20 | 0.19 ± 0.07 | 0.67 ± 0.12 | 1.00 ± 0.19 | 0.53 ± 0.12 |
| PEP + $CO_2$ → OAA | 6.64 ± 0.95 | 4.90 ± 0.50 | 3.59 ± 2.44 | 6.15 ± 0.93 | 3.08 ± 1.14 |
| PYR + ATP + $CO_2$ → OAA | - | - | - | - | - |
| OAA → PYR + $CO_2$ | - | - | - | - | - |
| AcCoA → Acetate + ATP | 7.16 ± 0.10 | 1.09 ± 0.10 | 3.15 ± 0.10 | 7.40 ± 0.10 | 5.50 ± 0.10 |
| NADH → NADPH | 1.21 ± 1.07 | - | 0.54 ± 2.52 | 1.44 ± 1.06 | - |
| NADPH → NADH | - | 7.97 ± 0.67 | - | - | 0.02 ± 1.25 |
| Respiration | 17.92 ± 0.75 | 12.3 ± 0.72 | 10.77 ± 0.73 | 20.71 ± 0.73 | 12.21 ± 0.73 |

- indicates an absent reaction

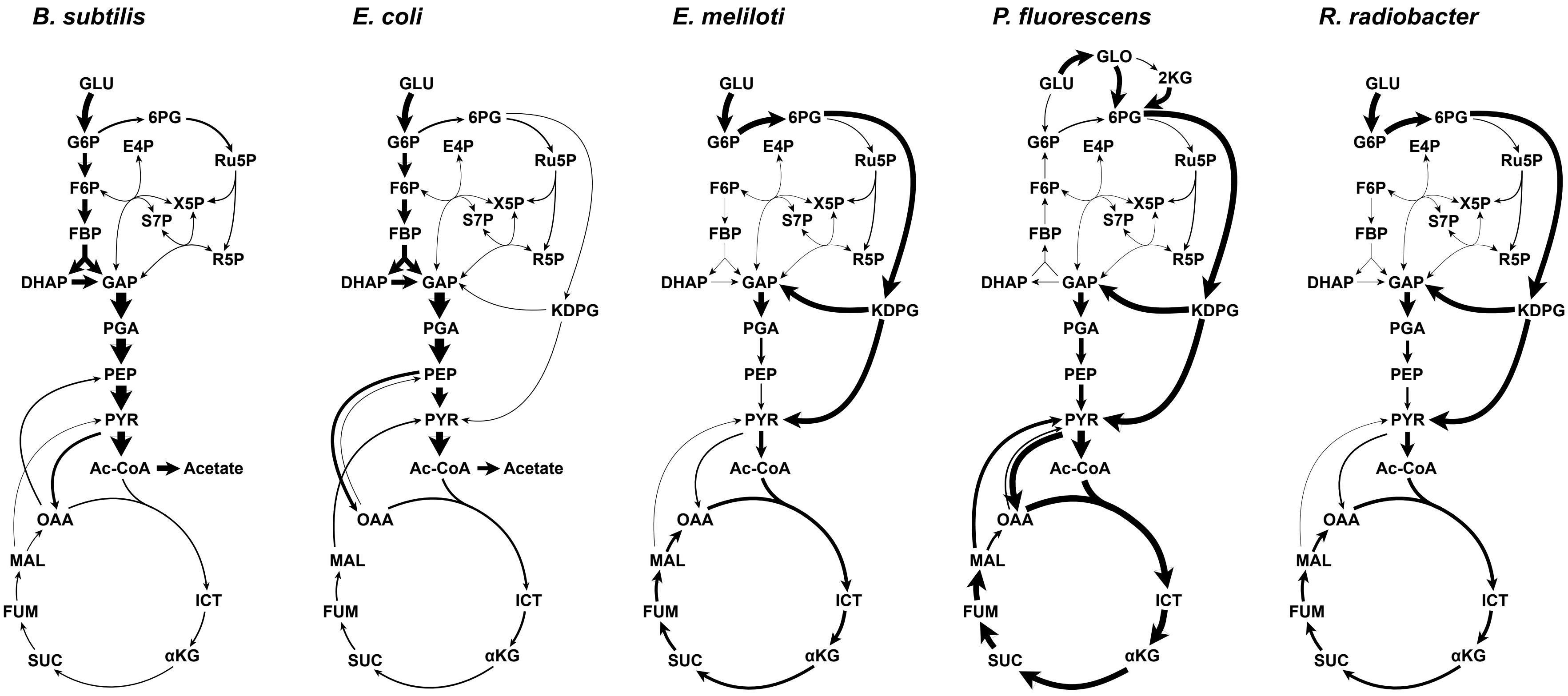


**Figure S3.** Visual summary of $^{13}C$-based metabolic fluxes in wildtype organisms grown on glucose. Sizes of arrows in flux maps scale with relative flux magnitudes (normalized to organism-specific glucose uptake rates). Flux data is summarized in Table S1.

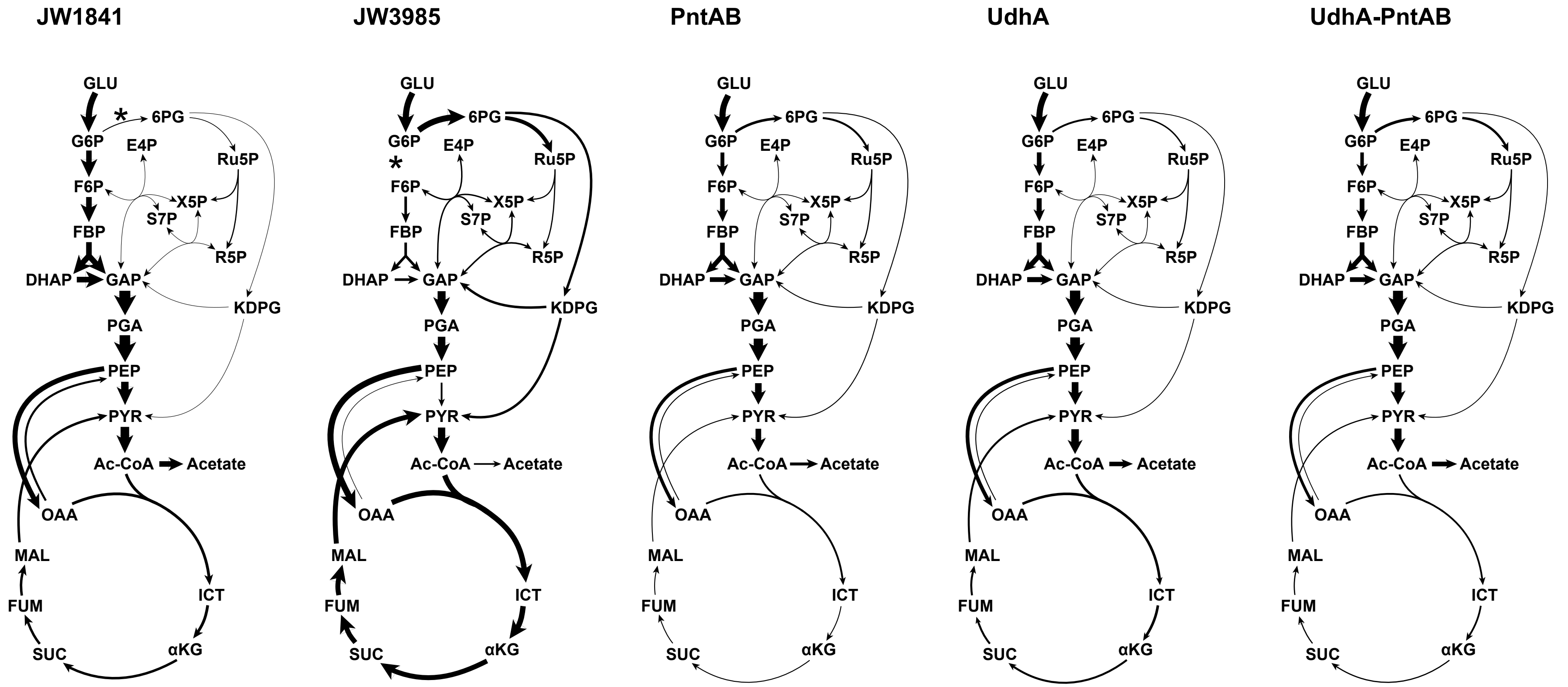


**Figure S4.** Visual summary of $^{13}C$-based metabolic fluxes in *E. coli* mutant organisms grown on glucose lacking certain dehydrogenase or transhydrogenase genes (glucose 6-phosphate dehydrogenase was deleted in JW1841, phosphoglucose isomerase in JW3985, membrane-bound transhydrogenase in PntAB, soluble transhydrogenase in UdhA, and both transhydrogenases in UdhA-PntAB). The * marks deleted genes in JW1841 and JW3985. Sizes of arrows in flux maps scale with relative flux magnitudes (normalized to organism-specific glucose uptake rates). Flux data is summarized in Table S1.

## 3. AMINO ACID $\delta^2H$ VALUES

**Table S2.** Growth rates and measured $\delta^2H$ values of amino acids (AA), substrates (s), and culture media (w) for each growth condition. Amino acids included are those whose isotopic compositions are discussed in the main text.

| Culture | Substrate | Rep.[1] | $\mu$ ($h^{-1}$)[2] | $\delta^2H_s$ (‰)[3] | $\delta^2H_w \pm \sigma$ (‰) | $\delta^2H_{AA} \pm \sigma$ (‰) | | | | |
|---|---|---|---|---|---|---|---|---|---|---|
| | | | | | | **Proline** | **Phenylalanine** | **Leucine** | **Valine** | **Isoleucine** |
| **Wildtype organisms** | | | | | | | | | | |
| *B. subtilis* | glucose | 1 | 0.38 | - | -90 ± 0 | -96 ± 2 | -134 ± 5 | -181 ± 3 | -200 ± 4 | -289 ± 2 |
| | glucose | 2 | - | -60 | -87 ± 0 | -116 ± 2 | -105 ± 16 | -166 ± 9 | -192 ± 10 | -295 ± 9 |
| | pyruvate | 1 | 0.13 | -12 | -86 ± 0 | 40 ± 2 | -127 ± 6 | -111 ± 3 | -109 ± 7 | -202 ± 6 |
| | pyruvate | 2 | 0.11 | -12 | -86 ± 0 | 34 ± 2 | -108 ± 7 | -110 ± 3 | -103 ± 10 | -198 ± 8 |
| | succinate | 1 | 0.09 | -97 | -88 ± 0 | 211 ± 4 | -81 ± 3 | -76 ± 3 | -69 ± 4 | -175 ± 3 |
| | succinate | 2 | 0.08 | -97 | -88 ± 0 | 113 ± 8 | -120 ± 5 | -70 ± 6 | -70 ± 4 | -193 ± 10 |
| *E. coli* | acetate | 1 | - | -76 | -91 ± 1 | 44 ± 5 | -47 ± 3 | -121 ± 2 | -129 ± 3 | -271 ± 8 |
| | glucose | 1 | 0.57 | - | -88 ± 1 | -90 ± 3 | -88 ± 4 | -213 ± 5 | -243 ± 7 | -392 ± 1 |
| | glucose | 2 | 0.58 | -60 | -80 ± 0 | -147 ± 2 | -123 ± 2 | -244 ± 4 | -295 ± 1 | -397 ± 7 |
| | pyruvate | 1 | - | -12 | -91 ± 1 | -27 ± 10 | -102 ± 5 | -165 ± 6 | -175 ± 7 | -325 ± 4 |
| *E. meliloti* | acetate | 1 | - | -76 | -76 ± 1 | -4 ± 2 | -12 ± 2 | -93 ± 1 | -175 ± 1 | -193 ± 2 |
| | acetate | 2 | - | -76 | -76 ± 1 | -6 ± 10 | 1 ± 6 | -88 ± 2 | -174 ± 13 | -199 ± 10 |
| | fructose | 1 | - | -22 | -85 ± 1 | -123 ± 8 | -78 ± 4 | -222 ± 5 | -324 ± 18 | -284 ± 14 |
| | fructose | 2 | - | -22 | -85 ± 1 | -120 ± 6 | -67 ± 15 | -218 ± 3 | -321 ± 4 | -282 ± 13 |
| | glucose | 1 | 0.17 | - | -76 ± 1 | -109 ± 3 | -83 ± 4 | -237 ± 1 | -356 ± 3 | -291 ± 2 |
| | glucose | 2 | - | -60 | -83 ± 1 | -135 ± 23 | -64 ± 7 | -227 ± 11 | -357 ± 14 | -307 ± 17 |
| | pyruvate | 1 | - | -12 | -77 ± 0 | -28 ± 4 | -50 ± 10 | -80 ± 3 | -156 ± 11 | -157 ± 4 |
| | pyruvate | 2 | - | -12 | -77 ± 0 | -36 ± 9 | -51 ± 3 | -86 ± 2 | -153 ± 5 | -157 ± 5 |
| | succinate | 1 | - | -97 | -83 ± 0 | -60 ± 2 | -54 ± 4 | -87 ± 2 | -166 ± 11 | -165 ± 5 |
| | succinate | 2 | - | -97 | -83 ± 0 | -32 ± 6 | -44 ± 3 | -81 ± 8 | -154 ± 8 | -155 ± 5 |
| *P. fluorescens* | acetate | 1 | - | -76 | -87 ± 0 | 15 ± 3 | 166 ± 7 | -73 ± 2 | -111 ± 3 | -30 ± 2 |
| | acetate | 2 | - | -76 | -87 ± 1 | 35 ± 9 | 178 ± 11 | -55 ± 3 | -96 ± 5 | -61 ± 10 |
| | citrate | 1 | 0.52 | - | -88 ± 0 | -65 ± 12 | 43 ± 9 | -92 ± 6 | -136 ± 11 | -192 ± 7 |

| | | | | | | | | | | |
|---|---|---|---|---|---|---|---|---|---|---|
| | citrate | 2 | 0.53 | - | -88 ± 0 | -66 ± 13 | 23 ± 17 | -102 ± 10 | -144 ± 10 | -177 ± 8 |
| | fructose | 1 | 0.28 | -22 | -87 ± 0 | -70 ± 5 | -40 ± 10 | -195 ± 2 | -269 ± 5 | -299 ± 6 |
| | fructose | 2 | 0.28 | -22 | -87 ± 0 | -54 ± 8 | -43 ± 3 | -198 ± 3 | -269 ± 7 | -307 ± 8 |
| | glucose | 1 | 0.31 | - | -82 ± 0 | -90 ± 4 | -103 ± 6 | -227 ± 3 | -308 ± 4 | -287 ± 1 |
| | glucose | 2 | - | -60 | -88 ± 1 | -77 ± 2 | -53 ± 1 | -225 ± 14 | -321 ± 28 | -252 ± 3 |
| | pyruvate | 1 | 0.38 | -12 | -87 ± 0 | -6 ± 3 | -38 ± 7 | -136 ± 4 | -185 ± 9 | -182 ± 9 |
| | pyruvate | 2 | 0.42 | -12 | -87 ± 0 | 9 ± 5 | -31 ± 3 | -126 ± 3 | -170 ± 10 | -171 ± 4 |
| | succinate | 1 | 0.46 | -97 | -86 ± 0 | 84 ± 4 | 39 ± 9 | -72 ± 3 | -104 ± 10 | -72 ± 3 |
| | succinate | 2 | 0.45 | -97 | -86 ± 0 | 77 ± 3 | 46 ± 11 | -75 ± 8 | -113 ± 8 | -81 ± 2 |
| *R. radiobacter* | acetate | 1 | - | -76 | -89 ± 1 | 29 ± 3 | 14 ± 3 | -115 ± 1 | -183 ± 4 | -232 ± 1 |
| | acetate | 2 | - | -76 | -89 ± 1 | 19 ± 6 | 22 ± 11 | -117 ± 3 | -178 ± 21 | -214 ± 11 |
| | fructose | 1 | - | -22 | -88 ± 1 | -87 ± 11 | -14 ± 18 | -242 ± 2 | -327 ± 14 | -318 ± 10 |
| | fructose | 2 | - | -22 | -88 ± 1 | -86 ± 11 | -32 ± 10 | -250 ± 2 | -337 ± 7 | -309 ± 12 |
| | glucose | 1 | 0.3 | - | -82 ± 1 | -72 ± 3 | -40 ± 3 | -246 ± 1 | -338 ± 1 | -318 ± 2 |
| | glucose | 2 | - | -60 | -90 ± 0 | -92 ± 3 | -33 ± 7 | -242 ± 12 | -353 ± 10 | -340 ± 1 |
| | pyruvate | 1 | - | -12 | -87 ± 1 | -10 ± 5 | -15 ± 4 | -128 ± 4 | -183 ± 3 | -209 ± 4 |
| | pyruvate | 2 | - | -12 | -88 ± 1 | 3 ± 2 | -26 ± 6 | -131 ± 6 | -185 ± 13 | -209 ± 4 |
| | succinate | 1 | - | -97 | -85 ± 0 | -48 ± 6 | -41 ± 11 | -118 ± 9 | -174 ± 13 | -219 ± 9 |
| | succinate | 2 | - | -97 | -87 ± 0 | -13 ± 4 | -45 ± 8 | -119 ± 10 | -170 ± 10 | -216 ± 9 |
| ***E. coli* knockout mutants** | | | | | | | | | | |
| JW1841 | glucose | 1 | 0.34 | - | -84 ± 2 | -108 ± 3 | -84 ± 3 | -219 ± 4 | -252 ± 5 | -384 ± 2 |
| JW3985 | glucose | 1 | 0.24 | - | -85 ± 1 | -78 ± 3 | -75 ± 3 | -224 ± 2 | -246 ± 6 | -378 ± 2 |
| PntAB | glucose | 1 | 0.33 | - | -74 ± 1 | -69 ± 5 | -63 ± 3 | -201 ± 3 | -238 ± 3 | -363 ± 4 |
| UdhA | glucose | 1 | 0.58 | - | -90 ± 2 | -103 ± 4 | -91 ± 4 | -220 ± 1 | -256 ± 2 | -389 ± 1 |
| UdhA-PntAB | glucose | 1 | 0.42 | - | -90 ± 2 | -92 ± 8 | -72 ± 6 | -212 ± 1 | -247 ± 7 | -378 ± 1 |
| **Growth water experiments** | | | | | | | | | | |
| *B. subtilis* | glucose | 2 | - | -60 | -87 ± 0 | -116 ± 2 | -105 ± 16 | -166 ± 9 | -192 ± 10 | -295 ± 9 |
| | | 1 | - | -60 | 194 ± 1 | 55 ± 6 | 12 ± 6 | -37 ± 6 | -94 ± 7 | -173 ± 3 |
| | | 1 | - | -60 | 448 ± 1 | 224 ± 23 | 76 ± 10 | 99 ± 10 | 7 ± 21 | -68 ± 4 |
| | | 1 | - | -60 | 618 ± 1 | 329 ± 6 | 127 ± 2 | 173 ± 1 | 74 ± 11 | 23 ± 6 |

| | | | | | | | | | | |
|---|---|---|---|---|---|---|---|---|---|---|
| | | 1 | - | -60 | 911 ± 0 | 497 ± 1 | 226 ± 3 | 296 ± 2 | 159 ± 1 | 152 ± 28 |
| *E. coli* | glucose | 2 | 0.58 | -60 | -80 ± 0 | -147 ± 2 | -123 ± 2 | -244 ± 4 | -295 ± 1 | -397 ± 7 |
| | | 1 | 0.59 | -60 | 128 ± 0 | -11 ± 8 | -28 ± 1 | -142 ± 7 | -218 ± 2 | -318 ± 2 |
| | | 1 | 0.60 | -60 | 337 ± 0 | 104 ± 8 | 35 ± 2 | -65 ± 8 | -132 ± 8 | -232 ± 2 |
| | | 1 | 0.59 | -60 | 545 ± 0 | 244 ± 3 | 101 ± 2 | 42 ± 3 | -71 ± 4 | -151 ± 8 |
| *E. meliloti* | glucose | 2 | - | -60 | -83 ± 1 | -135 ± 23 | -64 ± 7 | -227 ± 11 | -357 ± 14 | -307 ± 17 |
| | | 1 | - | -60 | 111 ± 2 | 10 ± 12 | -7 ± 5 | -133 ± 6 | -289 ± 17 | -207 ± 8 |
| | | 1 | - | -60 | 248 ± 1 | 111 ± 3 | 46 ± 6 | -48 ± 8 | -233 ± 20 | -117 ± 17 |
| | | 1 | - | -60 | 405 ± 1 | 246 ± 12 | 106 ± 14 | 36 ± 5 | -168 ± 1 | -62 ± 5 |
| | | 1 | - | -60 | 544 ± 1 | 337 ± 12 | 146 ± 8 | 121 ± 8 | -103 ± 21 | 36 ± 24 |
| *P. fluorescens* | glucose | 2 | - | -60 | -88 ± 1 | -77 ± 2 | -53 ± 1 | -225 ± 14 | -321 ± 28 | -252 ± 3 |
| | | 1 | - | -60 | 319 ± 1 | 268 ± 15 | 102 ± 9 | 27 ± 16 | -103 ± 22 | -21 ± 2 |
| | | 1 | - | -60 | 643 ± 1 | 521 ± 14 | 297 ± 14 | 244 ± 3 | 84 ± 9 | 198 ± 11 |
| | | 1 | - | -60 | 868 ± 1 | 654 ± 5 | 367 ± 11 | 375 ± 4 | 223 ± 3 | 326 ± 5 |
| *R. radiobacter* | glucose | 2 | - | -60 | -90 ± 0 | -92 ± 3 | -33 ± 7 | -242 ± 12 | -353 ± 10 | -340 ± 1 |
| | | 1 | - | -60 | 100 ± 1 | 50 ± 6 | 42 ± 3 | -151 ± 9 | -293 ± 11 | -247 ± 11 |
| | | 1 | - | -60 | 253 ± 0 | 165 ± 8 | 92 ± 2 | -67 ± 12 | -231 ± 15 | -179 ± 12 |
| | | 1 | - | -60 | 497 ± 0 | 279 ± 5 | 151 ± 5 | 11 ± 6 | -170 ± 5 | -118 ± 10 |
| | | 1 | - | -60 | 632 ± 2 | 391 ± 3 | 243 ± 11 | 87 ± 2 | -117 ± 3 | -39 ± 22 |

[1]Biological replicates are designated as 1 and 2. Data for the most $^{2}$H-depleted growth water condition for each organism are included as the second glucose replicate within the 'Wildtype organisms' conditions. Note that *E. coli* was cultured on glucose in two different experiments, so these cultures are not biological replicates (see Sections 2.1 and 2.2 in the main text).
[2]Blank entries (-) indicate growth rate was not measured.
[3]Entries with isotopic compositions are the same substrates used and measured by Zhang et al. (2009). Uncertainties are likely <20‰. Blank entries (-) indicate substrate isotopic composition was not measured.

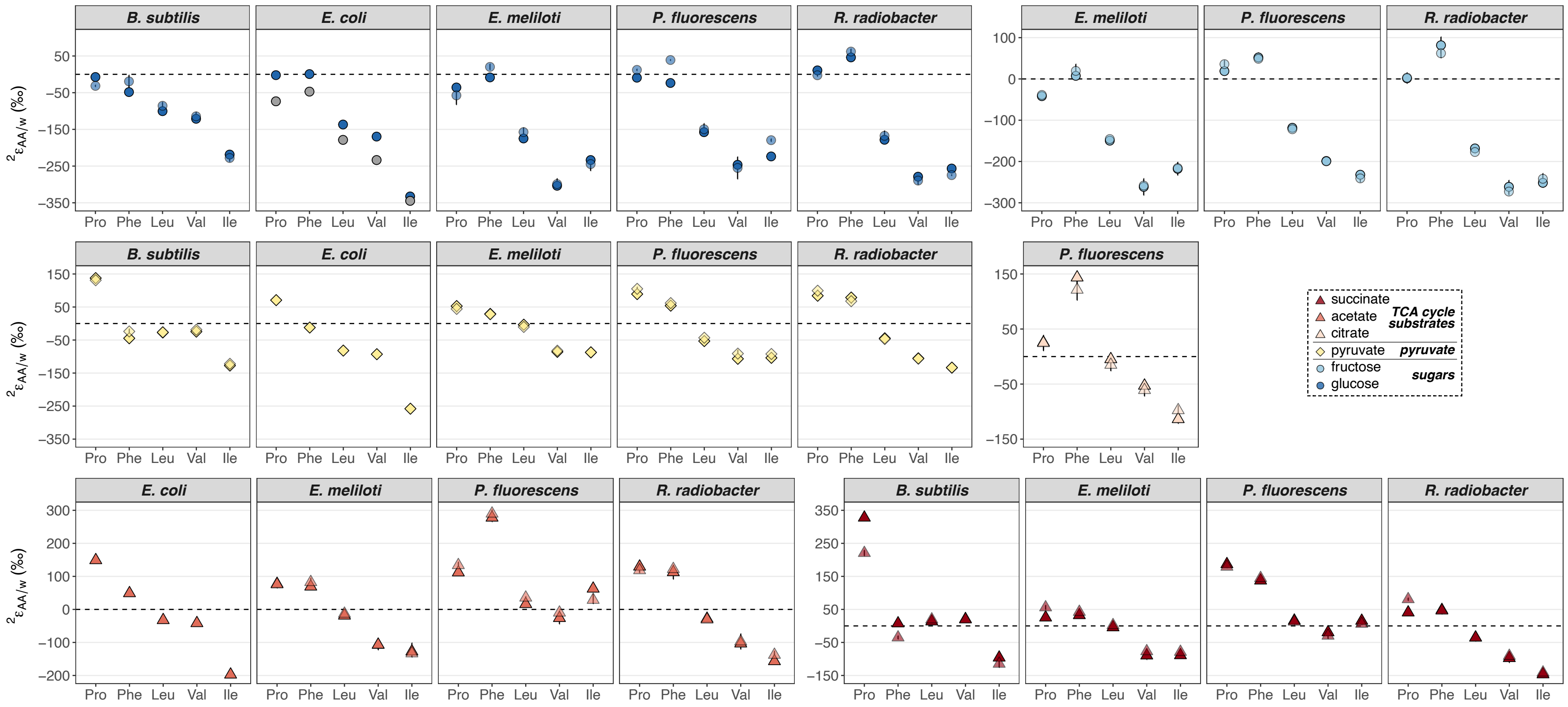


**Figure S5.** $^2H/^1H$ fractionations between amino acids and water in from biological replicates of wildtype organisms grown on different carbon substrates. Data for replicate #1 are denoted with opaque symbols, and with transparent symbols for replicate #2. Error bars indicate the propagated uncertainties (±1σ) from the amino acid, derivative, and water $\delta^2H$ measurements, and in most cases are smaller than symbols. Note that *E. coli* was cultured on glucose in two different experiments, and the amino acids were derivatized via different reaction conditions, so these cultures are not biological replicates and are distinguished as blue (culture #1) and gray (culture #2) symbols (see Sections 2.1 and 2.2 in the main text for details).

**Table S3.** δ$^2$H values of amino acids (AA) that were not discussed in the main text. See Table S2 for growth rates and δ$^2$H values of substrates and water in each culture.

| Culture | Substrate | Rep.[1] | $\delta^2H_w \pm \sigma$ (‰) | $\delta^2H_{AA} \pm \sigma$ (‰)[2] | | | | | | |
|---|---|---|---|---|---|---|---|---|---|---|
| | | | | Alanine + Glycine | Aspartic acid (+ Asparagine) | Glutamic acid (+ Glutamine) | Lysine | Methionine | Threonine | Tyrosine |
| **Wildtype organisms** | | | | | | | | | | |
| *B. subtilis* | glucose | 1 | -90 ± 0 | -177 ± 9 | -52 ± 29 | -45 ± 5 | - | -136 ± 3 | - | - |
| | glucose | 2 | -87 ± 0 | -178 ± 43 | -62 ± 5 | -49 ± 2 | -126 ± 28 | 23 | - | - |
| | pyruvate | 1 | -86 ± 0 | -111 ± 10 | -6 ± 27 | 23 ± 24 | -77 ± 18 | -100 ± 46 | - | -85 ± 38 |
| | pyruvate | 2 | -86 ± 0 | -104 ± 7 | -15 ± 31 | 14 ± 8 | -73 ± 8 | - | - | -65 ± 25 |
| | succinate | 1 | -88 ± 0 | -56 ± 9 | 72 ± 9 | 22 ± 8 | - | -54 ± 6 | -24 ± 22 | - |
| | succinate | 2 | -88 ± 0 | -91 ± 11 | -1 ± 14 | -39 ± 24 | -69 ± 13 | - | - | -48 ± 42 |
| *E. coli* | acetate | 1 | -91 ± 1 | -146 ± 7 | 41 ± 10 | 8 ± 3 | - | -10 ± 1 | - | - |
| | glucose | 1 | -88 ± 1 | -237 ± 5 | -41 ± 9 | -48 ± 18 | -143 ± 4 | -157 ± 15 | -267 | -104 ± 13 |
| | glucose | 2 | -80 ± 0 | -394 ± 6 | -198 ± 9 | - | - | - | - | - |
| | pyruvate | 1 | -91 ± 1 | -172 ± 7 | -36 ± 16 | -25 ± 30 | -100 ± 8 | -101 ± 45 | -112 | -123 ± 17 |
| *E. meliloti* | acetate | 1 | -76 ± 1 | -128 ± 44 | 51 ± 5 | 25 ± 8 | -25 ± 3 | 40 ± 18 | 53 ± 64 | - |
| | acetate | 2 | -76 ± 1 | -133 ± 39 | 60 ± 16 | 24 ± 25 | -29 ± 11 | 11 ± 28 | 212 ± 118 | - |
| | fructose | 1 | -85 ± 1 | -225 ± 69 | -12 ± 6 | -44 ± 3 | -109 ± 12 | -40 ± 8 | - | - |
| | fructose | 2 | -85 ± 1 | -238 ± 69 | -22 ± 17 | -55 ± 10 | -106 ± 25 | - | -99 | - |
| | glucose | 1 | -76 ± 1 | -240 ± 15 | -6 ± 7 | -44 ± 8 | - | - | 13 ± 4 | - |
| | glucose | 2 | -83 ± 1 | -237 ± 43 | -31 ± 10 | -24 ± 10 | -117 ± 16 | -28 ± 10 | - | - |
| | pyruvate | 1 | -77 ± 0 | -108 ± 6 | 4 ± 5 | -9 ± 15 | -55 ± 8 | -64 ± 47 | - | -73 ± 43 |
| | pyruvate | 2 | -77 ± 0 | -106 ± 7 | -8 ± 11 | 9 ± 46 | -60 ± 12 | -76 ± 55 | -82 | -52 ± 17 |
| | succinate | 1 | -83 ± 0 | -139 ± 8 | 2 ± 11 | -31 ± 23 | -68 ± 4 | -96 ± 21 | - | -73 ± 13 |
| | succinate | 2 | -83 ± 0 | -131 ± 18 | -6 ± 22 | -5 ± 39 | -71 ± 9 | - | - | -48 ± 30 |
| *P. fluorescens* | acetate | 1 | -87 ± 0 | -176 ± 3 | 46 ± 11 | 13 ± 14 | 114 ± 5 | 250 ± 31 | - | - |
| | acetate | 2 | -87 ± 1 | -176 ± 50 | 34 ± 17 | 13 ± 14 | 114 ± 7 | 211 ± 42 | - | - |
| | citrate | 1 | -88 ± 0 | -213 ± 51 | 73 ± 4 | 4 ± 14 | 1 ± 11 | 95 ± 3 | - | - |
| | citrate | 2 | -88 ± 0 | -213 ± 65 | 63 ± 5 | -13 ± 7 | -3 ± 11 | - | - | - |
| | fructose | 1 | -87 ± 0 | -290 ± 75 | -24 ± 6 | -13 ± 5 | -41 ± 18 | 6 ± 8 | 94 | - |

| | | | | | | | | | | |
|---|---|---|---|---|---|---|---|---|---|---|
| | fructose | 2 | -87 ± 0 | -290 ± 83 | -18 ± 14 | -32 ± 20 | -52 ± 8 | - | - | - |
| | glucose | 1 | -82 ± 0 | -257 ± 4 | -57 ± 3 | -28 ± 17 | - | - | - | - |
| | glucose | 2 | -88 ± 1 | -281 ± 39 | -55 ± 20 | -41 ± 3 | -61 ± 20 | 38 ± 20 | - | - |
| | pyruvate | 1 | -87 ± 0 | -233 ± 9 | -13 ± 29 | -7 ± 20 | -11 ± 16 | - | - | -35 ± 18 |
| | pyruvate | 2 | -87 ± 0 | -230 ± 10 | 20 ± 19 | -17 ± 23 | -2 ± 13 | - | - | -59 ± 28 |
| | succinate | 1 | -86 ± 0 | -195 ± 7 | 19 ± 23 | 8 ± 35 | 64 ± 4 | 136 ± 84 | - | -2 ± 14 |
| | succinate | 2 | -86 ± 0 | -201 ± 7 | 11 ± 24 | 21 ± 38 | 38 ± 11 | - | - | -3 ± 13 |
| *R. radiobacter* | acetate | 1 | -89 ± 1 | -145 ± 16 | 41 ± 6 | 49 ± 3 | -47 ± 21 | -13 ± 4 | -72 ± 7 | - |
| | acetate | 2 | -89 ± 1 | -141 ± 36 | 55 ± 4 | 37 ± 36 | -51 ± 17 | 45 ± 29 | 110 ± 58 | - |
| | fructose | 1 | -88 ± 1 | -222 ± 44 | -40 ± 6 | -19 ± 15 | -105 ± 11 | -45 ± 33 | 112 ± 72 | - |
| | fructose | 2 | -88 ± 1 | -224 ± 35 | -51 ± 11 | -26 ± 20 | -130 ± 6 | -63 ± 70 | -58 ± 97 | - |
| | glucose | 1 | -82 ± 1 | -225 ± 8 | -60 ± 5 | -29 ± 12 | - | - | -119 ± 7 | - |
| | glucose | 2 | -90 ± 0 | -143 ± 14 | -13 ± 21 | 69 ± 8 | -39 ± 2 | 56 ± 5 | - | - |
| | pyruvate | 1 | -87 ± 1 | -139 ± 16 | -45 ± 4 | -22 ± 58 | -72 ± 7 | -15 ± 39 | 253 | -75 ± 9 |
| | pyruvate | 2 | -88 ± 1 | -152 ± 16 | -25 ± 18 | 19 ± 10 | -63 ± 20 | - | - | -30 ± 50 |
| | succinate | 1 | -85 ± 0 | -159 ± 17 | -18 ± 33 | -3 ± 60 | -71 ± 14 | -56 ± 45 | - | -64 ± 14 |
| | succinate | 2 | -87 ± 0 | -156 ± 11 | -22 ± 18 | 34 ± 25 | -86 ± 5 | -45 ± 7 | - | -56 ± 40 |
| ***E. coli* knockout mutants** | | | | | | | | | | |
| JW1841 | glucose | 1 | -84 ± 2 | -232 ± 9 | -45 ± 10 | -49 ± 8 | - | - | - | - |
| JW3985 | glucose | 1 | -85 ± 1 | -244 ± 11 | -44 ± 6 | -52 ± 7 | - | - | - | - |
| PntAB | glucose | 1 | -74 ± 1 | -196 ± 12 | -53 ± 6 | -20 ± 7 | - | -127 | -147 ± 47 | - |
| UdhA | glucose | 1 | -90 ± 2 | -237 ± 5 | -55 ± 4 | -51 ± 6 | - | - | -216 | - |
| UdhA-PntAB | glucose | 1 | -90 ± 2 | -223 ± 6 | -62 ± 3 | -51 ± 10 | - | -150 ± 13 | -162 ± 28 | - |
| **Growth water experiments** | | | | | | | | | | |
| *B. subtilis* | glucose | 2 | -87 ± 0 | -178 ± 43 | -62 ± 5 | -49 ± 2 | -126 ± 28 | 23 | - | - |
| | | 1 | 194 ± 1 | -25 ± 66 | 67 ± 14 | 82 ± 13 | -14 ± 5 | 9 ± 40 | - | - |
| | | 1 | 448 ± 1 | 80 ± 96 | 138 ± 3 | 217 ± 15 | 103 ± 7 | 177 ± 37 | - | - |
| | | 1 | 618 ± 1 | 116 ± 133 | 204 ± 19 | 228 ± 16 | 191 ± 2 | - | - | - |
| | | 1 | 911 ± 0 | 244 ± 91 | 301 ± 7 | 393 ± 17 | 317 ± 7 | 338 ± 37 | - | - |
| *E. coli* | glucose | 2 | -80 ± 0 | -394 ± 6 | -198 ± 9 | - | - | - | - | - |

| | | | | | | | | | | |
|---|---|---|---|---|---|---|---|---|---|---|
| | | 1 | 128 ± 0 | -272 ± 6 | -90 ± 3 | - | - | - | - | - |
| | | 1 | 337 ± 0 | -141 ± 10 | 15 ± 6 | - | - | - | - | - |
| | | 1 | 545 ± 0 | -21 ± 3 | 94 ± 8 | - | - | - | - | - |
| *E. meliloti* | glucose | 2 | -83 ± 1 | -237 ± 43 | -31 ± 10 | -24 ± 10 | -117 ± 16 | -28 ± 10 | - | - |
| | | 1 | 111 ± 2 | -130 ± 30 | 27 ± 15 | 56 ± 32 | -21 ± 8 | 67 ± 11 | 219 ± 187 | - |
| | | 1 | 248 ± 1 | -50 ± 44 | 100 ± 8 | 127 ± 25 | 71 ± 13 | 150 ± 26 | 361 | - |
| | | 1 | 405 ± 1 | 46 ± 48 | 163 ± 3 | 172 ± 12 | 129 ± 1 | 210 ± 28 | 307 | - |
| | | 1 | 544 ± 1 | 86 ± 74 | 219 ± 34 | 255 ± 12 | 228 ± 5 | 295 ± 44 | - | - |
| *P. fluorescens* | glucose | 2 | -88 ± 1 | -281 ± 39 | -55 ± 20 | -41 ± 3 | -61 ± 20 | 38 ± 20 | - | - |
| | | 1 | 319 ± 1 | -70 ± 47 | 103 ± 49 | 136 ± 11 | 169 ± 8 | 261 ± 32 | - | - |
| | | 1 | 643 ± 1 | 117 ± 75 | 209 ± 45 | 337 ± 51 | 397 ± 9 | 444 ± 2 | - | - |
| | | 1 | 868 ± 1 | 234 ± 204 | 354 ± 36 | 427 ± 9 | 436 ± 24 | - | - | - |
| *R. radiobacter* | glucose | 2 | -90 ± 0 | -143 ± 14 | -13 ± 21 | 69 ± 8 | -39 ± 2 | 56 ± 5 | - | - |
| | | 1 | 100 ± 1 | -73 ± 38 | 68 ± 11 | 143 ± 24 | 33 ± 5 | 81 ± 35 | 103 | - |
| | | 1 | 253 ± 0 | -3 ± 56 | 108 ± 32 | 201 ± 21 | 115 ± 3 | - | - | - |
| | | 1 | 497 ± 0 | 110 ± 82 | 159 ± 11 | 278 ± 10 | 180 ± 4 | 279 ± 12 | 674 ± 3 | - |
| | | 1 | 632 ± 2 | -178 ± 43 | -62 ± 5 | -49 ± 2 | -126 ± 28 | 23 | - | - |

[1]Biological replicates are designated as 1 and 2. Data for the most $^{2}H$-depleted growth water condition for each organism are included as the second glucose replicate within the 'Wildtype organisms' conditions. Note that *E. coli* was cultured on glucose in two different experiments, so these cultures are not biological replicates (see Sections 2.1 and 2.2 in the main text).

[2]$\delta^{2}H_{AA}$ values for which no errors are reported were measured in only one of three analytical replicates.

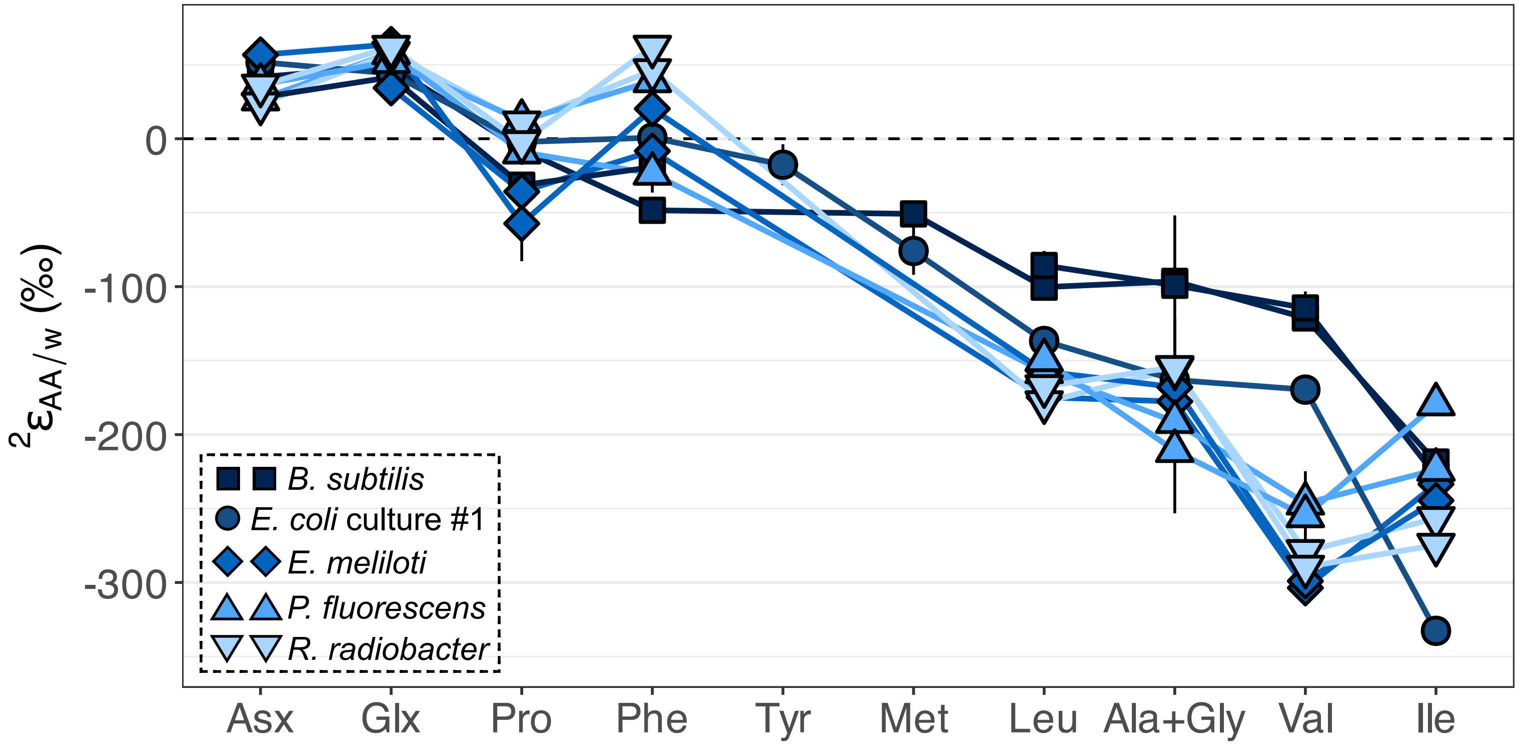


**Figure S6.** Summary of $^2H/^1H$ fractionations between amino acids and water in replicate cultures of wildtype organisms grown on glucose (except for *E. coli*, for which only data from culture #1 is plotted). Error bars indicate the propagated uncertainties (±1σ) from the amino acid, derivative, and water $\delta^2H$ measurements. One $\delta^2H$ value is given for alanine + glycine because these amino acids co-elute on a ZB-5ms column when derivatized as MOC methyl esters (Fig. S1).

## 4. ERROR ASSOCIATED WITH IMPROPER δ$^2$H CORRECTIONS FOR N-BOUND HYDROGEN IN AMINO ACIDS

Amine-, hydroxyl-, and carboxyl-bound hydrogen atoms rapidly exchange with water so do not contribute isotopic information about 'native' (i.e., non-exchangeable, carbon-bound) hydrogen. Derivatization of these functional groups removes all but one exchangeable hydrogen atom, which remains on the amine group and can dilute or obscure native δ$^2$H$_{AA}$ values. Contribution by this remaining N-bound hydrogen to measured δ$^2$H$_{AA}$ values must therefore be corrected (in addition to corrections for exogenous hydrogen from derivative reagents). Here, we calculate possible errors ($\alpha_{error}$) arising from improper correction for the amine hydrogen in two scenarios, with results for a range of δ$^2$H$_{AA}$ values and equilibrium fractionations between NH-containing compounds and water (Bigeleisen, 1965; Englander and Poulsen, 1969) visualized in Fig. S7:

**Scenario 1:** Measured δ$^2$H$_{AA}$ values are corrected for the derivative hydrogen, but not for the amine-bound hydrogen, thus $F_{CH+NH}$ is reported:

$$F_{tot} = X_{CH}F_{CH} + X_{NH}F_{NH} + X_{dH}F_{dH} \tag{S1}$$
$$where\ R_{NH} = R_w \alpha_{NH/w}$$
$$F_{tot} = X_{CH+NH}F_{CH+NH} + X_{dH}F_{dH}$$
$$F_{CH+NH} = (F_{tot} - X_{dH}F_{dH})/(X_{CH+NH})$$
$$\therefore \alpha_{error} = R_{CH+NH}/R_{CH}$$

where $F$ and $X$ are the respective mole fractions of $^2$H in, and hydrogen from, the component in the derivatized amino acid represented by the subscript: C-bound hydrogen (CH); N-bound hydrogen (NH); derivative hydrogen (dH); combined C-bound and N-bound hydrogen (CH+NH); and total hydrogen, including CH, NH, and dH (tot). $R$ is the $^2$H/$^1$H ratio ($R_w$ is that of water), and $\alpha_{NH/w}$ is the equilibrium fractionation between the N-bound hydrogen and water. Conversion between $F$ and $R$ is achieved via $F = R/(1+R)$ and $R = F/(1-F)$. In general, $F_{CH}$ is the parameter to isolate when correcting δ$^2$H$_{AA}$ values because it exclusively encompasses non-exchangeable, carbon-bound hydrogen. However, in Scenario 1, $F_{CH+NH}$ is reported because $F_{NH}$ is not accounted for.

**Scenario 2:** A correction is applied with the erroneous approximation that $F_{NH} = F_w$ (i.e., the isotope composition of the N-bound hydrogen equals that of water):

$$F_{CH.corr} = (F_{tot} - X_{NH}F_w - X_{dH}F_{dH})/X_{CH} \tag{S2}$$
$$\therefore \alpha_{error} = R_{CH.corr}/R_{CH.actual}$$

where $R_{CH.corr}$ and $R_{CH.actual}$ are the erroneously corrected and actual isotope compositions of carbon-bound hydrogen in amino acids, respectively.

As shown in Fig. S7, errors associated with lack of or improper correction for the exchangeable, amine-bound hydrogen may be substantial, even when $F_{NH}$ is approximated as $F_w$. Magnitudes of errors primarily depend on $\alpha_{NH/w}$ and $X_{NH}$ (i.e., amino acids like glycine with the least number of carbon-bound hydrogen atoms are most affected). The difference between $F_{CH}$ and $F_w$ contributes to additional errors in reported $\delta^2H_{AA}$ values, although the size of the effect depends on whether an attempt is made to correct for the amine-bound hydrogen. Notably, $F_{NH}$ is included as a distinct parameter in Eqns. S1 and S2 for illustration of errors; in reality, we cannot isolate $F_{NH}$ without knowledge of $\alpha_{NH/w}$ values for the derivatized forms of each amino acid. In our correction scheme used in this study, we measure $F_{NH} + F_{dH}$ (where dH represents hydrogen from methyl chloroformate, MCF) as a single parameter ($F_{MCF+NH}$; see Section 2.5 in the main text).

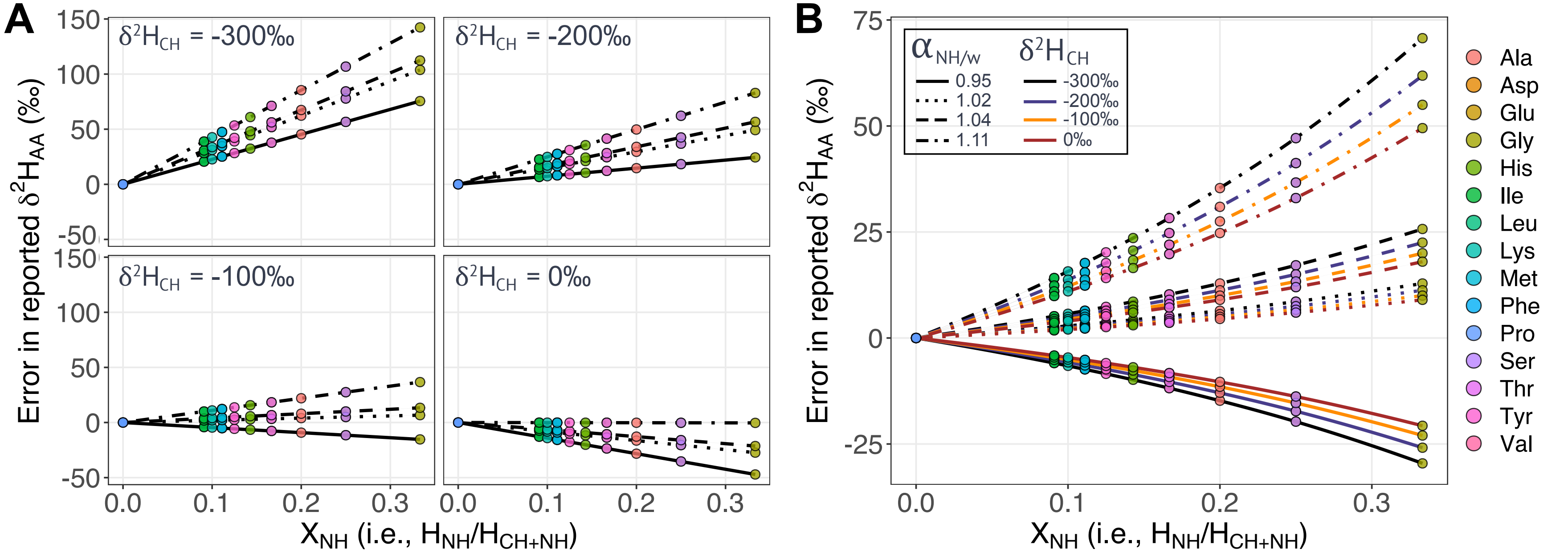


**Figure S7.** Theoretical errors in reported $\delta^2H_{AA}$ values when **(A)** no correction for the isotopic contribution by amine-bound hydrogen ($F_{NH}$) is made (Eqn. S1), or **(B)** when a correction is made with the erroneous assumption that $F_{NH}$ is equivalent to the isotopic composition of water ($F_w$; Eqn. S2). Equilibrium fractionations between amine-bound and water hydrogen ($\alpha_{NH/w}$) have yet to be determined for amino acids, so we use $\alpha_{NH/w}$ values reported in the literature for a variety of NH-containing compounds: 1.02 (acetamide), 1.04 (formamide), and 1.11 (aniline; Bigeleisen, 1965); and 1.095 (peptide group; calculated as ½ the magnitude of $^3H/^1H$ fractionation reported in Englander and Poulsen, 1969). Errors are calculated for a range of representative isotope ratios of carbon-bound hydrogen in amino acids ($\delta^2H_{CH}$ = -300 to 0‰), where $\delta^2H_w$ = -100‰ and $\delta^2H_{dH}$ is assumed to already be subtracted from measured amino acid isotope ratios. Note that only one set of results for amino acids with identical $X_{NH}$ values is displayed.

Proline is the only amino acid in which no amine-bound hydrogen remains after derivatization. In theory, this would allow proline $\delta^2$H values to be reported without correction for exchangeable hydrogen if the isotopic compositions of the derivative reagents are measured independently (hence no errors in proline $\delta^2$H values arise when this correction is not applied in our theoretical calculations; Fig. S7). In our study, some error accompanies reported proline $\delta^2$H values, as we cannot isolate $F_{NH}$ from $F_{MCF+NH}$ in our measurement (see Section 2.5 in the main text). Thus, $\delta^2$H values for proline had to be corrected for the combined value of $F_{MCF+NH}$ using the mass balance equation

$$F_{CH.corr} = (F_{tot} - 0.23F_{MeOH} - 0.23F_{MCF+NH})/0.54 \qquad \text{(S3)}$$
$$\therefore \alpha_{error} = R_{CH.corr}/R_{CH.actual}$$

where $F_{tot}$ is the measured isotopic composition of derivatized proline (i.e., including the derivative MeOH and MCF hydrogen, but not including amine-bound hydrogen). Calculations across a range of representative $\delta^2H_{Pro}$, $\delta^2H_{MCF}$, and $\alpha_{NH/w}$ values demonstrate that errors in reported proline $\delta^2$H values (resulting from this extraneous derivative correction) are likely no more than 20‰ (Fig. S8).

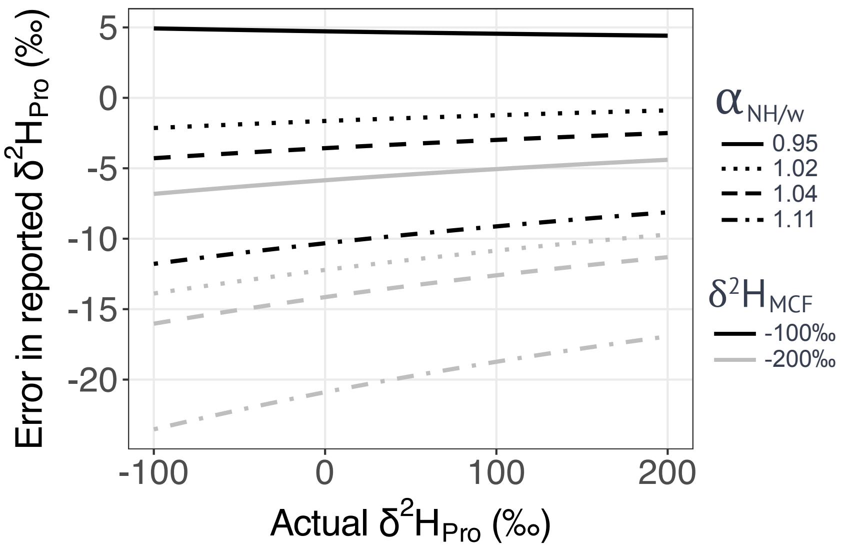


**Figure S8.** Theoretical errors in reported $\delta^2$H values for proline when our derivative correction is applied (see Eqn. S3 and Section 2.5 in the main text), as no amine-bound hydrogen remains in proline after derivatization. Errors are calculated for a range of representative isotopic compositions of proline ('actual' $\delta^2H_{Pro}$ = -100 to +200‰ for carbon-bound hydrogen in proline), where $\delta^2H_{MCF}$ = -150 or -50‰, $\delta^2H_{MeOH}$ and $\delta^2H_w$ = -100‰, and $\alpha_{NH/w}$ values used are those reported in the literature for a variety of NH-containing compounds: 1.02 (acetamide), 1.04 (formamide), and 1.11 (aniline; Bigeleisen, 1965); and 1.095 (peptide group; calculated as ½ the magnitude of $^3H/^1H$ fractionation reported in Englander and Poulsen, 1969).

## 5. HYDROLYSIS AND DERIVATIZATION TESTS

**Table S4.** $\delta^2H$ values and standard deviations (from duplicate or triplicate analyses) of amino acids from bovine serum albumin hydrolyzed under different conditions.

| Amino acid | Replicate | Control[1] | Oxic[2] | 105°C[3] | 20h[4] | 48h[5] |
|---|---|---|---|---|---|---|
| Ala+Gly | 1 | -131 ± 2 | -132 ± 2 | -129 ± 1 | -128 ± 1 | -127 ± 2 |
| Ala+Gly | 2 | -129 ± 2 | -127 ± 0 | -128 ± 2 | -129 ± 2 | -128 ± 1 |
| Asx | 1 | -139 ± 2 | -143 ± 0 | -132 ± 2 | -136 ± 2 | -141 ± 1 |
| Asx | 2 | -136 ± 2 | -138 ± 0 | -131 ± 2 | -133 ± 1 | -141 ± 2 |
| Glx | 1 | -110 ± 3 | -115 ± 5 | -98 ± 2 | -104 ± 3 | -111 ± 2 |
| Glx | 2 | -107 ± 3 | -107 ± 1 | -104 ± 1 | -107 ± 2 | -108 ± 1 |
| Ile | 1 | -235 ± 1 | -235 ± 5 | -240 ± 4 | -238 ± 5 | -237 ± 9 |
| Ile | 2 | -233 ± 3 | -232 ± 4 | -235 ± 4 | -235 ± 0 | -238 ± 10 |
| Leu | 1 | -182 ± 1 | -180 ± 3 | -179 ± 0 | -181 ± 1 | -179 ± 3 |
| Leu | 2 | -181 ± 1 | -179 ± 0 | -180 ± 1 | -180 ± 1 | -180 ± 1 |
| Lys | 1 | -167 ± 4 | -171 ± 0 | -165 ± 0 | -170 ± 5 | -171 ± 3 |
| Lys | 2 | -166 ± 2 | -167 ± 1 | -168 ± 2 | -171 ± 4 | -170 ± 4 |
| Met | 1 | -133 ± 11 | -145 ± 11 | -139 ± 11 | -144 ± 10 | -136 ± 8 |
| Met | 2 | -140 ± 13 | -136 ± 0 | -148 ± 10 | -134 ± 9 | -141 ± 8 |
| Phe | 1 | -134 ± 2 | -132 ± 1 | -129 ± 1 | -130 ± 1 | -130 ± 1 |
| Phe | 2 | -131 ± 2 | -130 ± 0 | -131 ± 1 | -129 ± 0 | -129 ± 2 |
| Pro | 1 | 16 ± 2 | 4 ± 2 | 12 ± 2 | 11 ± 1 | 6 ± 3 |
| Pro | 2 | 12 ± 2 | 2 ± 1 | 7 ± 1 | 8 ± 1 | 6 ± 2 |
| Tyr | 1 | -131 ± 3 | -130 ± 1 | -124 ± 4 | -128 ± 1 | -126 ± 2 |
| Tyr | 2 | -127 ± 4 | -126 ± 1 | -127 ± 2 | -129 ± 2 | -127 ± 1 |
| Val | 1 | -165 ± 2 | -164 ± 4 | -160 ± 2 | -162 ± 4 | -163 ± 2 |
| Val | 2 | -163 ± 3 | -159 ± 2 | -159 ± 3 | -160 ± 2 | -164 ± 1 |

[1]Anoxic hydrolysis in 6N HCl for 24h at 110°C.

[2-5]Identical conditions as used in control, except for one variable altered in each treatment:

[2]Samples were not sparged with $N_2$ prior to sealing sample vials for hydrolysis (i.e., $O_2$ was present during hydrolysis).

[3]Samples were hydrolyzed at 105°C.

[4]Samples were hydrolyzed for 20h.
[5]Samples were hydrolyzed for 48h.

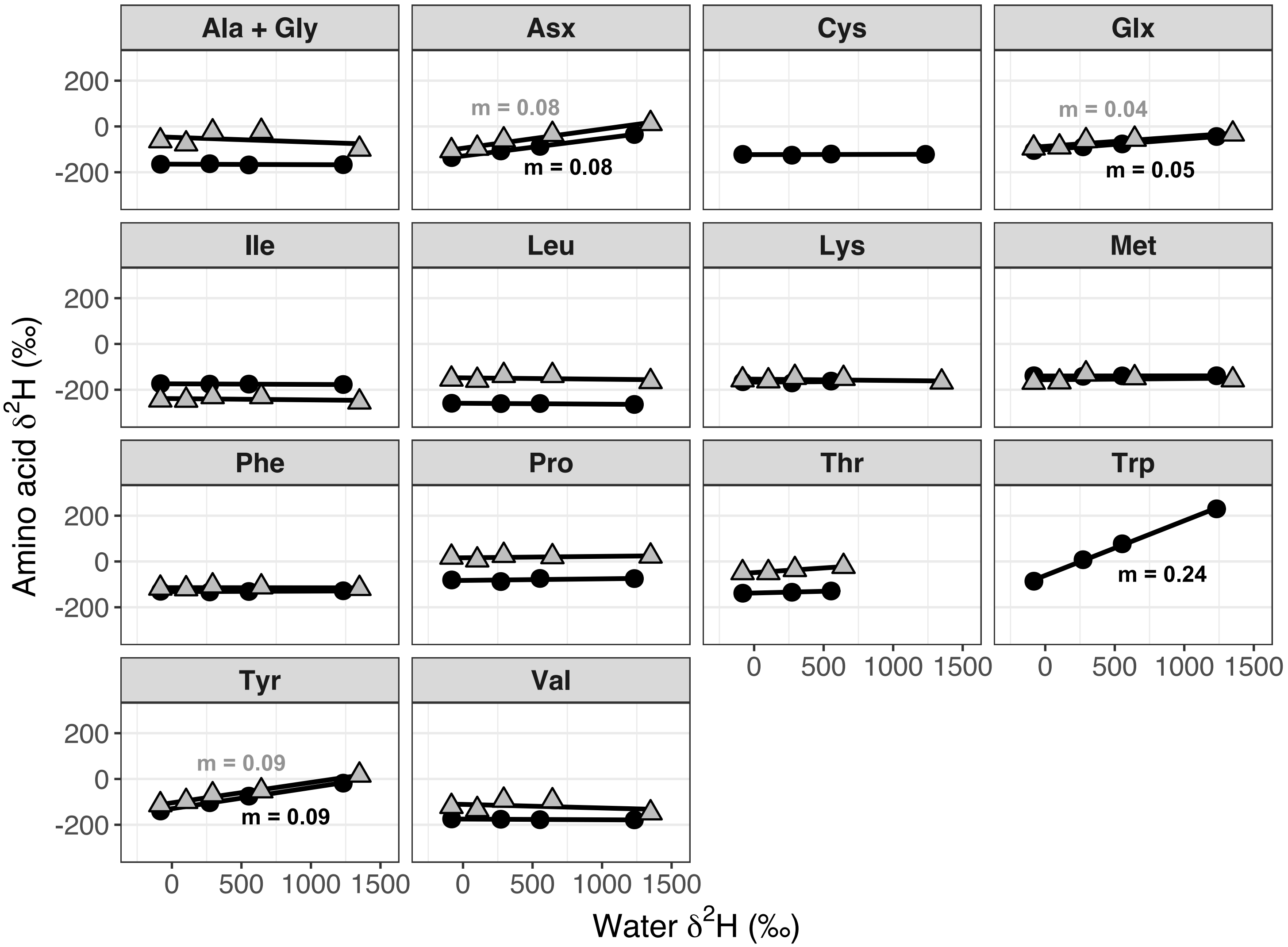


**Figure S9.** Effect of hydrolysis on amino acid $\delta^2$H values from BSA (gray triangles) and a mixture of pure standards (black circles). Amino acids were hydrolyzed in 6N HCl at 110°C for 24 h. The acid was prepared with different $\delta^2$H values to test for hydrogen exchange during hydrolysis. Slopes (m) ≥0.02 are annotated in gray for BSA and black for the standard mixture.

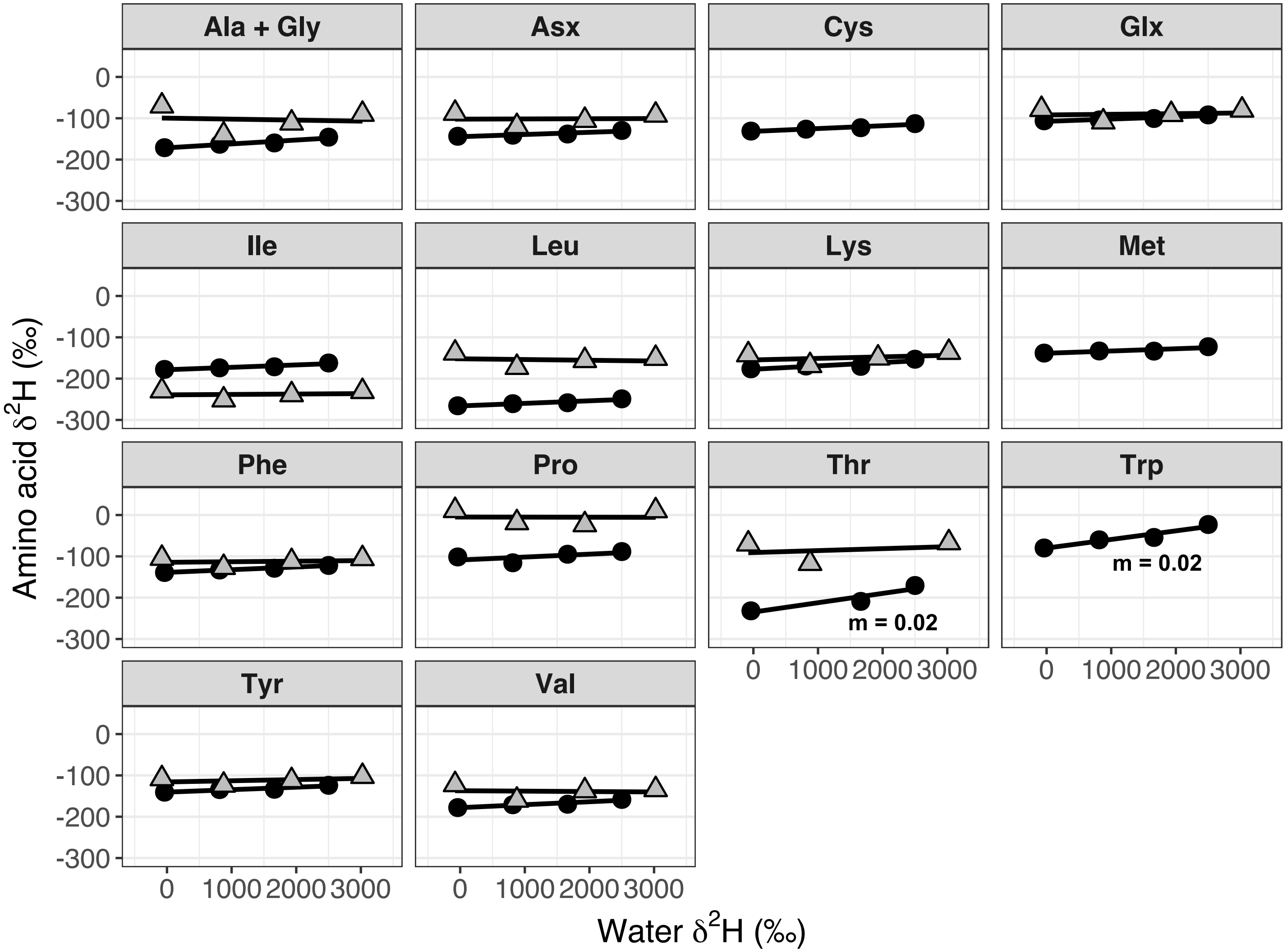


**Figure S10.** Effect of derivatization on amino acid $\delta^2$H values from BSA (gray triangles) and a mixture of pure standards (black circles). Amino acids were derivatized to MOC esters with 7:2:3 v/v/v anhydrous MeOH, pyridine, and MCF in 0.1N HCl at room temperature. The solvent was prepared with different $\delta^2$H values to test for hydrogen exchange during derivatization. Slopes (m) ≥0.02 are annotated in gray for BSA and black for the standard mixture.

## 6. CONTROLS ON VARIATIONS IN $^2\varepsilon_{AA/w}$ VALUES

### 6.1 Water fraction factors

Hydrogen in amino acids ultimately originates from two external sources: organic substrates and water. The hydrogen isotope composition of each amino acid can therefore be conceptualized as the weighted contributions of these sources according to the mass balance equation

$$R_{AA} = X_w \alpha_{AA/w} R_w + (1 - X_w)\, \alpha_{AA/s} R_s \tag{S4}$$

where $R_{AA}$, $R_w$, and $R_s$ denote the non-exchangeable hydrogen isotope ratios of the amino acid, water, and substrate, respectively; $X_w$ is the mole fraction of water-derived hydrogen in an amino acid; and $\alpha_{AA/w}$ and $\alpha_{AA/s}$ are the net isotopic fractionations associated with hydrogen uptake and incorporation from water and the substrate, respectively (although note that treatment of $\alpha_{AA/w}$ and $\alpha_{AA/s}$ as distinct is often an artificial simplification – see Zhang et al., 2009). Without additional constraints on the system, a unique solution for the three unknown parameters ($X_w$, $\alpha_{AA/w}$, and $\alpha_{AA/s}$) is not possible (Sessions and Hayes, 2005). However, the combined effect of $X_w \cdot \alpha_{AA/w}$ (termed the 'water fraction factor' after Kopf, 2014) can be obtained from the slope of the regression between $R_{AA}$ and $R_w$, with values resulting from culturing experiments in which the $\delta^2$H value of an organism's growth water is manipulated (Fig. S11A). Water fraction factors provide an additional means to compare physiological differences across organisms, and when combined with $^2\varepsilon_{AA/w}$ data, can help constrain some of the control by $X_w$ versus $\alpha_{AA/w}$ on $^2\varepsilon_{AA/w}$ values.

Wildtype organisms showed similar patterns of water fraction factors (Figs. S11B; Table S5), implying similar net amino acid/water fractionations for a given amino acid. Water fraction factors in *E. coli* are comparable to those calculated from similar published growth water experiments (Table S5; Fogel et al., 2016), demonstrating that organisms yield reproducible amino acid/water fractionations when grown under similar conditions. For a given amino acid in *B. subtilis, E. coli,* and *R. radiobacter*, water fraction factors are approximately within error, suggesting that differences in corresponding $\delta^2H_{AA}$ values in these organisms are likely not driven by differences in $\alpha_{AA/w}$ or $X_w$, but rather may be attributed to variations in the isotopic composition of hydrogen from organic precursors or in the isotope fractionations imparted during post-synthesis reactions. In contrast, the generally higher but similar ordering of water fraction factors in *E. meliloti* and *P. fluorescens* imply potential differences in $X_w$ at the level of central metabolites (e.g., pyruvate), which in turn may reflect the variations in relative fluxes through central metabolic pathways, as well as different extents of organic hydrogen equilibration with water in pools of central metabolites. Some of the variation across organisms may be driven by different intracellular water isotope ratios, which can be distinct from the growth medium when cellular growth rates are high (Kreuzer-Martin et al., 2006), although the extent to which this effect was relevant in our cultures is unclear.

The overall pattern of water fraction factors generally followed that of ${}^{2}\varepsilon_{AA/w}$ values (Fig. 4A), with the exception of phenylalanine, which had notably low water fraction factors yet high $\delta^2$H values. This difference is puzzling given that the small phenylalanine/water fractionations in each substrate condition (Fig. 6) support a high $X_w$ and large $\alpha_{AA/w}$ (i.e., small net fractionation), yet together these would yield high water fraction factors. This discrepancy could not be resolved here. Proline had the highest water fraction factors, which, coupled with its high $\delta^2$H values and potentially high fraction of water-derived hydrogen (Fig. S16), can likely be explained by both a large $X_w$ and large $\alpha_{AA/w}$ (i.e., small fractionation). In turn, a large $\alpha_{AA/w}$ can be explained in part by ${}^{2}$H-enrichment of water-derived hydrogen from acetyl-CoA by citrate synthase (Section 4.1.1 in main text), which would increase the apparent $\alpha_{AA/w}$ value toward or beyond unity. The ordering of water fraction factors for leucine, isoleucine, and valine is consistent across organisms and matches that of ${}^{2}\varepsilon_{AA/w}$values in *E. meliloti, P. fluorescens,* and *R. radiobacter*, suggesting a strong control by the water-derived fraction of hydrogen in these amino acids on their overall isotopic compositions.

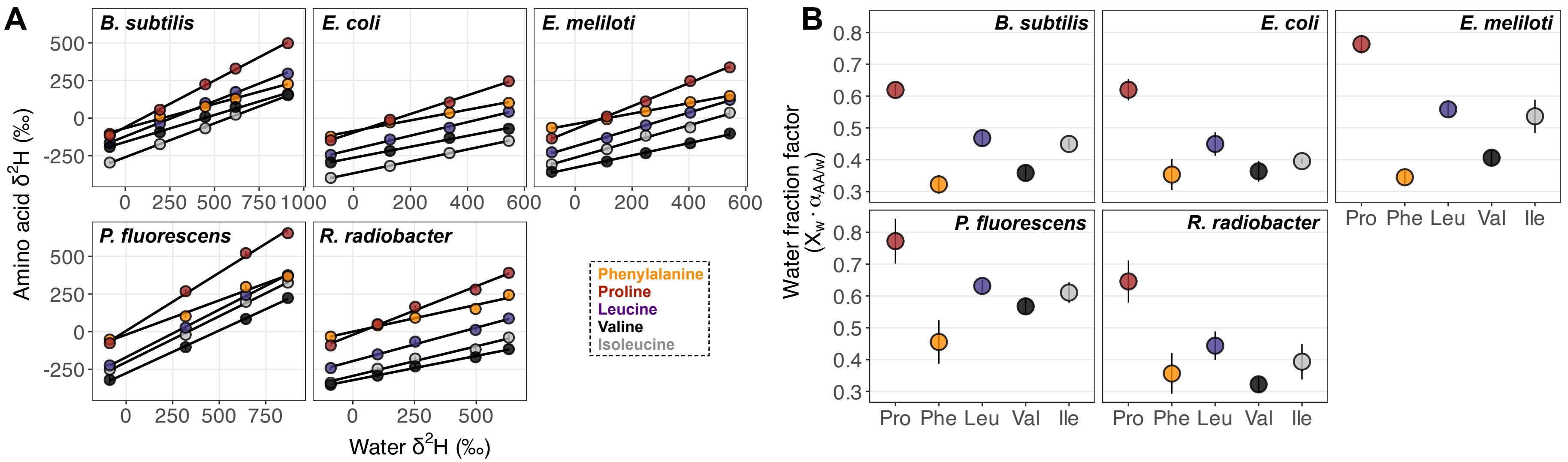


**Figure S11.** Influence of media $\delta^2$H on amino acid $\delta^2$H values in wildtype organisms grown on glucose. **(A)** Regressions of $\delta^2$H values of amino acids versus media water. Error bars indicate the propagated uncertainties ($\pm 1\sigma$) from the amino acid, derivative, and water measurements, and are smaller than symbols. **(B)** Summary of water fraction factors (i.e., slopes from regressions in **A**). Error bars are 95% confidence intervals of the coefficients from the linear regression fit. Slopes and 95% confidence intervals are summarized in Table S5. Note that the *E. coli* cultures were grown at a different time, and amino acids were derivatized following a different reaction procedure, compared to the cultures of the other four organisms (see Sections 2.1 and 2.2 in the main text). We do not expect these differences to affect the interpretations drawn from this data.

**Table S5.** Summary of slopes and 95% confidence intervals calculated from regressions of amino acid versus water $\delta^2H$ values for wildtype organisms grown on glucose (Fig. S11).

| Organism | Study | Proline | Phenylalanine | Leucine | Valine | Isoleucine |
|---|---|---|---|---|---|---|
| *B. subtilis* | This study | 0.62 ± 0.018 | 0.32 ± 0.030 | 0.47 ± 0.025 | 0.36 ± 0.024 | 0.45 ± 0.017 |
| *E. coli* | This study | 0.62 ± 0.017 | 0.35 ± 0.025 | 0.45 ± 0.019 | 0.36 ± 0.017 | 0.40 ± 0.004 |
| *E. coli* | Fogel et al., 2016 | 0.56 ± 0.025 | 0.24 ± 0.077 | 0.44 ± 0.009 | 0.37 ± 0.023 | 0.45 ± 0.009 |
| *E. meliloti* | This study | 0.76 ± 0.029 | 0.34 ± 0.022 | 0.56 ± 0.026 | 0.41 ± 0.025 | 0.54 ± 0.052 |
| *P. fluorescens* | This study | 0.77 ± 0.071 | 0.46 ± 0.068 | 0.63 ± 0.020 | 0.57 ± 0.024 | 0.61 ± 0.032 |
| *R. radiobacter* | This study | 0.65 ± 0.066 | 0.36 ± 0.064 | 0.44 ± 0.045 | 0.32 ± 0.026 | 0.39 ± 0.056 |

## 6.2 Pyruvate hydrogen

Pyruvate occupies a crucial node in central metabolism and contributes hydrogen to all amino acids either directly (the case for leucine, valine, and isoleucine), or indirectly (through α-ketoglutarate for proline when pyruvate hydrogen is routed through the TCA cycle, and through PEP for phenylalanine when flux is gluconeogenic). The altered metabolic programming in organisms grown on different substrates undoubtedly alters the hydrogen isotope composition of pyruvate, which likely contributes to some of the observed shifts in all $\delta^2H_{AA}$ values. To explore this control, we estimated the fraction of 'pyruvate-related' hydrogen in each amino acid (i.e., the fraction of hydrogen directly derived from pyruvate or derived from a metabolite whose hydrogen exchanges with that of pyruvate). For leucine, valine, and isoleucine, pyruvate hydrogen is directly incorporated (Figs. 1 and S18-S19), thus pyruvate-related hydrogen fractions are 60, 75, and 30%, respectively in these amino acids. Pyruvate hydrogen is routed into proline via acetyl-CoA during pyruvate and succinate metabolism (Gerosa et al., 2015); estimates of 29% pyruvate hydrogen in proline are included for these conditions based on hydrogen accounting (Figs. 1 and S16). Upon growth on acetate, pyruvate hydrogen is not technically routed into proline (e.g., Gerosa et al., 2015; Dolan et al., 2020); nevertheless, substantial shifts in the $\delta^2H$ value of acetyl-CoA from glucose to acetate metabolism likely occurred, as evidenced by proline's significantly higher $\delta^2H$ values. Thus, the fraction of acetyl-CoA-derived hydrogen in proline during acetate metabolism (29%) is additionally included in the pyruvate-related fraction of proline hydrogen. PEP hydrogen, which is incorporated into phenylalanine, is directly related to pyruvate hydrogen through pyruvate kinase or PEP synthetase activity (which interconvert PEP and pyruvate). However, this relationship is impeded when PEP is predominantly synthesized through PEP carboxykinase, or pyruvate through KDPG aldolase (Fig. S3), which occurs when different central metabolic pathways are activated (Gerosa et al., 2015; Dolan et al., 2020). Because exact determination of the relationship between PEP and pyruvate hydrogen in each substrate growth condition is difficult, an estimate of 50% relatedness was used here, which equates to ~19% pyruvate-related hydrogen in phenylalanine when accounting for the fact that 38% of hydrogen in phenylalanine is PEP-derived. Plotting these pyruvate-related hydrogen fractions against shifts in $\delta^2H_{AA}$ values in wildtype organisms grown on glucose versus on acetate, pyruvate, or succinate reveals that changes in the isotope composition of pyruvate-related hydrogen alone explains 15–52% of the variation in $\delta^2H_{AA}$ values (Fig. S12).

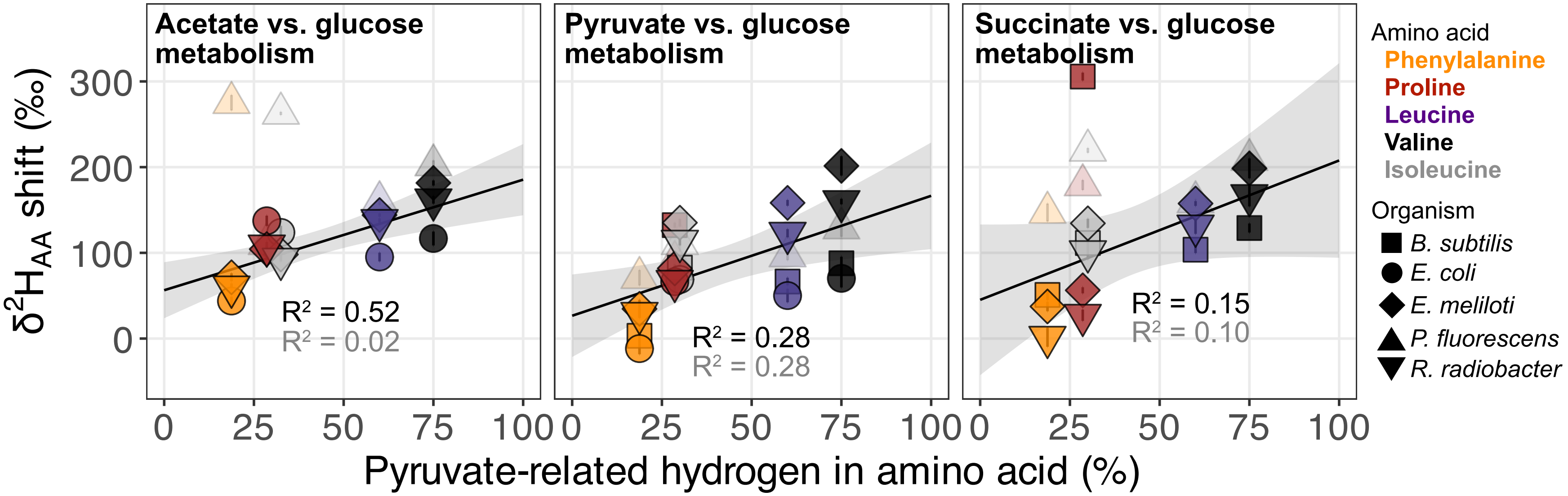


**Figure S12.** Regressions of pyruvate-derived hydrogen in amino acids versus shifts in $\delta^2H_{AA}$ values in wildtype organisms grown on glucose versus on acetate, pyruvate, and succinate. $\delta^2H_{AA}$ shifts were calculated as the difference in $\delta^2H$ value of a given amino acid between the two conditions compared in each panel. Error bars on individual data points indicate the propagated uncertainties ($\pm 1\sigma$) from each pair of $\delta^2H_{AA}$ values measured and are smaller than symbols. Data are shown for one biological replicate. The shaded gray region indicates the 95% confidence interval of the coefficients from the linear regression fit. Adjusted $R^2$ values for regressions are shown when *P. fluorescens* data (transparent symbols) are excluded (black) versus included (gray).

### 6.3 NADPH hydrogen

For amino acids that derive hydrogen from NADPH, regression analysis shows that 61% of $\delta^2H_{AA}$ shifts from glucose to acetate metabolism, and 34% of shifts from glucose to pyruvate metabolism, may be explained when only considering the proportion of NADPH-derived hydrogen in each amino acid (Fig. S13). Significant deviations in proline $\delta^2H$ values in organisms grown on succinate impedes interpretation of NADPH's influence on $\delta^2H_{AA}$ values during succinate metabolism. As the isotopic compositions of leucine and valine are not impacted by NADPH but vary significantly across substrate conditions, contributions by pyruvate must be additionally accounted for to capture the major variations in all amino acid $\delta^2H$ values (Fig. 7B; Supplementary Section 6.2).

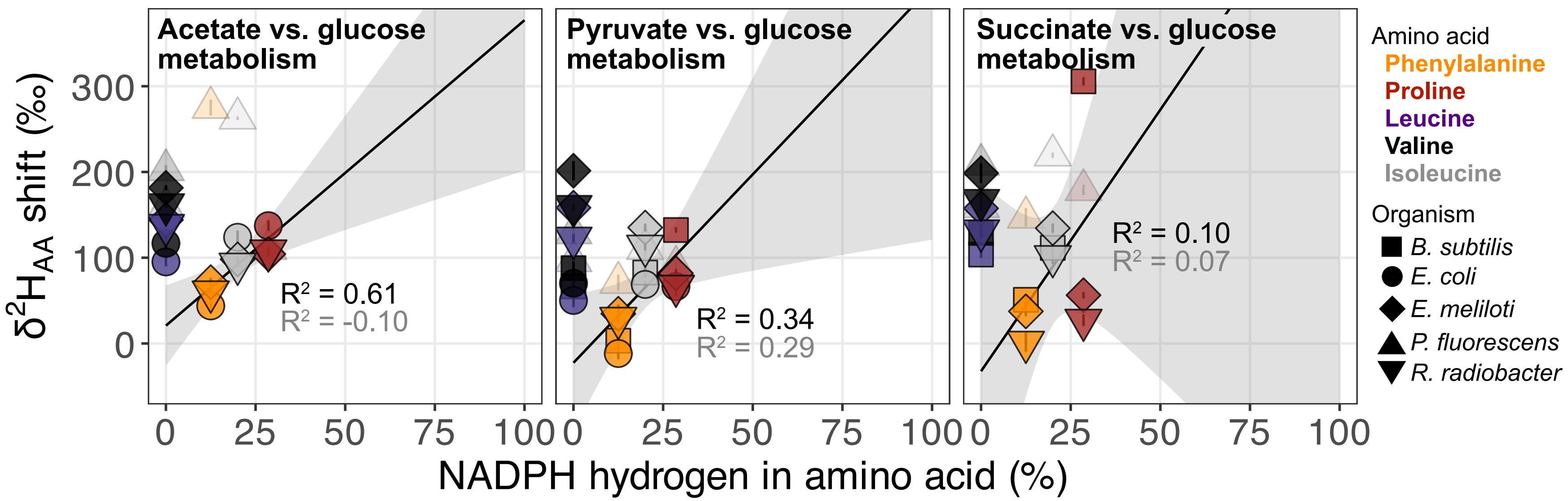


**Figure S13.** Regressions of NADPH-derived hydrogen in amino acids versus shifts in $\delta^2H_{AA}$ values in wildtype organisms grown on glucose versus on acetate, pyruvate, and succinate. $\delta^2H_{AA}$ shifts were calculated as the difference in $\delta^2H$ value of a given amino acid between the two conditions compared in each panel. Error bars on individual data points indicate the propagated uncertainties (±1σ) from each pair of $\delta^2H_{AA}$ values measured and are smaller than symbols. Data are shown for one biological replicate. The shaded gray region indicates the 95% confidence interval of the coefficients from the linear regression fit. Adjusted $R^2$ values for regressions are shown when *P. fluorescens* data (transparent symbols) are excluded (black) versus included (gray). Regressions displayed only include amino acids that inherit hydrogen from NADPH (i.e., phenylalanine, proline, and isoleucine); when all amino acids are included, adjusted $R^2$ values are ~0 with or without *P. fluorescens* data.

Variations in $\delta^2H_{AA}$ values due to NADPH may be further interrogated through $\delta^2H$ shifts in proline, whose hydrogen sources and proportions (29% from NAD(P)H, 29% from acetyl-CoA, and 42% from water; Figs. 1 and S16) are invariant regardless of the catabolic pathway activated. Upon growth on different substrates relative to growth on glucose, changes in the isotopic composition of proline beyond those due to shifts in acetyl-CoA $\delta^2H$ are presumably related to changes in the $\delta^2H$ value of NADPH (thus are referred to here as "NADPH-driven $^2H$-enrichment," or $\Delta^2H_{NADPH}$), as the water-derived fraction is assumed to be isotopically invariant. We can estimate $\Delta^2H_{NADPH}$ of proline across growth conditions by subtracting the relative contribution of acetyl-CoA $\delta^2H$ variations ($\Delta^2H_{AcCoA}$) from total shifts in proline $\delta^2H$ values ($\Delta^2H_{Pro}$):

$$\Delta^2H_{NADPH} = 7\Delta^2H_{Pro}/2 - \Delta^2H_{AcCoA} \quad \text{(S5)}$$

In turn, $\Delta^2 H_{AcCoA}$ can be estimated based on assumptions about how hydrogen is routed through the catabolic pathways. During glucose, pyruvate, and succinate metabolism, the majority of acetyl-CoA is produced from pyruvate via pyruvate dehydrogenase (Gerosa et al., 2015). The methyl group of pyruvate remains intact during this conversion, so the hydrogen isotope composition of the acetyl-CoA methyl group is presumed to be identical to that of cellular pyruvate (Fig. 8). Thus, $\Delta^2 H_{AcCoA}$ from glucose to pyruvate or succinate metabolism can be determined based on shifts in cellular pyruvate $\delta^2$H ($\Delta^2 H_{Pyr}$; Eqn. S6), which, in turn, can be calculated through shifts in leucine or valine $\delta^2$H ($\Delta^2 H_{Leu}$ or $\Delta^2 H_{Val}$), as leucine and valine only inherit hydrogen from pyruvate and water (Eqn. S7; Figs. 1 and S18; Table S6), and the water-derived fraction is presumed to be isotopically invariant.

$$\Delta^2 H_{AcCoA} = \Delta^2 H_{Pyr} \tag{S6}$$

$$\Delta^2 H_{Pyr} = 10\Delta^2 H_{Leu}/6 \tag{S7}$$
$$\Delta^2 H_{Pyr} = 8\Delta^2 H_{Val}/6$$

During acetate metabolism, pyruvate hydrogen does not route into proline (Fig. 8; Gerosa et al., 2015; Dolan et al., 2020), so acetyl-CoA $\delta^2$H shifts could not be directly calculated through $\Delta^2 H_{Leu}$ or $\Delta^2 H_{Val}$. Instead, cellular acetyl-CoA $\delta^2$H values during acetate and glucose metabolism were estimated individually. The hydrogen isotope composition of acetyl-CoA produced during acetate metabolism ($\delta^2 H_{AcCoA.Ace}$) was approximated as equal to that of the acetate substrate (Eqn. S8; $\delta^2 H_{Ace}$ = -76 ± 20 ‰, measured by Zhang et al., 2009). The isotopic composition of acetyl-CoA produced during glucose metabolism ($\delta^2 H_{AcCoA.Glu}$) was assumed to be equal to that of intracellular pyruvate ($\delta^2 H_{Pyr.Glu}$; Eqn. S9; Fig. 8). In turn, $\delta^2 H_{Pyr.Glu}$ was estimated based on intracellular pyruvate $\delta^2$H shifts from glucose to pyruvate metabolism (Eqn. S10), which were calculated through shifts in leucine and valine $\delta^2$H values (Eqn. S7), with the assumption that during pyruvate metabolism, the isotopic composition of intracellular pyruvate ($\delta^2 H_{Pyr.Pyr}$) is equal to that of the substrate pyruvate (Eqn. S11; where $\delta^2 H_{Pyr}$ = -12 ± 20‰, as measured by Zhang et al., 2009). Estimated shifts in the hydrogen isotope composition of intracellular acetyl-CoA from glucose to acetate metabolism were subtracted from measured shifts in proline $\delta^2$H values in order to estimate the contribution of NADPH to $^2$H-enrichment of proline (Eqn. S5). Results of these calculations are shown in Fig. 7C and Table S6, and are discussed in Section 4.2.1.2 in the main text.

$$\delta^2 H_{AcCoA.Ace} = \delta^2 H_{Ace} \tag{S8}$$

$$\delta^2 H_{AcCoA.Glu} = \delta^2 H_{Pyr.Glu} \tag{S9}$$

$$\delta^2 H_{Pyr.Glu} = \delta^2 H_{Pyr} - (\delta^2 H_{Pyr.Pyr} - \delta^2 H_{Pyr.Glu}) \tag{S10}$$

$$\delta^2 H_{Pyr.Pyr} = \delta^2 H_{Pyr} \tag{S11}$$

**Table S6.** Estimated isotopic compositions of and shifts in intracellular metabolite pools presented in Supplementary Sections 6.2 and 6.3.

| Organism | Condition 2[1] | Pyruvate $\delta^2H$ shift ($\Delta^2H_{Pyr}$)[2] | Intracellular pyruvate $\delta^2H$ in glucose condt.[3] | NADPH-driven $^2H$-enrichment in proline ($\Delta^2H_{NADPH}$)[4] |
|---|---|---|---|---|
| *B. subtilis* | pyruvate | 114 ± 6 | -126 ± 15 | 99 ± 7 |
| | succinate | 172 ± 5 | | 256 ± 7 |
| *E. coli* | acetate | 157 ± 7 | -101 ± 17 | 130 ± 27 |
| | pyruvate | 87 ± 10 | | 41 ± 15 |
| *E. meliloti* | acetate | 241 ± 3 | -278 ± 16 | 46 ± 26 |
| | fructose | 48 ± 13 | | -19 ± 16 |
| | pyruvate | 266 ± 8 | | 5 ± 9 |
| | succinate | 264 ± 8 | | -20 ± 9 |
| *P. fluorescens* | acetate | 268 ± 5 | -178 ± 16 | 81 ± 26 |
| | citrate | 236 ± 9 | | |
| | fructose | 61 ± 5 | | 8 ± 9 |
| | pyruvate | 166 ± 8 | | 42 ± 10 |
| | succinate | 272 ± 8 | | 100 ± 10 |
| *R. radiobacter* | acetate | 223 ± 4 | -221 ± 15 | 66 ± 25 |
| | fructose | 20 ± 10 | | -14 ± 15 |
| | pyruvate | 209 ± 5 | | 6 ± 7 |
| | succinate | 221 ± 11 | | -37 ± 13 |

[1]Condition 1 = glucose metabolism.

[2]Estimated average pyruvate $\delta^2H$ shift from glucose metabolism to growth condition 2, computed from two independent estimates calculated using $\delta^2H$ shifts in leucine and valine (Eqn. S7) in replicate #1 cultures, with $\Delta^2H_{Leu}$ and $\Delta^2H_{Val}$ adjusted for differences in growth medium $\delta^2H$. Errors indicate propagated uncertainties (±1σ) in leucine, valine, and growth medium $\delta^2H$ values in each pair of conditions.

[3]$\delta^2H$ value of intracellular pyruvate produced during glucose metabolism, estimated for each organism as -12‰ – [pyruvate $\delta^2H$ shift from the glucose to pyruvate condition; Eqn. S10]. This calculation assumes that during pyruvate metabolism, the $\delta^2H$ value of intracellular pyruvate is equivalent to that of the substrate pyruvate (-12‰; Zhang et al., 2009). Errors indicate propagated uncertainties (±1σ) in the measured pyruvate $\delta^2H$ value (~20‰; Zhang et al., 2009) and in the estimated pyruvate $\delta^2H$ shifts (see footnote 2).

[4]Estimated $^2H$-enrichment in proline caused by NADPH. See Supplementary Section 6.3 for calculation details. Errors indicate propagated uncertainties (±1σ) in measured proline, pyruvate, and/or acetate $\delta^2H$ values, as well as estimated pyruvate $\delta^2H$ shifts (see footnote 2).

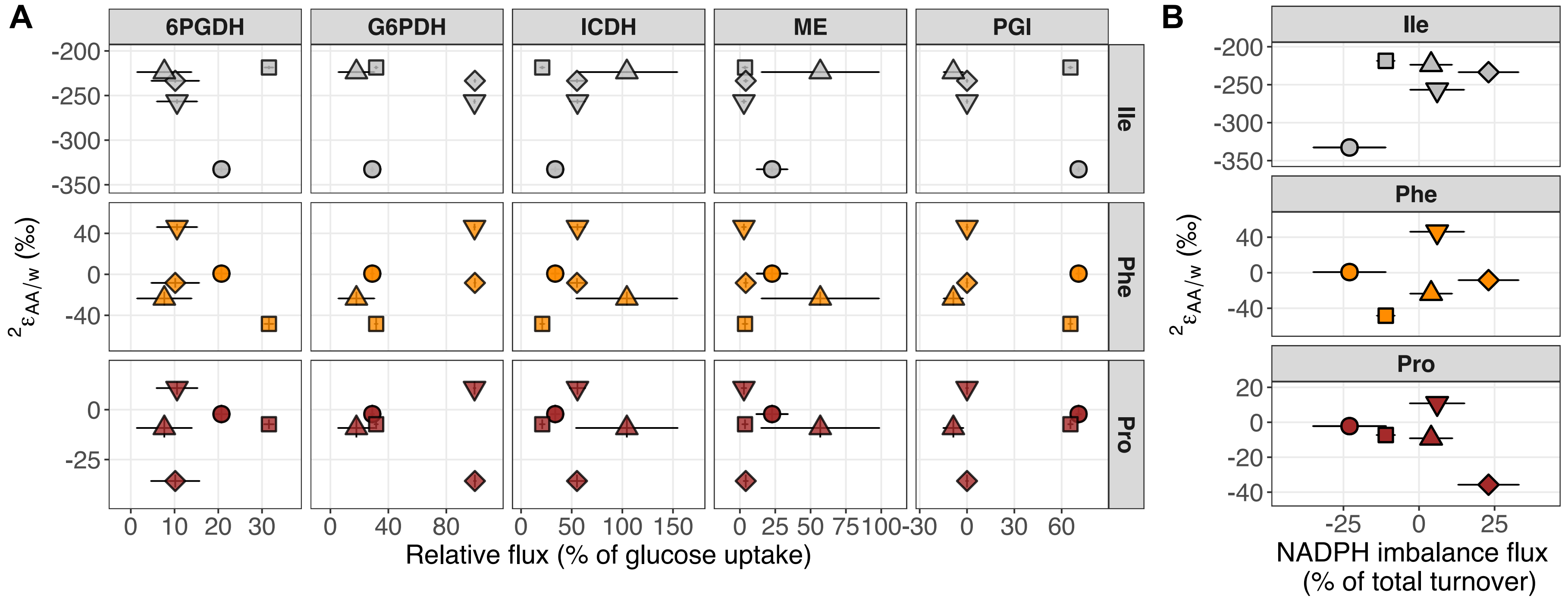


**Figure S14.** Lack of correlations between amino acid/water fractionations and NADPH metabolism in wildtype organisms grown on glucose. **(A)** $^{2}\varepsilon_{AA/w}$ values for amino acids that inherit NADPH hydrogen versus relative carbon flux (i.e., normalized to glucose uptake rates) through NADPH-relevant enzymes. **(B)** $^{2}\varepsilon_{AA/w}$ values versus NADPH imbalance flux, calculated for these organisms in Wijker et al. (2019) as the difference between all NADPH-producing and -consuming fluxes. Error bars represent ±1σ. Only data from replicate #1 cultures are plotted. Enzyme abbreviations: 6PGDH, 6-phosphogluconate dehydrogenase; G6PDH, glucose-6-phosphate dehydrogenase; ICDH, isocitrate dehydrogenase; ME, malic enzyme; PGI, phosphoglucose isomerase.

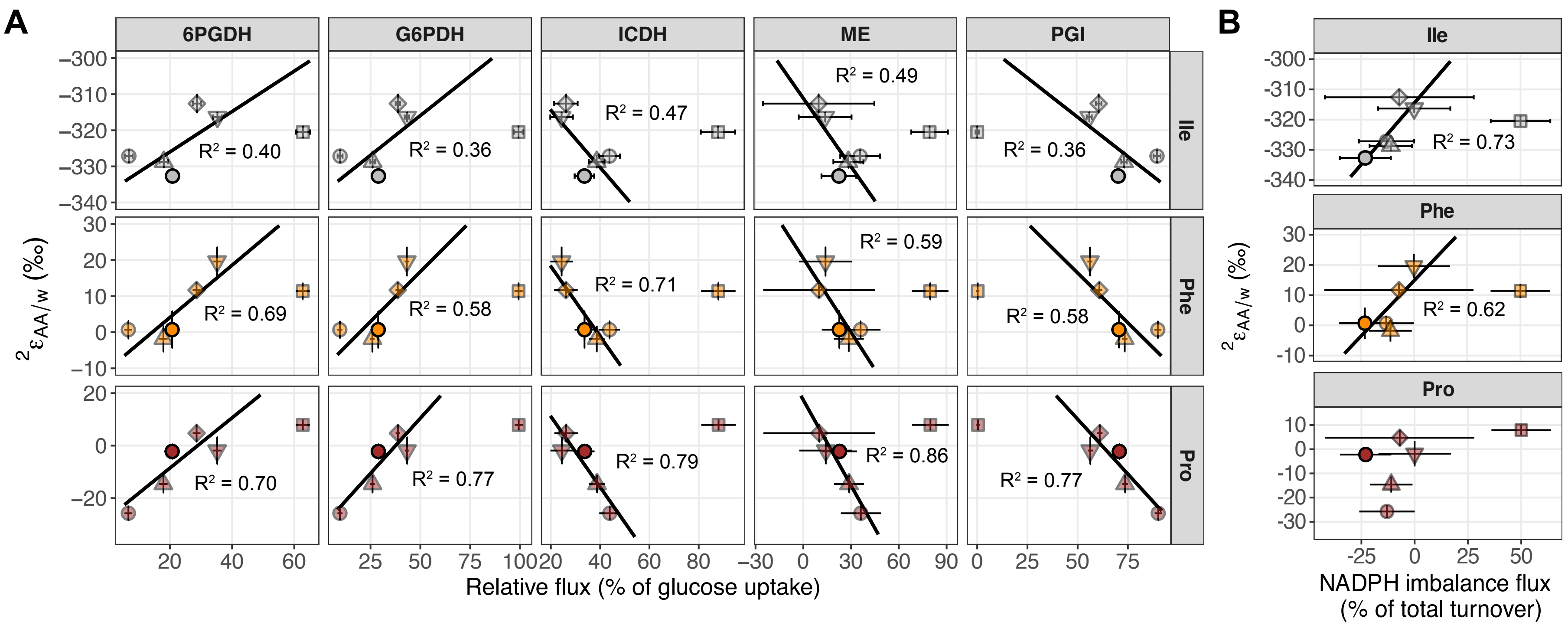


**Figure S15.** Correlations between amino acid/water fractionations and NADPH metabolism in *E. coli* wildtype and mutant organisms grown on glucose. **(A)** $^2\varepsilon_{AA/w}$ values for amino acids that inherit NADPH hydrogen versus relative carbon flux (i.e., normalized to glucose uptake rates) through NADPH-relevant enzymes. **(B)** $^2\varepsilon_{AA/w}$ values versus NADPH imbalance flux, calculated for these organisms in Wijker et al. (2019) as the difference between all NADPH-producing and -consuming fluxes. JW3985 was excluded from all regression analyses because in most cases it was a significant outlier for unclear reasons. Moderate to weak correlations were observed for all comparisons, except for proline $\delta^2$H versus NADPH imbalance flux, which for unknown reasons did not appear to correlate. Error bars represent ±1σ. Enzyme abbreviations: 6PGDH, 6-phosphogluconate dehydrogenase; G6PDH, glucose-6-phosphate dehydrogenase; ICDH, isocitrate dehydrogenase; ME, malic enzyme; PGI, phosphoglucose isomerase.

# 7. BIOSYNTHETIC PATHWAYS

## 7.1 Proline biosynthesis

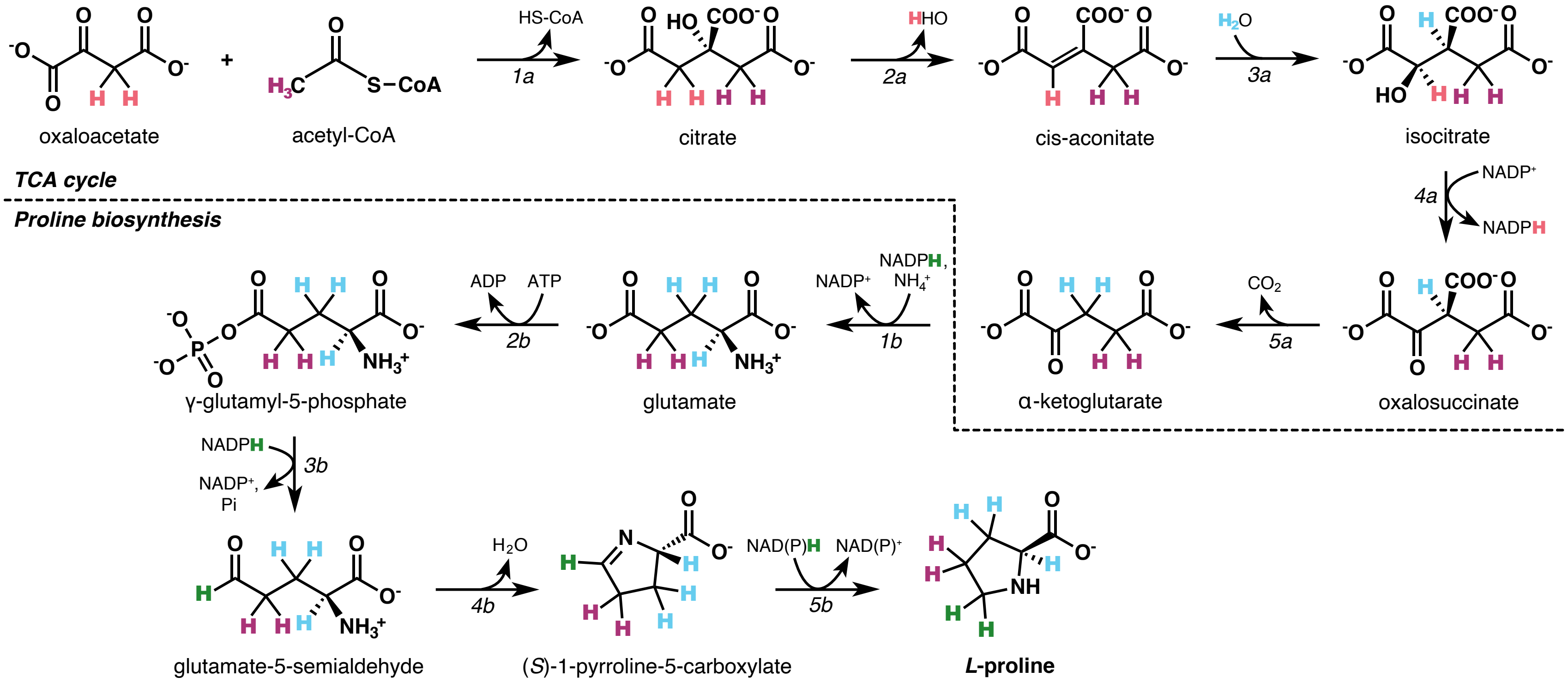


**Figure S16.** General biosynthetic pathway of proline with colors representing tracked hydrogen sources (red for OAA, purple for acetyl-CoA, light blue for water, green for NADPH). As summarized in Section 4.1.1 in the main text, aconitase and isocitrate dehydrogenase stereospecifically remove the oxaloacetate-derived hydrogen from citrate and isocitrate, respectively (Lowenstein, 1967; Smith and York, 1970; Csonka and Fraenkel, 1977; Ochs and Talele, 2020), resulting in exclusive retention of acetyl-CoA hydrogen in proline. The hydrogen atom transferred from NADPH in step 1b likely equilibrates with water due to PLP-dependent enzyme reactions that target the α-hydrogen position in amino acids (Csonka and Fraenkel, 1977; Eliot and Kirsch, 2004), as well as relative acidity of α-hydrogen positions; thus, this NADPH-derived hydrogen is not propagated to proline in our schematic. Acidity of hydrogen in aldehyde positions may result in additional loss of NADPH hydrogen transferred in step 3b, although tritium labeling experiments support retention of this hydrogen through synthesis of proline (Csonka and Fraenkel, 1977). Enzymes: 1a, citrate synthase; 2-3a, aconitase; 4-5a, isocitrate dehydrogenase; 1b, glutamate dehydrogenase; 2b, glutamate-5-kinase; 3b, γ-glutamyl phosphate reductase; 4b, spontaneous cyclization; 5b, 1-pyrroline-5-carboxylate reductase. See Fig. S20 for distribution of isozymes across organisms investigated in this study.

## 7.2 Phenylalanine biosynthesis

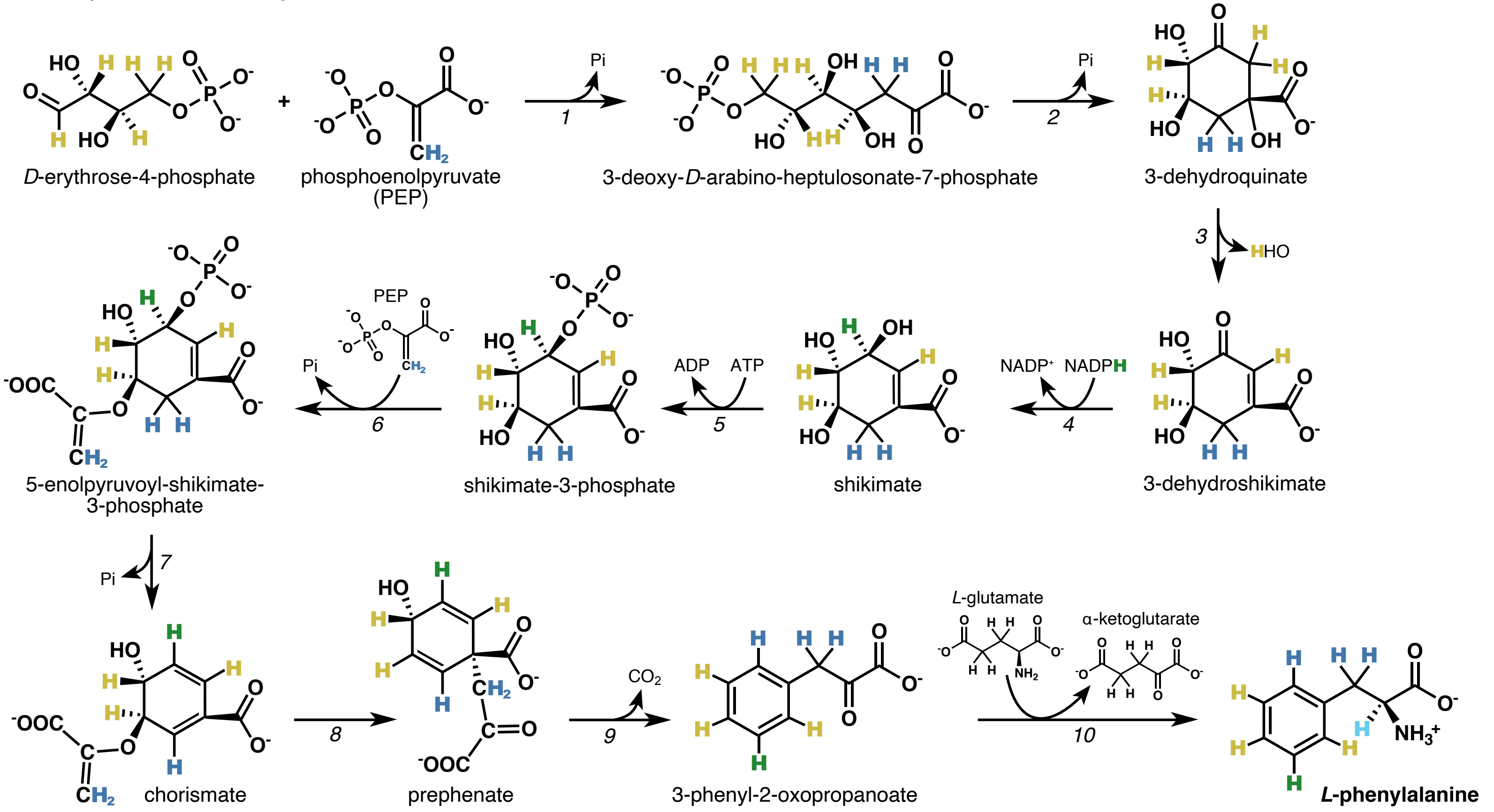


**Figure S17.** General biosynthetic pathway of phenylalanine with colors representing tracked hydrogen sources (yellow for erythrose-4-phosphate, dark blue for PEP, green for NADPH, light blue for water). During step 6, hydrogen in the incoming PEP substrate may be $^2$H-enriched during its condensation with shikimate-3-phosphate, as the methylene group of PEP is transiently converted to a methyl group in the enzyme-bound intermediate, then deprotonated with an accompanying normal KIE (Grimshaw et al., 1982). This hydrogen atom eventually becomes the C3 hydrogen in phenylalanine. Although steps 3 and 7 result in abstraction of one proton from a pair, these reactions likely do not contribute to phenylalanine's $^2$H-enrichment due to their stereospecificity (Turner et al., 1975; Vaz, 1980; Onderka and Floss, 1969; Bornemann et al., 2000). PLP-dependent enzyme reactions equilibrate the α-hydrogen in amino acids with solvent (Eliot and Kirsch, 2004). Enzymes: 1, 3-deoxy-7-phosphoheptulonate synthase; 2, 3-dehydroquinate synthase; 3, 3-dehydroquinate dehydratase; 4, shikimate dehydrogenase; 5, shikimate kinase; 6, 3-phosphoshikimate 1-carboxyvinyltransferase; 7, chorismate synthase; 8, chorismate mutase; 9, prephenate dehydratase; 10, phenylalanine transaminase/ aromatic aminotransferase. See Fig. S20 for distribution of isozymes across organisms investigated in this study.

### 7.3 Leucine and valine biosynthesis

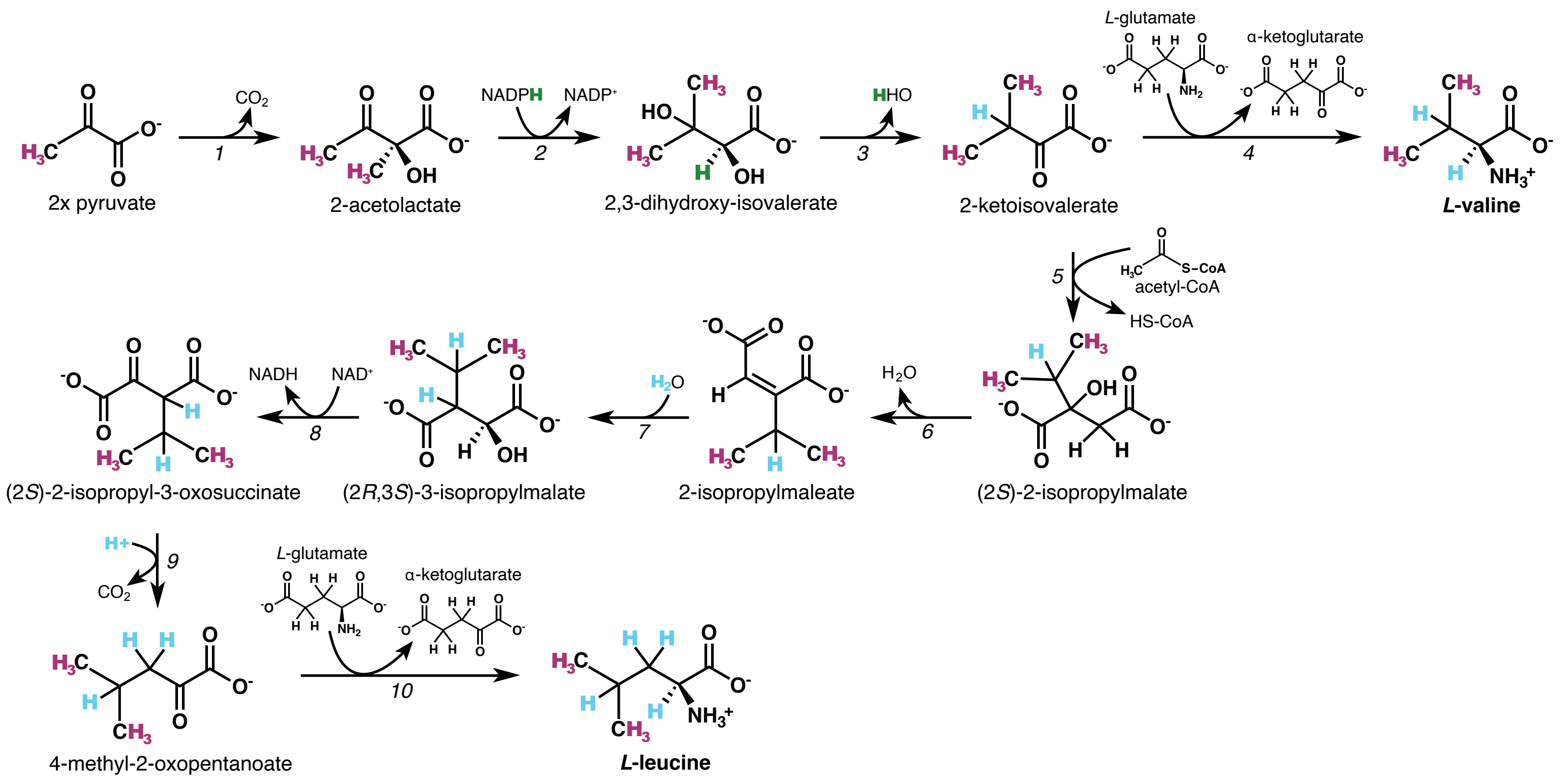


**Figure S18.** General biosynthetic pathways of leucine and valine with colors representing tracked hydrogen sources (purple for pyruvate, green for NADPH, light blue for water). PLP-dependent enzyme reactions equilibrate the α-hydrogen in amino acids with solvent (Eliot and Kirsch, 2004). Enzymes: 1, acetolactate synthase; 2, ketol-acid reductoisomerase; 3, dihydroxy-acid dehydratase; 4, valine transaminase/ branched-chain amino acid aminotransferase; 5, 2-isopropylmalate synthase; 6-7, 3-isopropylmalate dehydratase; 8, 3-isopropylmalate dehydrogenase; 9, spontaneous rearrangement; 10, leucine aminotransferase/ branched-chain amino acid aminotransferase. See Fig. S20 for distribution of isozymes across organisms investigated in this study.

## 7.4 Isoleucine biosynthesis

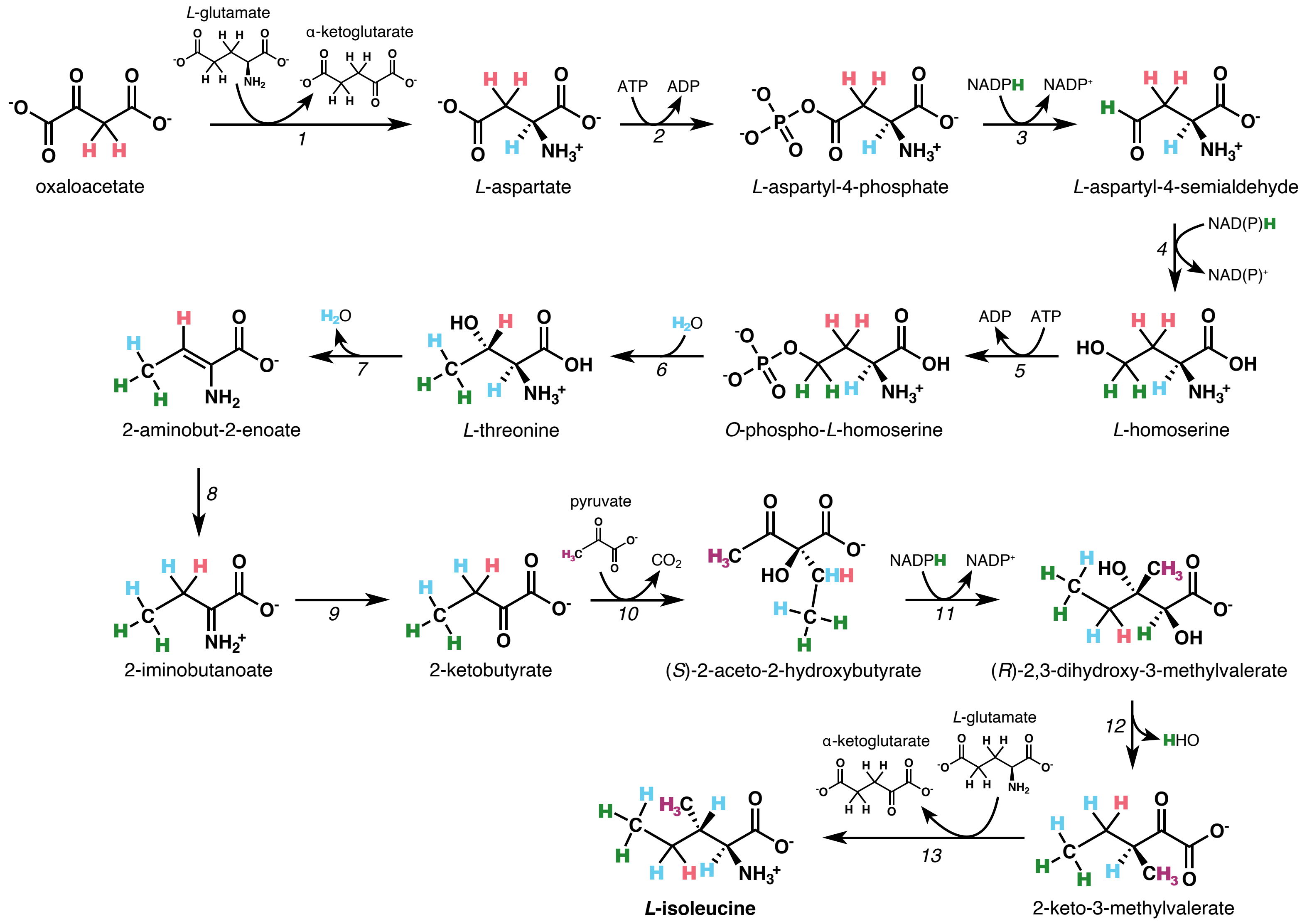


**Figure S19.** General biosynthetic pathway of isoleucine with colors representing tracked hydrogen sources (red for oxaloacetate, light blue for water, green for NADPH, purple for pyruvate). The hydrogen atom transferred from NADPH in step 3 may equilibrate with water due to its acidity in an aldehyde position, but is shown as retained through isoleucine synthesis here. Threonine synthase (step 6) stereospecifically abstracts the *pro-R* hydrogen from phosphohomoserine (Omi et al., 2003); in turn, this *pro-R* hydrogen originates from fumarate (while the

*pro-S* hydrogen is water-derived) when oxaloacetate is synthesized through fumarase and malate dehydrogenase activity (Gawron and Fondy, 1959). The methylene hydrogen in oxaloacetate and aspartyl-4-semialdehyde may equilibrate with water through keto-enol tautomerizations (Kosicki, 1962; Bruice and Bruice, 1978), although the extents of these equilibrations depend on the rates of subsequent transformation reactions (e.g., the rate of aspartyl-4-semialdehyde conversion to homoserine). PLP-dependent enzyme reactions equilibrate the α-hydrogen in amino acids with solvent (Eliot and Kirsch, 2004). Enzymes: 1, aspartate transaminase; 2, aspartate kinase; 3, aspartate semialdehyde dehydrogenase; 4, homoserine dehydrogenase; 5, homoserine kinase; 6, threonine synthase; 7, threonine deaminase; 8-9, spontaneous rearrangement; 10, acetohydroxybutanoate synthase; 11, ketol-acid reductoisomerase; 12, dihydroxyacid dehydratase; 13, isoleucine transaminase/ branched-chain amino acid aminotransferase. See Fig. S20 for distribution of isozymes across organisms investigated in this study.

## 7.5 Distribution of amino acid biosynthetic enzymes across organisms

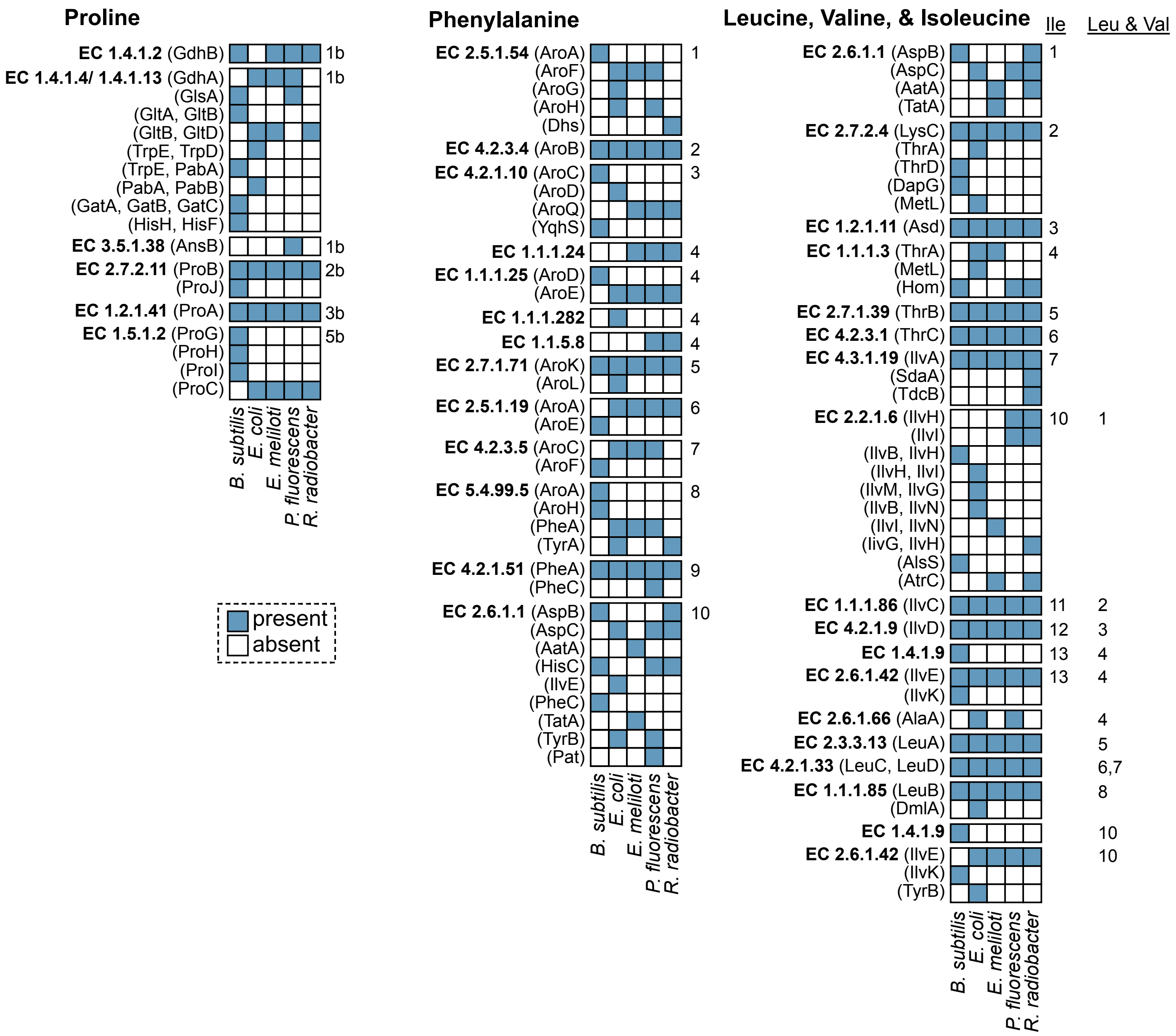

**Figure S20.** Distribution of enzymes in amino acid biosynthetic pathways across organisms investigated in this study. Reactions are denoted by their EC numbers (bold text), with numbers to the right of each grid row denoting the step in the biosynthetic pathway (Figs. S16-S19) where the enzyme is found. Leucine, valine, and isoleucine have overlapping biosynthetic pathways, so are displayed as one set of grids, with numbers to the right additionally denoting steps corresponding to leucine + valine versus isoleucine pathways. Proteins are annotated in parentheses as single or multi-subunit complexes. All proteins corresponding with a given reaction are listed in the grid block next to or under their respective EC number. The presence/absence of each enzyme in each organism was determined using a combination of BsubCyc, EcoCyc (Keseler et al., 2021), BioCyc (Karp et al., 2019), and KEGG pathway (Kanehisa et al., 2016) databases. For *E. meliloti* and *R. radiobacter*, reference genomes used in BioCyc were *'Sinorhizobium meliloti 1021 reference genome'* and *'Agrobacterium fabrum C58 reference genome,'* respectively. Organism databases referenced for KEGG pathway searches were 'Bacillus subtilis subsp. subtilis 168 [bsu],' 'Escherichia coli K-12 MG1655 [eco],' 'Sinorhizobium meliloti 1021 [sme],' and 'Agrobacterium fabrum [atu]' for *B. subtilis, E. coli, E. meliloti* (formerly *S. meliloti*), and *R. radiobacter* (syn. *Agrobacterium fabrum*). For *P. fluorescens*, no BioCyc or KEGG pathway database included a strain sufficiently genetically similar to our strain (average nucleotide identity cutoff score >95%), so KEGG Orthologs were assigned to GenBank protein sequences in *P. fluorescens* strain 2-79 (BioSample: SAMN03278127) using KofamKOALA (Aramaki et al., 2020), and enzymes were identified using KEGG pathway maps.

## REFERENCES


Aramaki, T., Blanc-Mathieu, R., Endo, H., Ohkubo, K., Kanehisa, M., Goto, S., and Ogata, H. (2020). KofamKOALA: KEGG Ortholog assignment based on profile HMM and adaptive score threshold. *Bioinformatics* 36, 2251–2252. doi:10.1093/bioinformatics/btz859

Bigeleisen, J. (1965). Chemistry of Isotopes. *Science* 147, 463–471. doi:10.2172/4014119

Bornemann, S., Theoclitou, M.-E., Brune, M., Webb, M. R., Thorneley, R. N., and Abell, C. (2000). A secondary β deuterium kinetic isotope effect in the chorismate synthase reaction. *Bioorganic Chemistry* 28, 191–204. doi:10.1006/bioo.2000.1174

Bruice, P. Y. and Bruice, T. C. (1978). Lack of concertedness in the catalysis of the enolization of oxaloacetic acid by general acids and bases. Formation of a carbinolamine intermediate in the tertiary amine catalyzed enolization reaction. *Journal of the American Chemical Society* 100, 4793–4801. doi:10.1021/ja00483a027

Corr, L. T., Berstan, R., and Evershed, R. P. (2007). Optimisation of derivatisation procedures for the determination of $\delta^{13}C$ values of amino acids by gas chromatography/combustion/isotope ratio mass spectrometry. *Rapid Communications in Mass Spectrometry* 21, 3759–3771. doi:10.1002/rcm.3252

Csonka, L. N. and Fraenkel, D. G. (1977). Pathways of NADPH formation in *Escherichia coli*. *The Journal of Biological Chemistry* 252, 3382–3391

Dolan, S. K., Kohlstedt, M., Trigg, S., Ramirez, P. V., Kaminski, C. F., Wittmann, C., et al. (2020). Contextual flexibility in *Pseudomonas aeruginosa* central carbon metabolism during growth in single carbon sources. *American Society for Microbiology* 11, e02684–19. doi:10.1128/mBio.02684-19

Eliot, A. C. and Kirsch, J. F. (2004). Pyridoxal Phosphate Enzymes: Mechanistic, Structural, and Evolutionary Considerations. *Annual Review of Biochemistry* 73, 383–415. doi:10.1146/annurev.biochem.73.011303.074021

Englander, S. W. and Poulsen, A. (1969). Hydrogen-tritium exchange of the random chain polypeptide. Biopolymers 7, 379–393

Fogel, M. L., Griffin, P. L., and Newsome, S. D. (2016). Hydrogen isotopes in individual amino acids reflect differentiated pools of hydrogen from food and water in *Escherichia coli*. *Proceedings of the National Academy of Sciences* 113, E4648–E4653. doi:10.1073/pnas.1525703113

Gawron, O. and Fondy, T. P. (1959). Stereochemistry of the fumarase and aspartase catalyzed reactions and of the krebs cycle from fumaric acid to *d*-isocitric acid. *Journal of the American Chemical Society* 81, 6333–6334

Gerosa, L., Haverkorn van Rijsewijk, B., Christodoulou, D., Kochanowski, K., Schmidt, T., Noor, E., et al. (2015). Pseudo-transition analysis identifies the key regulators of dynamic metabolic adaptations from steady-state data. *Cell Systems* 1, 270–282. doi:10.1016/j.cels.2015.09.008

Grimshaw, C., Sogo, S., and Knowles, J. (1982). The fate of the hydrogens of phosphoenolpyruvate in the reaction catalyzed by 5-enolpyruvylshikimate-3-phosphate synthase. Isotope effects and isotope exchange. *Journal of Biological Chemistry* 257, 596–598. doi:10.1016/S0021-9258(19)68232-3

Hill, J. and Leach, S. J. (1964). Hydrogen exchange at carbon-hydrogen sites during acid or alkaline treatment of proteins. *Biochemistry* 3, 1814–1818. doi:10.1021/bi00900a003

Hušek, P. (1991b). Rapid derivatization and gas chromatographic determination of amino acids. *Journal of Chromatography A* 552, 289–299. doi:10.1016/S0021-9673(01)95945-X

Kanehisa, M., Sato Y., Kawashima, M., Furumichi, M., and Tanabe, M. (2016). KEGG as a reference resource for gene and protein annotation. *Nucleic Acids Research* 44, D457–D462.

Karp, P. D., Billington, R., Caspi, R., Fulcher, C. A., Latendresse, M., Kothari, A., Keseler, I. M., Krummenacker, M., Midford, P. E., Ong, Q., Ong, W. K., Paley, S. M., and Subhraveti, P. (2019). The BioCyc collection of microbial genomes and metabolic pathways. *Briefings in Bioinformatics* 20, 1085–1093. doi:10.1093/bib/bbx085

Keseler, I. M., Gama-Castro, S., Mackie, A., Billington, R., Billington, R., Caspi, R., Kothari, A., Krummenacker, M., Midford, P., Muñiz-Rascado, L., Ong, W., Paley, S., Santos-Zavaleta, A., Subhraveti, P., Tierrafria, V., Wolfe, A., Collado-Vides, J., Paulsen, I., and Karp, P. D. (2021). The EcoCyc database in 2021. *Frontiers in Microbiology* 12, 711077.

Kopf, S. H. (2014). From lakes to lungs: assessing microbial activity in diverse environments. Ph.D. thesis, California Institute of Technology.

Kosicki, G. W. (1962). Isotope rate effects in the enolization of oxalacetic acid. *Canadian Journal of Chemistry* 40, 1280–1284. doi:10.1139/v62-196

Kreuzer-Martin, H. W., Lott, M. J., Ehleringer, J. R., and Hegg, E. L. (2006). Metabolic processes account for the majority of the intracellular water in log-phase *Escherichia coli* cells as revealed by hydrogen isotopes. *Biochemistry* 45, 13622–13630. doi:10.1021/bi0609164

Lowenstein, J. M. (1967). The tricarboxylic acid cycle. In Metabolic Pathways, ed. D. M. Greenberg (New York: Academic Press), vol. 1. 146–270

Ochs, R. S. and Talele, T. T. (2020). Revisiting prochirality. *Biochimie* 170, 65–72. doi:10.1016/j.biochi.2019.12.009

Omi, R., Goto, M., Miyahara, I., Mizuguchi, H., Hayashi, H., Kagamiyama, H., et al. (2003). Crystal structures of threonine synthase from *Thermus thermophilus* HB8. *The Journal of Biological Chemistry* 278, 46035–46045.

Onderka, D. K. and Floss, H. G. (1969). Steric course of the chorismate synthetase reaction and the 3-deoxy-D-arabino-heptulosonate 7-phosphate (DAHP) synthetase reaction. *Journal of the American Chemical Society* 91, 5894–5896. doi:10.1021/ja01049a046

Phillips, A. A., Wu, F., and Sessions, A. L. (2021). Sulfur isotope analysis of cysteine and methionine via preparatory liquid chromatography and elemental analyzer isotope ratio mass spectrometry. *Rapid Communications in Mass Spectrometry* 35, e9007. doi:10.1002/rcm.9007

Sessions, A. L. and Hayes, J. M. (2005). Calculation of hydrogen isotopic fractionations in biogeochemical systems. *Geochimica et Cosmochimica Acta* 69, 593–597. doi:10.1016/j.gca.2004.08.005

Silverman, S. N., Phillips, A. A., Weiss, G. M., Wilkes, E. B., Eiler, J. M., and Sessions, A. L. (2022). Practical considerations for amino acid isotope analysis. *Organic Geochemistry* 164, 104345. doi:10.1016/j.orggeochem.2021.104345

Smith, W. G. and York, J. L. (1970). Stereochemistry of the citric acid cycle. *Journal of Chemical Education* 47, 588. doi:10.1021/ed047p588

Turner, M. J., Smith, B. W., and Haslam, E. (1975). The Shikimate Pathway. Part IV. The Stereochemistry of the 3-Dehydroquinate Dehydratase Reaction and Observations on 3-Dehydroquinate Synthetase. *Journal of the Chemical Society, Perkin Transactions 1* 1, 52–55.

Vaz, A. D. N. (1980). Studies on the enzymatic cis dehydration of 3-dehydroquinic acid catalyzed by *Escherichia coli* 3-dehydroquinate dehydratase. Ph.D. thesis, Tulane University.

Walsh, R. G., He, S., and Yarnes, C. T. (2014). Compound-specific $\delta^{13}C$ and $\delta^{15}N$ analysis of amino acids: a rapid, chloroformate-based method for ecological studies. *Rapid Communications in Mass Spectrometry* 28, 96–108. doi:10.1002/rcm.6761

Wijker, R. S., Sessions, A. L., Fuhrer, T., and Phan, M. (2019). $^{2}H/^{1}H$ variation in microbial lipids is controlled by NADPH metabolism. *Proceedings of the National Academy of Sciences* 116, 12173–12182

Wright, H. T. (1991). Nonenzymatic deamidation of asparaginyl and glutaminyl residues in proteins. *Critical Reviews in Biochemistry and Molecular Biology* 26, 1–52.

Zhang, X., Gillespie, A. L., and Sessions, A. L. (2009). Large D/H variations in bacterial lipids reflect central metabolic pathways. *Proceedings of the National Academy of Sciences* 106, 12580–12586. doi:10.1073/pnas.0903030106
